# Selective CO_2_ Electroreduction to CO by an Organometallic Nickel Catalyst Featuring a C_3_–Symmetric Tris(Phosphino)Alkyl Ligand

**DOI:** 10.1021/acscatal.5c08299

**Published:** 2026-02-04

**Authors:** Sergio Fernández, Klaudia Michaliszyn, Ekaterina S. Smirnova, Marc Robert, Josep M. Luis, Julio Lloret-Fillol

**Affiliations:** † Institute of Chemical Research of Catalonia (ICIQ), The Barcelona Institute of Science and Technology, Avinguda Països Catalans 16, Tarragona 43007, Spain; ‡ 27063 Sorbonne Université, CNRS, Institut Parisien de Chimie Moléculaire, IPCM, Paris F-75005, France; § Institut de Química Computacional i Catàlisi (IQCC) and Departament de Química, Universitat de Girona, Campus Montilivi, Girona, Catalonia E-17003, Spain; ∥ Catalan Institution for Research and Advanced Studies (ICREA), Passeig Lluïs Companys, 23, Barcelona 08010, Spain

**Keywords:** CO_2_ reduction, electrocatalysis, reaction mechanisms, cyclic voltammetry, organometallic chemistry, nickel

## Abstract

We report a Nickel CO_2_ reduction electrocatalyst based on a *C*_3_-symmetric tris­(phosphino)­alkyl ligand, CNPPh_3_, which displays a metalated axial carbon atom. Catalyst **Ni** ^**H**^_**Br**_ selectively reduces CO_2_ to CO (FY_CO_ = 94%) at −2.3 V vs Fc^+/0^ with a TO *F*_max_ = 65 s^–1^ in DMF/[TBA]­PF_6_ with 3.5 M of added H_2_O. Cyclic voltammetry (CV) and an exhaustive computational study of the reaction mechanism show that our Ni^II^ complex undergoes two one-electron reduction events before the CO_2_ binding step. Afterward, the catalytic CO_2_ reduction takes place through a reduction-first pathway. The formation of a Ni–CO intermediate along the CO_2_ reduction pathway was inferred by CV, and the corresponding [Ni^II^–CO]^+^ complex was isolated. FTIR spectroelectrochemistry (SEC) allowed for the detection of three different Ni–CO species: [Ni–CO]^+^, [Ni–CO]^0^, and [Ni–CO]^−^. This work provides critical insights into the electrocatalytic CO_2_ reduction, laying the foundation for efficient CO_2_ conversion strategies.

## Graphical abstract

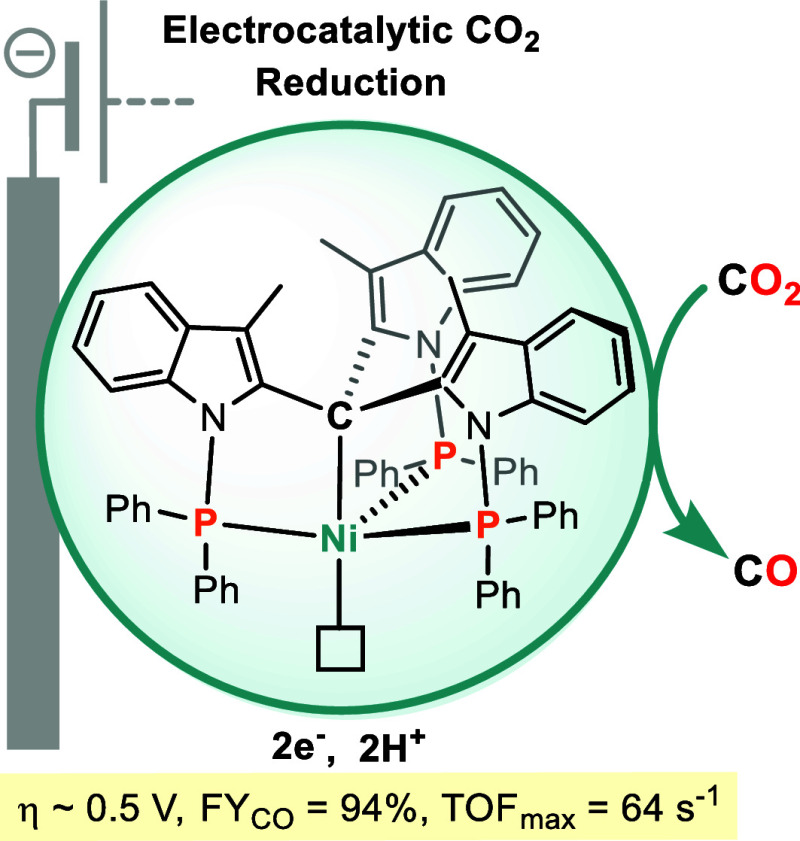

## Introduction

The current increasing energy demand has led to a rising interest in developing new and more effective methods for renewable energy conversion and storage.^1^ The conversion of solar energy into chemical energy through an electrical potential is one of the most promising approaches for that purpose. In this circular economy context, CO_2_ has become a target molecule to utilize as a C_1_ building block in the synthesis of fuels and fine chemicals.^2^ However, the geometry and electronic structure of CO_2_ make it stable against electrophiles, and its one-electron reduction occurs at highly negative potentials.^3,4^ Consequently, the multielectron/multiproton CO_2_ reduction to fuels needs to be catalyzed.^5^

Heterogeneous catalysts are envisioned as the most suitable ones for large-scale CO_2_ reduction in water.^6^ They can also provide access to multiple products, ranging from CO to C_n ≥ 2_ products, although selectivity is still a challenge.^7^ Recently, well-defined molecular complexes have been demonstrated to reduce CO_2_ beyond two electrons under photochemical,^8,9^ electrochemical,^10–14^ and photoelectrochemical^15^ conditions. Also, they have shown promising results when coupled with organometallic catalysis in solution to produce MeOH and organic products.^16,17^ Although heterogenized molecular catalysts can offer improved durability and catalytic performance,^11,18–21^ the well-defined nature of molecular catalysts in solution is an advantage for their investigation by electrochemical and spectroscopic techniques.^22^ In this regard, the CO_2_-to-CO reduction mechanisms have been studied extensively during the last years, allowing for the identification of the thermodynamic and kinetic factors governing the CO_2_ reduction reaction.^23–33^ We believe these studies are crucial to establishing the fundamentals for developing faster and more selective catalysts as well as novel reactivity.^31,32^

In the particular case of Ni complexes, only a few ligand platforms have provided good catalytic performance (Chart 1).^6,34^ Most of them are based on macrocyclic ligands like cyclam derivatives (**Ni1–2**), phthalocyanines (**Ni3**), or non-macrocyclic (amino)­pyridine and related ligands (**Ni4–6**). Among them, adsorbed (**Ni1**) or supported (**Ni3**) catalysts show excellent performance in aqueous electrolyte. On the contrary, most homogeneous Ni catalysts present much lower selectivity toward CO production (Table 1). FTIR-SEC studies on **Ni1** and **Ni5** complexes allowed to identify the one electron reduction of [Ni–CO]^+^ as a key intermediate in the catalytic cycle.^35,36^

**Chart 1:**
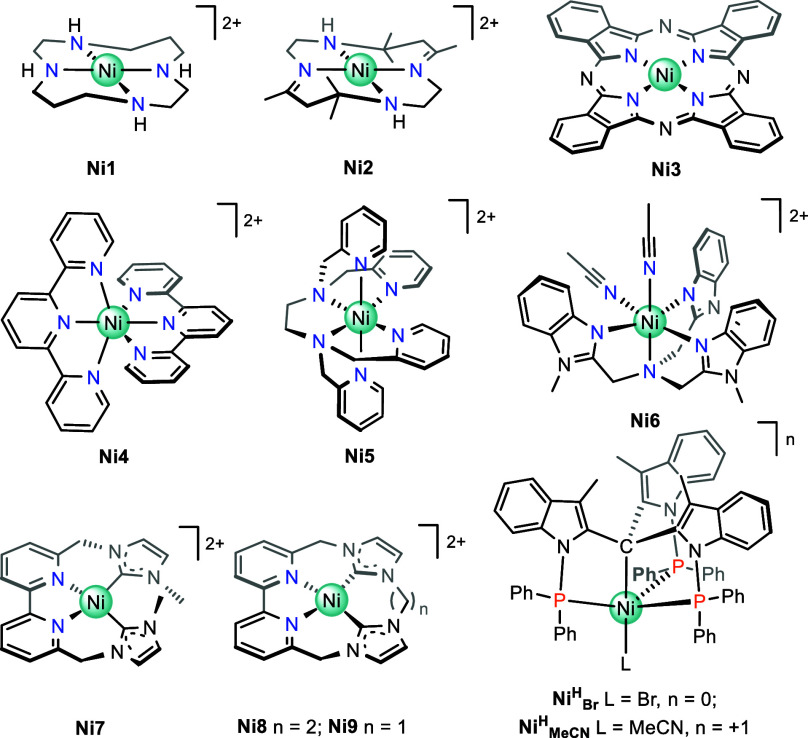
Structures of selected Ni molecular CO_2_-to-CO reduction electrocatalysts.^35–41^

**Table 1:** CO_2_-To-CO Reduction Performance (FY for CO) of Selected Ni Reduction Electrocatalysts (Ni1-9)

Cat	conditions	E(V vs Fc^+/0^)^c^	FY (%)
Ni1^a^	H_2_O (pH = 4.1)	–1.68	96
Ni2	-	–1.89	-
Ni3^b^	H_2_O (0.5 M KHCO_3_)	–1.63 to −2.38	100
Ni4	DMF (5% H_2_O)	–1.72 to −2.14	17
Ni5	MeCN (0.5 M PhOH)	–2.05	-
Ni6	DMF (5 M H_2_O)	–1.87	96
Ni7	MeCN (2% H_2_O)	–2.25	5
Ni8	MeCN (2% H_2_O)	–2.4	56
Ni9	MeCN (2% H_2_O)	–2.4	87

a Adsorbed on a Hg pool electrode.

b Supported over a gas diffusion electrode.

c E (V vs Fc^+/0^) = *E* (V vs NHE) −0.63 = *E* (V vs SCE) −0.39 ^43^.

Selective CO_2_-to-CO electroreduction based on organometallic Nickel catalysts are rare compared to other catalyst families.^34^ Among them, complexes based on NHC ligands have shown electrocatalytic activity for the CO_2_ reduction to CO.^42–46^ In this regard, Jurss, Panetier, and co-workers reported the electrochemical reduction of CO_2_ to CO catalyzed by a family of *bis-*NHC Ni complexes (**Ni7**-**9**). This study shows that the catalyst becomes more selective for the CO_2_ reduction to CO as the rigidity of the macrocyclic ligand is increased.^41^

Herein, we report the selective electrocatalytic CO_2_-to-CO reduction with a unique organometallic Nickel complex (**Ni** ^**H**^_**Br**_ in Chart 1) based on a *C*_*3*_ *-*symmetric tris­(phosphino)­alkyl ligand (C^N^P^Ph^_3_).^47,48^ The synthesis, characterization and catalytic activity of **Ni** ^**H**^_**Br**_ in the thermal hydrogenation of *N-*heteroarenes has been recently reported by our group.^49^ Interestingly, complex **Ni** ^**H**^_**Br**_ displays a metalated axial carbon atom, and this is reminiscent of the interstitial C atom in the iron–molybdenum cofactor of Mo-dependent nitrogenase that exhibits CO_2_ reduction activity under certain conditions.^50^ Peters and co-workers have reported similar tris­(phosphino)­silyl Ni complexes which can bind N_2_, H_2_, and CO.^51,52^ To the best of our knowledge, **Ni** ^**H**^_**Br**_ is the first trigonal-bipyramidal complex with an axial alkyl ligand and three equatorial phosphines exhibiting CO_2_ reduction catalytic activity.

## Results and Discussion

In addition to **Ni** ^**H**^_**Br**_, we have prepared the corresponding MeCN solvento complex [**Ni** ^**H**^_**MeCN**_]­BF_4_ (see Supporting Information for details). This catalyst also displays a trigonal-bipyramidal geometry and shows higher solubility than **Ni** ^**H**^_**Br**_. The labile character of the MeCN ligand makes **Ni** ^**H**^_**MeCN**_ a more suitable complex for the mechanistic study by electrochemical and spectroscopic techniques (see Figure 1).

**Figure 1:**
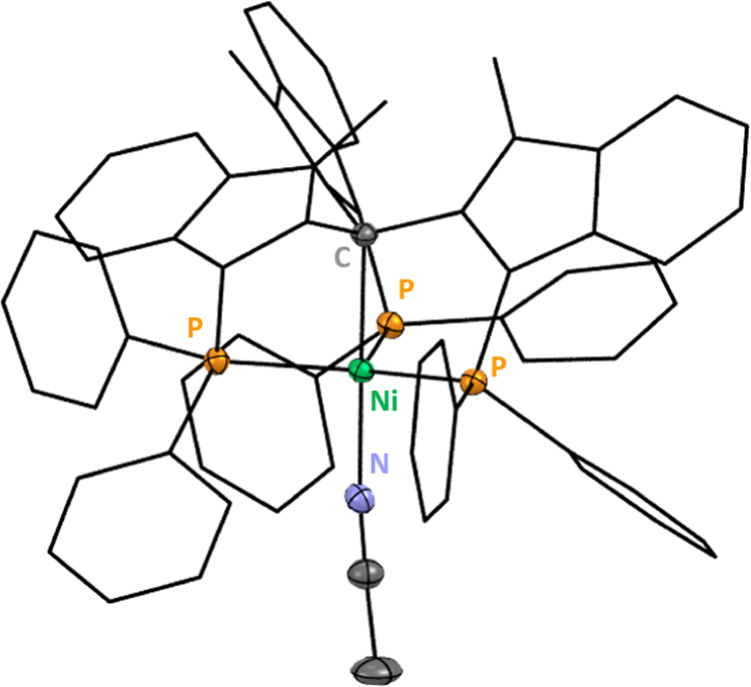
ORTEP plot of **Ni** ^**H**^_**MeCN**_, thermal ellipsoids at 50% probability. Bond lengths (Å): d­(Ni–P) = 2.24–2.26; d­(Ni–C) = 2.05; d­(Ni–N) = 1.93. H atoms, anions and solvent molecules are omitted for clarity.

### Electrochemistry under Argon

The cyclic voltammogram (CV) of **Ni** ^**H**^_**Br**_ in DMF/[TBA]­PF_6_ (0.1 M) solvent/electrolyte mixture under an inert atmosphere presents two main features upon reductive scan (Figure 2). The first one is irreversible, with *E*_p,c_ −1.46 V, and the second one is fully reversible and centered at −1.64 V (all redox potentials are referenced to Fc^+/0^). Upon increasing the scan rate, the first irreversible reduction peak shifts cathodically (Figure S5). A progressive cathodic shift is also observed with the addition of increasing amounts of tetrabutylammonium bromide (Figure S6). This experimental evidence is in line with a ligand exchange process involving the Br^–^ elimination after the electrochemical reduction.

**Figure 2:**
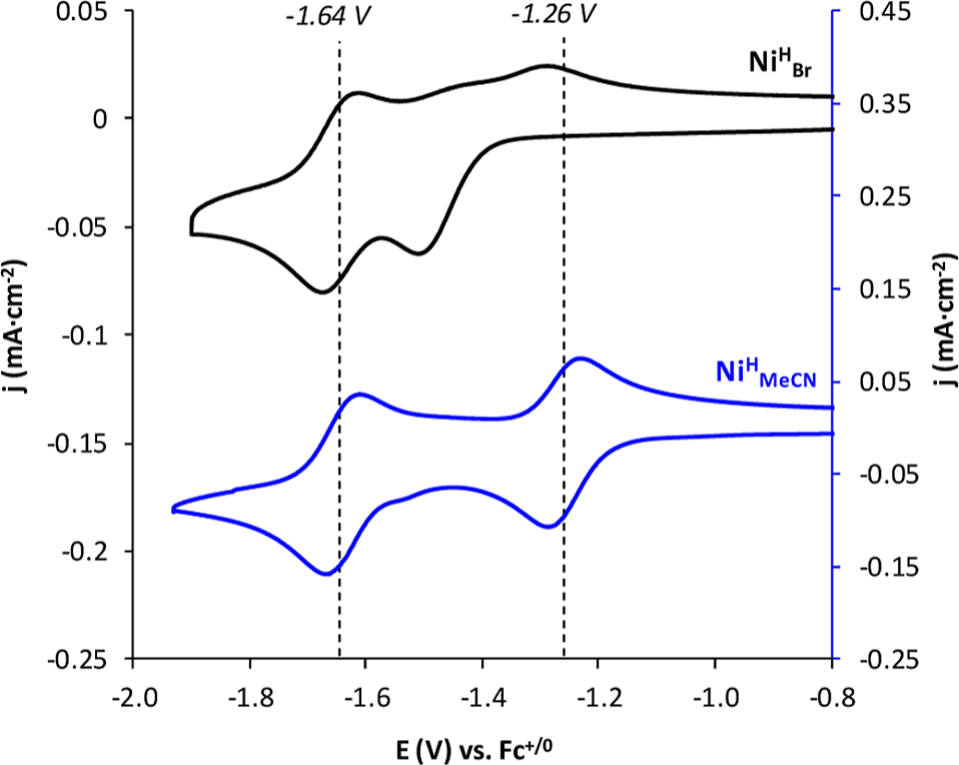
CVs of **Ni** ^**H**^_**Br**_ (black) and **Ni** ^**H**^_**MeCN**_ (blue) under argon in DMF/[TBA]­PF_6_ (0.1 M). [Ni] = 0.5 mM, *v* = 0.1 V·s^–1^.

Interestingly, the CV of the solvento complex **Ni** ^**H**^_**MeCN**_ exhibits two fully reversible one-electron reduction waves under Argon atmosphere. The first reduction takes place at an *E*_1/2_ = −1.26 V. This redox event appears 200 mV more positive than the first reduction of **Ni** ^**H**^_**Br**_, most likely due to the cationic nature of **Ni** ^**H**^_**MeCN**_. Instead, the second reversible process appears at an *E*_1/2_ = −1.64 V overlapping perfectly with the second reduction described for **Ni** ^**H**^_**Br**_ (Figure 2). In this case, the two features observed by CV have been assigned to a Ni^II/I^ and a Ni^I/0^ redox couple, respectively.

By comparing the electrochemistry of **Ni** ^**H**^_**Br**_ and **Ni** ^**H**^_**MeCN**_ under an inert atmosphere, we conclude that the irreversible process observed for **Ni** ^**H**^_**Br**_ is consistent with an electrochemical-chemical (EC) mechanism. This EC mechanism involves the Br^–^ dissociation from the 19-electron [Ni^I^–Br]^−^ species to form a 17-electron [Ni^I^]^0^ intermediate (eq 1). The latter species is oxidized to the corresponding [Ni^II^]^+^ species at the anodic back scan of the CV (eq 2). In contrast, the second reduction corresponds to a one-electron reversible process (Δ*E*_p_ = 60 mV, *E*_1/2_ = −1.64 V) which is in line with the formation of the [Ni^0^]^−^ 18-electron species from [Ni^I^]^0^ (eq 3). The mechanistic proposal is supported by the two diffusive reversible electrochemical processes observed (*E*_1/2_ = −1.26 and −1.64 V) for **Ni** ^**H**^_**MeCN**_ in DMF solution (Figure S8).

1 $${{[Ni}^{II}-Br]}^{0}\overset{{+e}^{-}}{\to }{{[Ni}^{I}-Br]}^{-}{{\overset{{-Br}^{-}}{\to }[Ni}^{I}]}^{0},E_{p,c}=-1.46V$$

2 $${\left[{Ni}^{I}\right]}^{0}\overset{{-e}^{-}}{\to }{\left[{Ni}^{II}\right]}^{+}\overset{{+Br}^{-}}{\to }{{[Ni}^{II}-Br]}^{0},E_{p,a}=-1.26V$$

3 $${\left[{Ni}^{I}\right]}^{0}+e^{-}⇌{\left[{Ni}^{0}\right]}^{-},E_{1/2}=-1.64V$$

### Electrochemistry under CO_2_

In CO_2_-saturated electrolyte, **Ni** ^**H**^_**Br**_ undergoes remarkable changes upon reductive scan, suggesting reactivity between CO_2_ and the electrochemically generated species (Figure 3A). While the first wave suffers a minor change, the second wave shifts to a more positive potential and becomes irreversible. This interaction becomes more evident from the CV in the presence of added H_2_O, suggesting the formation of a metal-carboxylate species (eq 4) that could be potentially detected by in situ FTIR spectroelectrochemistry (SEC).

4 $$,{\left[{Ni}^{0}\right]}^{-}+{CO}_{2}\overset{k_{CO2}}{\to }{{[Ni}^{II}{CO}_{2}]}^{-}\overset{H^{+}}{\to }{[Ni}^{II}{CO}_{2}H]$$

**Figure 3:**
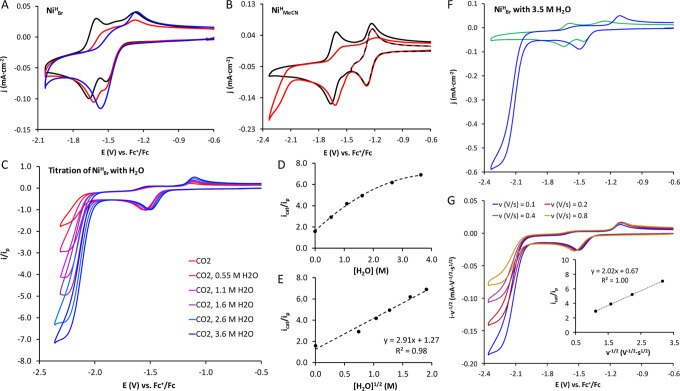
(A) CVs of **Ni** ^**H**^_**Br**_ under Argon (black) and CO_2_ in dry electrolyte (red) and with 0.55 M H_2_O (blue). (B) CVs of **Ni** ^**H**^_**MeCN**_ under Argon (black) and CO_2_ (red) in MeCN 0.1 M [TBA]­PF_6_. (C) CVs of **Ni** ^**H**^_**Br**_ under CO_2_ at increasing [H_2_O]. (D) *i*_cat_/*i*_p_ vs [H_2_O]. (E) *i*_cat_/*i*_p_ vs the square root of [H_2_O]. (F) CVs of **Ni** ^**H**^_**Br**_ with H_2_O (3.5 M) under Argon (green) and CO_2_ (blue). (G) Scan rate-normalized CV under CO_2_ (3.5 M H_2_O) at 0.1–0.8 V·s^–1^ and *i*_cat_/*i*_p_ vs v^–1/2^ plot (inset). General conditions: [Ni] = 0.5 mM in DMF/[TBA]­PF_6_ 0.1 M and 0.1 V·s^–1^ unless otherwise indicated.

Equation 5 relates the peak potential shift measured at a certain scan rate (υ) with the kinetic constant (*k*_CO2_) of the chemical reaction in an EC mechanism at a certain CO_2_ concentration ([CO_2_] = 0.2 M in DMF).^53,54^ In the case of **Ni** ^**H**^_**MeCN**_, a 17 mV shift of the peak potential is observed with respect to the thermodynamic Ni^I/0^ redox couple, leading to *k*_CO2_(Ni^0^) = 250 M^–1^ s^–1^ in DMF.

5 $$E_{p}=E_{1/2}\left({Ni}^{I/0}\right)-0.78\frac{RT}{F}+\frac{RT}{2F}\ln\frac{RTk_{CO2}\left[{CO}_{2}\right]}{F\upsilon }$$

The small change observed in the first reduction may also result from an overlap with the second reduction process. To deconvolute this effect, the CV of **Ni** ^**H**^_**MeCN**_ was recorded in CO_2_-saturated anhydrous MeCN (Figure 3B). The CV at 100 mV/s showed full reversibility and nearly identical features under both N_2_ and CO_2_, effectively ruling out any interaction between [Ni^I^]^0^ and CO_2_ on the CV time scale. However, since SEC operates on a slower time scale, it allows for the potential observation of such interactions by coupling electroreduction with in situ detection of carboxylate intermediates. Although the C=O stretching region is complicated by the presence of carbonate (ν_CO_ = 1646 cm^–1^) and bicarbonate (ν_CO_ = 1684 cm^–1^), a distinct band was observed at 1677 cm^–1^ (Figure S25). This matches the DFT-computed ν_CO_ values of the [Ni–CO_2_H]^0^ (1673 cm^–1^) intermediate (Figure S26), suggesting the formation of a {Ni–CO_2_} adduct that undergoes protonation with adventitious H_2_O.

Under CO_2_ atmosphere, complex **Ni** ^**H**^_**Br**_ starts to show a current increase in the CV below −2.0 V. The addition of small amounts of H_2_O led to the progressive current increase, which is in line with a proton-dependent catalytic process (Figure 3C). At *E*_cat_ = −2.27 V, an *S-*shaped catalytic response is observed with a 7-fold increase in current after the addition of 3.5 M of H_2_O (Figure 3C). The same behavior is observed for the **Ni** ^**H**^_**MeCN**_ in both DMF (Figures S9 and S10) and MeCN (Figure S14). CV experiments in MeCN with added H_2_O (3%_v/v_) allowed for the estimation of a catalytic overpotential (η) of ca. 500 mV considering that *E*_cat/2_ is −2.05 V under these conditions.^55^ Control experiments in the absence of CO_2_ show no catalytic current increase upon the addition of a proton source, ruling out a major contribution of the catalytic reduction of water protons to H_2_ (Figure S11). This behavior suggests that the current increase observed upon the addition of H_2_O is due to the catalytic CO_2_ reduction.

Titration studies reveal that the catalytic process is first order with respect to H_2_O concentration, as evidenced by the linear relationship between the normalized catalytic current (*i*_cat_/*i*_p_) and the square root of the H_2_O concentration, where H_2_O acts as a Brønsted acid (Figure 3D,E). The same current increase is observed when D_2_O is added instead of H_2_O, indicating the absence of a kinetic isotope effect (KIE_H/D_ ≈1, Figure S12), despite the proton-assisted nature of the CO_2_ reduction reaction. Other proton sources, such as 2,2,2-trifluoroethanol, also activate the catalytic CO_2_ reduction wave with a first-order dependence, displaying an equivalent behavior to the one found with added H_2_O (Figure S13).

Controlled potential electrolysis (CPE) under CO_2_ atmosphere at *E*_app_ = −2.3 V revealed the formation of CO as the major product with a Faradaic yield (FY) of 94% after 1.8 h (Figure S15). Given the outstanding selectivity for CO, and considering that **Ni** ^**H**^_**Br**_ operates under pure kinetic conditions, a *k*_obs_ = 64 s^–1^ was obtained by the analysis of the catalytic wave at increasing scan rates using eq 6 (Figure 3F,G)

6 $$\frac{i_{cat}}{i_{p}}=\frac{1}{0.446}\sqrt{\left(\frac{RT}{nFv}\right)n^{\prime }k_{obs}}$$

being *n* = 2 the number of ET occurring at the electrode per equivalent of catalyst, and *n*’ = 1 the reaction order in the catalyst.^56,57^ *k*_obs_, also known as TO*F*_max_, is the observed first-order kinetic constant that depends linearly on the substrate concentration (eq 7). Under CO_2_ saturation, the intrinsic catalytic rate constant (*k*_cat_) is 350 s^–1^.

7 $$k_{obs}=k_{cat}\left[{CO}_{2}\right]$$

The fact that the catalytic reaction initiates 0.32 V more negative than the Ni^*I*/0^ reduction suggests the catalytic cycle proceeds through a reduction-first pathway (eq 8).^58^ In the anodic back scan after the catalytic wave, a new feature is observed at *E*_p,a_ = −1.12 V. Consistent with previous mechanistic studies with other molecular Fe, Co, and Ni electrocatalysts, we suggest that this anodic feature is due to the oxidation of a {Ni–CO} species (vide infra).^24,35,59^

8 $${[Ni}^{II}{CO}_{2}H]\overset{e^{-}}{\to }{{[Ni}^{I}{CO}_{2}H]}^{-}\overset{H^{+}}{\to }{{[Ni}^{I}CO]}^{0}+H_{2}O$$

Although SEC studies in dry acetonitrile allowed for the detection of CO_3_^2–^/HCO_3_^–^ evolving at catalytically relevant potentials, no {Ni–CO} species were identified in the spectral window between 2100 and 1850 cm^–1^ (Figure S23).

### Electrochemistry under CO

To test our hypothesis and to obtain more information about the {Ni–CO} reoxidation, we run the CV of **Ni** ^**H**^_**Br**_ in CO-saturated DMF (Figure 4A). Under these conditions, the CV response of **Ni** ^**H**^_**Br**_ presents an irreversible reduction at *E*_p,c_ = −1.50 V (eq 9) followed by a reversible wave at *E*_1/2_ = −1.66 V (eq 10). The anodic peak at *E*_p,a_ = −1.12 V is also observed, as under CO_2_ saturation in the presence of H_2_O (Figures S16 and S17). This result is in line with the detection of a [Ni^I^–CO] intermediate, which is oxidized to [Ni^II^–CO]^+^ at the anodic back scan of the CV. After this oxidation, a consecutive cathodic scan (0.1 V·s^–1^) allowed to observe the reversible Ni^II/I^–CO process at *E*_1/2_ = −1.14 V (eq 11, Figure 4A). The partial loss of reversibility of this wave at lower scan rates indicates that the electrochemically generated [Ni^II^–CO]^+^ engages the irreversible CO dissociation (eq 12). Using the working curve method to scrutinize the dependency of the Ni^II/I^–CO reduction peak current on the scan rate, we were able to estimate a CO dissociation constant of 0.05 ± 0.02 s^–1^ from [Ni^II^–CO]^+^ (see Section S4.2 of the Supporting Information).^26,60,61^

9 $${[Ni}^{II}Br]+e^{-}\overset{{-Br}^{-}}{\to }{\left[{Ni}^{I}\right]}^{-}{\overset{CO}{\to }[Ni}^{I}CO],E_{p,c}=-1.50V$$

10 $${[Ni}^{I}CO]+e^{-}\to {{[Ni}^{0}CO]}^{-},E_{1/2}=-1.66V$$

11 $${[Ni}^{I}CO]\to {{[Ni}^{II}CO]}^{+}+e^{-},E_{1/2}=-1.14V$$

12 $${{[Ni}^{II}CO]}^{+}\overset{+{Br}^{-}}{\to }{[Ni}^{II}Br]+CO,k_{-CO}=0.05\pm 0.02s^{-1}$$

**Figure 4:**
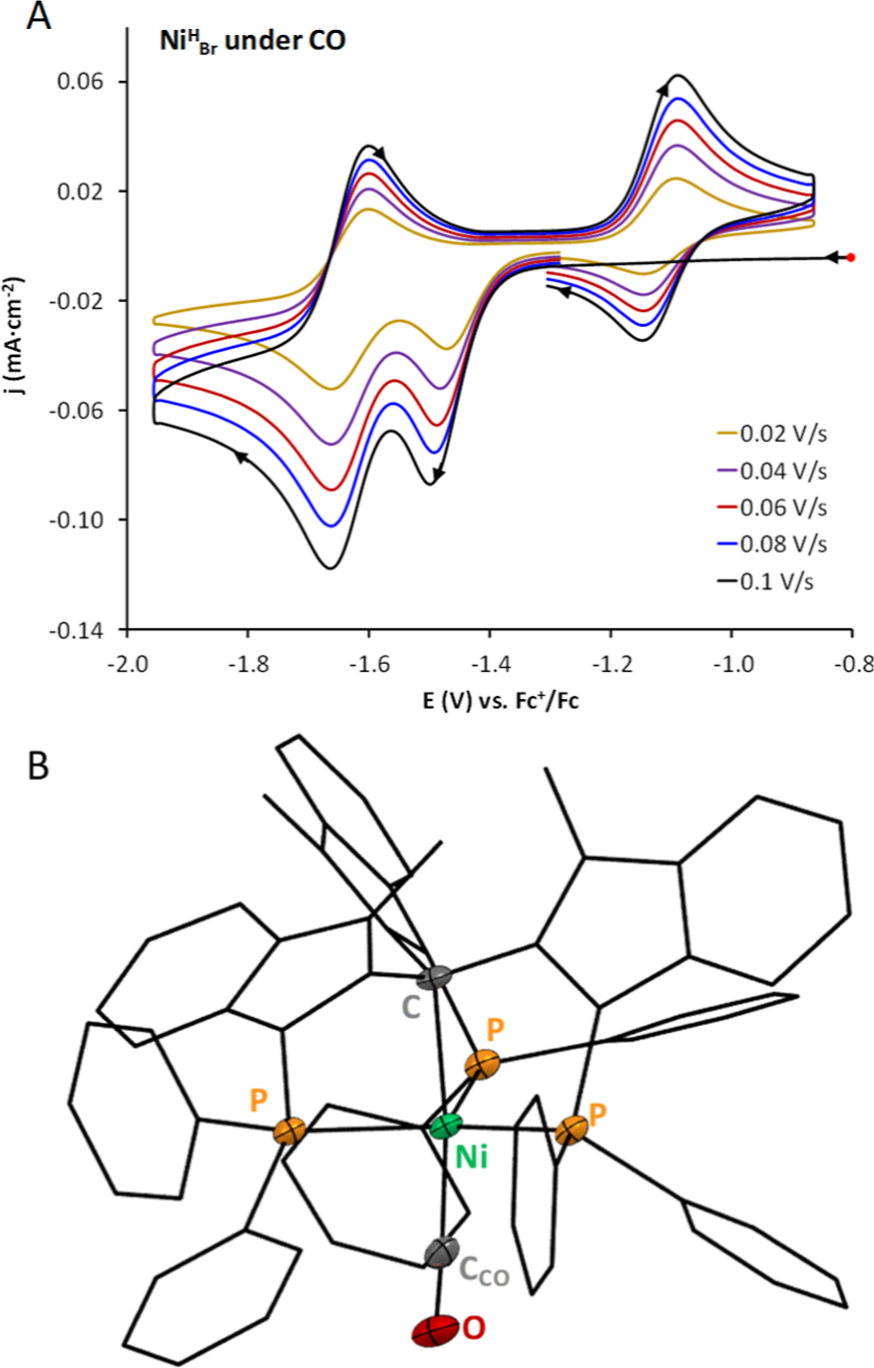
(A) CVs of **Ni** ^**H**^_**Br**_ under CO at increasing scan rates. (B) ORTEP plot of the **Ni** ^**H**^_**CO**_ solid state structure. Hydrogen atoms, anions and solvent molecules have been omitted for clarity. Relevant bond lengths: d­(Ni–P) = 2.23–2.27 Å; d­(Ni–C) = 2.10 Å; d­(Ni–C_CO_) = 1.80 Å; d­(C_CO_–O) = 1.14 Å.

Starting from a solution of **Ni** ^**H**^_**MeCN**_ in a noncoordinating solvent, we could synthesize and fully characterize the corresponding [Ni^II^–CO]­BF_4_ ([**Ni** ^**H**^_**CO**_]­BF_4_) complex (Scheme 1). Under 1 atm of CO, the ligand exchange was complete after 3 h under CO atmosphere, as suggested by ^1^H- and ^31^P NMR (Section S2 of the Supporting Information). Slow diffusion of hexane into a solution of (**Ni** ^**H**^_**CO**_)­BF_4_ in CH_2_Cl_2_ gave suitable crystals for X-ray diffraction (Figure 4B, Section S3 of the Supporting Information). The strong trans effect of the CO ligand is reflected in the elongation of the Ni–C bond (d­(Ni–C) = 2.10 Å) with respect to **Ni** ^**H**^_**MeCN**_ (d­(Ni–C) = 2.05 Å). Both **Ni** ^**H**^_**MeCN**_ and **Ni** ^**H**^_**CO**_ show a single signal in ^31^P NMR, at 60.10 and 69.51 ppm, respectively, which agrees with the *C*_*3*_-symmetry of the two complexes.

**Scheme 1:**
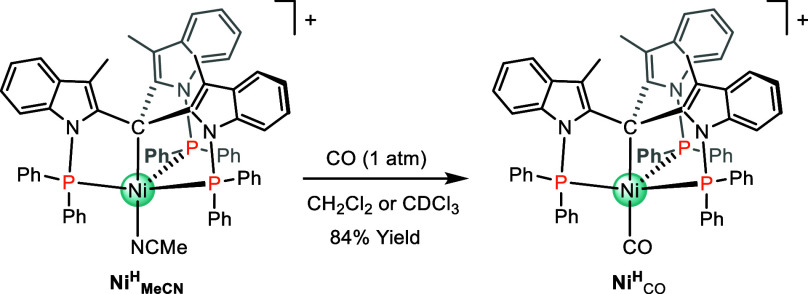
Synthesis of **Ni** ^**H**^_**CO**_

The FTIR spectrum of **Ni** ^**H**^_**CO**_ in MeCN solution shows an intense absorption at 2060 cm^–1^, which is in the region of C–O stretching frequencies of metal–carbonyls (Figure 5A). In 2012, Peters and co-workers reported two similar trigonal-bipyramidal cationic Ni^II^–CO complexes based on tris­(phosphino)­silyl ligands.^50^ These complexes display a metalated silyl ligand in trans position to the CO coordination site. Despite this difference, the Ni–C_CO_ and C_CO_–O bond distances obtained from X-ray diffraction are close to those of **Ni** ^**H**^_**CO**_. Nonetheless, the ν_CO_ of the two previously reported complexes appears at a lower energy than the one of **Ni** ^**H**^_**CO**_, suggesting that the coordination of CO is weaker in our tris­(phosphino)­alkyl Ni^II^–CO complex.

**Figure 5:**
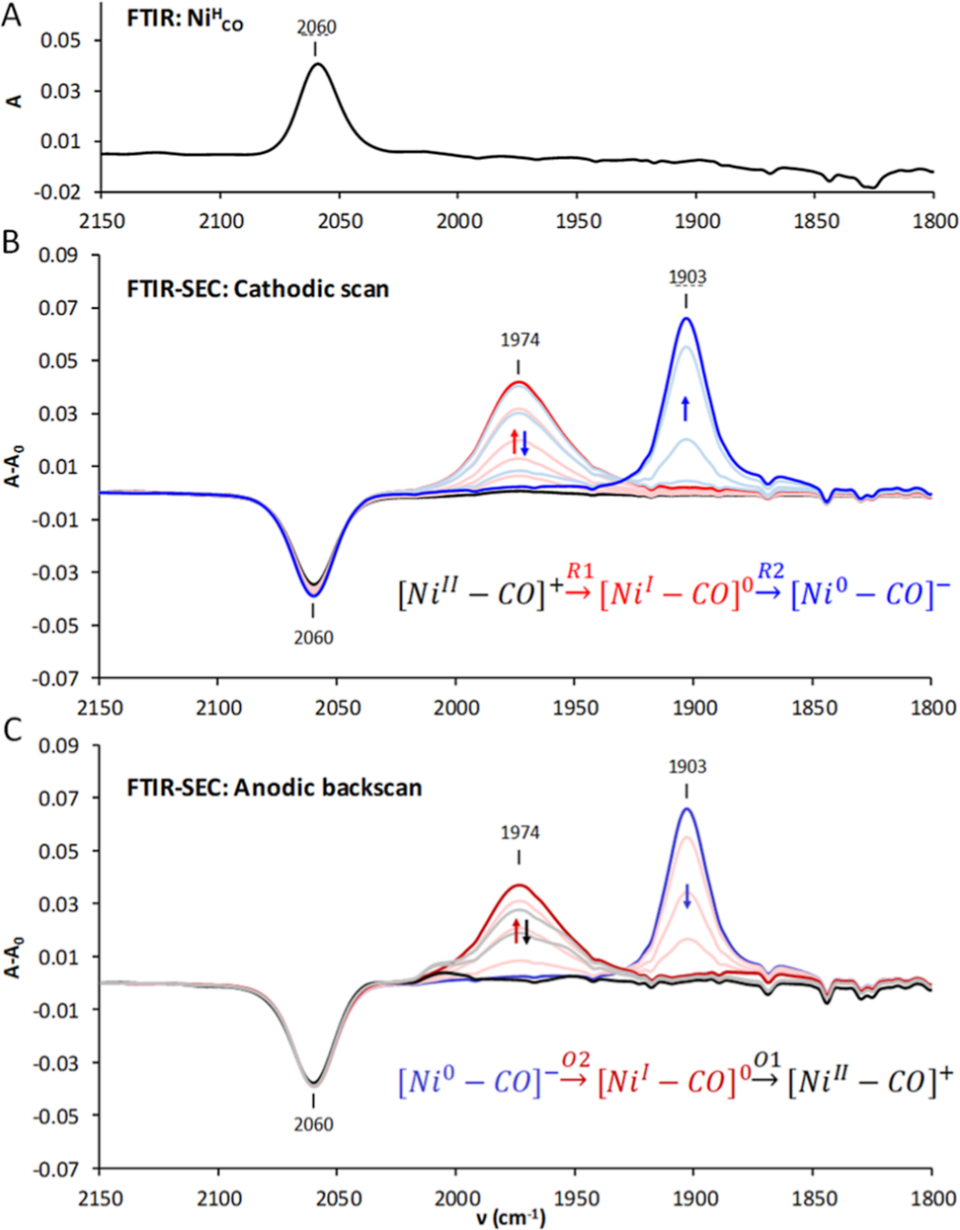
(A) FTIR of **Ni** ^**H**^_**CO**_ (4 mM) in MeCN/[TBA]­PF_6_ (0.2 M). (B) Followed FTIR-SEC differential spectra upon cathodic scan (R1 in red, R2 in blue). (C) Followed FTIR-SEC differential spectra of the anodic back scan (O1 in red, O2 in blue). The negative band at 2060 cm^–1^ is the consequence of a fast redox autocatalyzed CO, MeCN exchange.

The CV of **Ni** ^**H**^_**CO**_ shows two reversible processes, the first one centered at *E*_1/2_(Ni^II/I^–CO) = −1.16 V is 100 mV earlier than the *E*_1/2_(Ni^II/I^) process of **Ni** ^**H**^_**MeCN**_ (Figures S19 and S20). This agrees with the strong π-backbonding of the CO ligand, which contributes to stabilizing the Ni^I^ center. Conversely, the *E*_1/2_(Ni^I/0^–CO) process occurs close to the Ni^I/0^ wave of **Ni** ^**H**^_**MeCN**_ at −1.66 V. The CV of **Ni** ^**H**^_**CO**_ under CO_2_ with added water (3.5 M) shows the same catalytic response as **Ni** ^**H**^_**MeCN**_ and **Ni** ^**H**^_**Br**_, meaning that neither the coordinated CO nor the Br^–^ ligands inhibit the catalytic process (Figures S21 and S22).

To investigate the nature of the reduced Ni–CO intermediates, we performed in situ FTIR-SEC experiments. We started from a 4 mM solution of [**Ni** ^**H**^_**CO**_]­BF_4_ in MeCN/[TBA]­PF_6_ (0.2 M) under an inert atmosphere. At the open circuit potential, the ν_CO_ of [Ni^II^–CO]^+^ is observed at 2060 cm^–1^ (Figure 5A). However, this feature suddenly disappears once the applied potential matches the onset potential of the first reduction (R1, Figure 5B). This behavior is consistent with a redox-triggered depletion of the Ni^II^–CO.

starting complex upon one-electron reduction. Accordingly, the signal of the parent Ni^II^–CO species appears as a negative band centered at 2060 cm^–1^ in the differential spectra, reflecting consumption of the initial complex. Under these reducing conditions, the resulting Ni^I^–CO intermediate can engage in a redox-catalyzed solvent exchange process, in which substitution of CO by MeCN is facilitated by electron transfer (Scheme 2).^28^

**Scheme 2:**
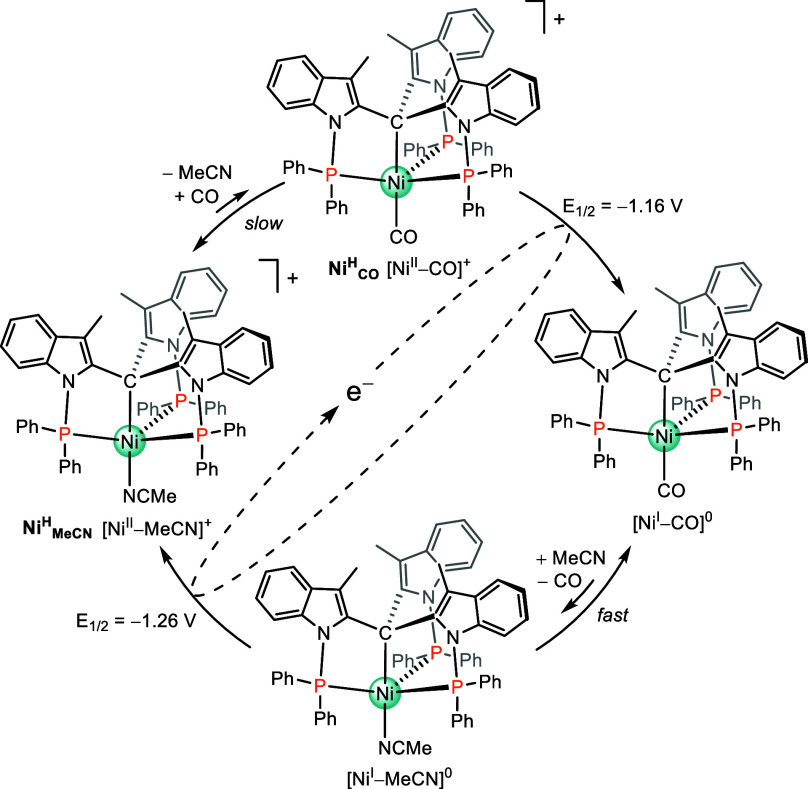
Proposed Redox-Catalyzed Ligand Exchange Process for **Ni** ^**H**^_**CO**_ ^a^

a Considering that the **[Ni** ^**I**^ **–CO]** ^**0**^ is less reducing than the **[Ni** ^**I**^ **–MeCN]** ^**0**^, a fast CO, MeCN ligand exchange at the Ni­(I) oxidation state, the formation of the **[Ni** ^**II**^ **–MeCN]** ^**+**^ is redox catalyzed at the **[Ni** ^**II/I**^ **–CO]** ^**+/0**^ onset redox potential.

Then, upon cathodic scan, a new signal appears at 1974 cm^–1^ throughout the first reduction process (R1, red) as shown in Figure 5B. At the second reduction (R2, blue), the 1974 cm^–1^ signal evolves to a new absorption at 1903 cm^–1^, showing an isosbestic point. We propose that these two new features correspond to [Ni^I^–CO] and [Ni^0^–CO]^−^, respectively. The latter (ν_CO_ = 1903 cm^–1^) is reversibly oxidized to the former (ν_CO_ = 1974 cm^–1^) in the anodic reverse scan (O2) (Figure 5C). Finally, the carbonyl feature disappeared at the last oxidation process (O1), which implies that the **Ni** ^**H**^_**MeCN**_ is formed after the FTIR-SEC experiment. These results suggest the clean formation of nickel–carbonyl species in the II, I, and 0 formal oxidation states with no signs of catalyst decomposition.

### Computational Mechanistic Study

To rationalize the catalytic activity and support our experimental data, we have computed the CO_2_ reduction mechanism by DFT. In this case, **Ni** ^**H**^_**MeCN**_ was selected as model to exclude the computationally problematic negatively charged species such as the isolated Br^–^ anion. The B3LYP hybrid functional together with the 6–31G* basis set were employed for the optimization and frequency calculation of the different intermediates, as Peters and co-workers did for a Ni complex also based on a C_3_-symmetric triphosphine ligand.^52^ We also included Grimme-D_3_ dispersion and the SMD implicit solvation model for MeCN. The Gibbs energy of each intermediate has been calculated as the sum of the electronic energy (*E*_elec_) obtained from a single point calculation at the MN15/6–311++G** level of theory on each optimized structure and the Gibbs energy correction (dG_corr_) from the previous B3LYP-D_3_/6–31G* frequency calculation (eq 13).

13 $$G=E_{elec}\left(MN15/6-311++G^{**}\right)+{dG}_{corr}\left(B3LYP/6-31G^{*}\right)$$

The Gibbs energy profile in Figure 6 shows the reduction-first pathway calculated at the theoretical *E*_1/2_ (Ni^0/–^) = −2.1 V potential. The reaction starts with the formation of the catalytically active species. First, the [Ni^II^–MeCN]^+^ species is reduced to [Ni^I^–MeCN]^0^ with a theoretical *E*_1/2_ (Ni^+/0^) = −1.32 V. That first redox event is followed by the dissociation of the MeCN ligand, which is endergonic by 4.3 kcal·mol^–1^. Then, a second reduction event generates the anionic active intermediate [Ni^0^]^−^ from the tetracoordinate [Ni^I^]^0^ species. While the DFT Ni^II/I^ redox potential qualitatively matches the experimental one, the DFT redox potential of the Ni^I/0^ couple differs substantially from the experimental value (Table 2). The discrepancy between the theoretical and experimental redox potential of the second reduction can be due to the limitations of the DFT methods in the description of anionic species. Nonetheless, we have explored different DFT functionals and concluded that the Ni^0/–^ reduction potential is highly dependent on the DFT method, obtaining the best results with MN15 (Table S4).

**Figure 6:**
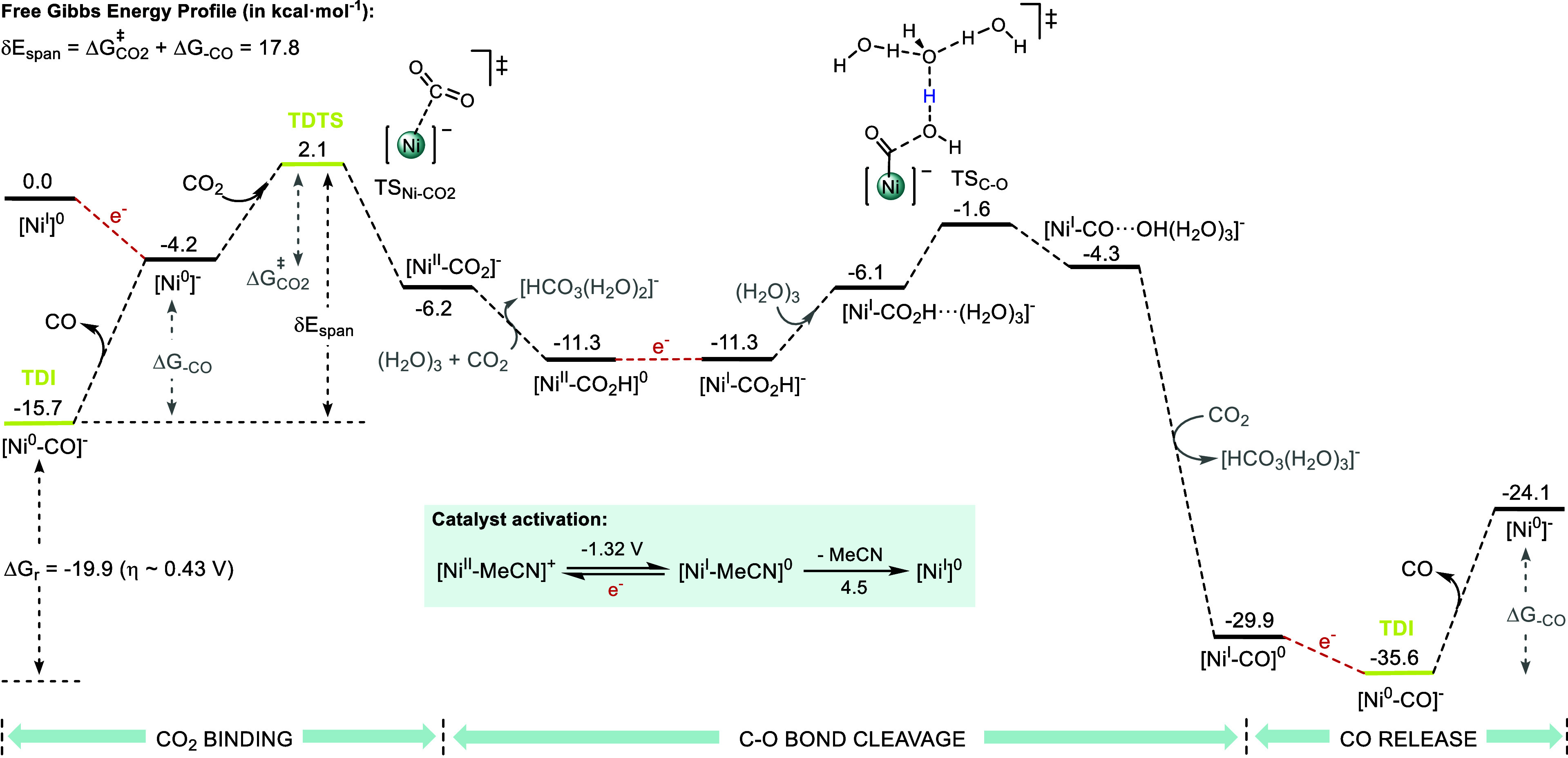
DFT computed Gibbs energy (kcal·mol^–1^) profile for the CO_2_ reduction to CO with complex catalyzed by **Ni** ^**H**^_**MeCN**_ computed at the MN15/6–311++G**//B3LYP-D_3_/6–31G* level of theory. TDI and TDTS are shown in yellow and reduction events in red. *E*_app_ = −2.1 V vs Fc^+/0^; [H_2_O]_3_ = [HCO_3_(H_2_O)_2_]^−^ = 1.2 M; [CO_2_] = 0.28 M; [CO] = 0.028 M; L = MeCN.

**Table 2:** Comparison between Experimental and DFT (MN15/6-311++G**//B3LYP-D_3_/6-31G*) Reduction Potentials, *E*_O*x*/Red_ (V vs Fc^+/0^)

oxidized	reduced	*E*_exp_ (V)	E_DFT_ (V)
[Ni^II^–MeCN]^+^	[Ni^I^–MeCN]^0^	–1.26	–1.32
[Ni^I^]^0^	[Ni^0^]^−^	–1.64	–1.92
[Ni^II^–CO_2_H]^0^	[Ni^I^–CO_2_H]^−^	*nd*	–2.10
[Ni^II^–CO]^+^	[Ni^I^–CO]^0^	–1.16	–1.24
[Ni^I^–CO]^0^	[Ni^0^–CO]^−^	–1.66	–1.86

The CO_2_ binding to the two-electron reduced intermediate [Ni^0^]^−^ is exergonic and requires overcoming a low kinetic barrier (Δ*G* ^‡^_CO2_ = 6.3 kcal·mol^–1^). Once the Ni-carboxylate intermediate is formed, the first protonation event to form the [Ni^II^–CO_2_H]^0^ intermediate is downhill in energy. To model the proton transfer events, we have considered an explicit cluster of three water molecules as the proton source. The buffering effect of CO_2_ has been accounted for in both the kinetics and thermodynamics of the energy profile by considering the reactivity of the OH^–^–water cluster resulting from proton transfer events with CO_2_ to give a HCO_3_^–^–water cluster.^32^ This strategy allows for the stabilization of the OH^–^ species, which is essential for providing a better description of the thermodynamics of the protonation of the carboxylate intermediates.^62^

Following a reduction first pathway, [Ni^II^–CO_2_H]^0^ is reduced to [Ni^I^–CO_2_H]^−^ before the C–O bond cleavage step. At the selected redox potential for the representation of the energy profile (−2.1 V), the latter two species are isoenergetic. Then, the protonation of [Ni^I^–CO_2_H]^−^ triggers the C–O bond cleavage through an energy barrier of 9.7 kcal·mol^–1^. Although the dissociation of a hydroxide-water cluster [OH­(H_2_O)_3_]^−^ is endergonic, the reaction becomes downhill in energy when the formation of [Ni^0^–CO]^−^ is accompanied by the formation of a solvated hydrogen carbonate ion cluster from CO_2_ and [OH­(H_2_O)_3_]^−^. Then, the subsequent Ni^li/0^–CO reduction is also thermodynamically favored. Finally, the catalytic cycle is closed after the endergonic CO release to give [Ni^0^]^−^ (Δ*G*_–CO_ = 11.5 kcal·mol^–1^). At −2.1 V, the catalytic CO_2_-to-CO reduction ΔG_r_ is exergonic by −19.9 kcal·mol^–1^, leading to a DFT-estimated overpotential of 430 mV, which is in very good agreement with the one observed experimentally (η = 500 mV).

Considering the energetic span model,^63^ the energetic span (δE_span_) of a Gibbs energy profile can be determined according to eq 14, which depends on the relative position of the TOF determining TS (TDTS) and the TOF determining intermediate (TDI)

14 $${\delta E}_{span}=\{\begin{array}{l} G_{TDTS}-G_{TDI},TDTS\;afterTDI\\ G_{TDTS}-G_{TDI}+{\Delta G}_{r},TDTS\;beforeTDI \end{array}$$

In the energy profile depicted in Figure 6, [Ni^0^–CO]^−^ and TS_Ni‑CO2_ are the TDI and the TDTS, respectively. Considering that the TDTS appears earlier than the TDI, the barrier is calculated by adding the energy difference between the TDTS and the TDI and the thermodynamics of the overall reaction (Δ*G*_r_). Then, the calculated δE_span_ is 17.8 kcal·mol^–1^, consistent with an obtained kinetic constant of 64 s^–1^.

### Mechanistic Discussion

The synergistic combination of experimental and computational results leads to the mechanistic proposal depicted in Scheme 3. Starting from **Ni** ^**H**^_**Br**_, the first irreversible reduction involves the elimination of the Br^–^ ligand to form a [Ni^I^]^0^ species. Then, the reversible [Ni^I^]^0^/[Ni^0^]^−^ takes place at −1.62 V. Under a CO_2_ atmosphere, the pentacoordinate [Ni^0^]^−^ evolves to [Ni^II^–CO_2_]^−^. From the cathodic shift observed in the CV, we have estimated a CO_2_ binding constant to Ni^0^ (*k*_CO2_) of 250 M^–1^ s^–1^ in anhydrous solvent. In the presence of a Brønsted acid, [Ni–CO_2_]^−^ gets protonated giving [Ni–CO_2_H]^0^. Subsequently, this intermediate needs to be further reduced to trigger the catalysis at an onset potential of −2.0 V (Figure 3F), which is in line with a reduction-first mechanism. This is also in agreement with our DFT study, since the computed [Ni–CO_2_H]^0/–^ redox potential of −2.1 V matches the experimental *E*_cat/2_ = −2.05 V. Subsequent protonation cleaves the C–O bond generating [Ni^I^–CO]^0^ which releases CO upon one-electron reduction and regenerates [Ni^0^]^−^ closing the catalytic cycle.

**Scheme 3:**
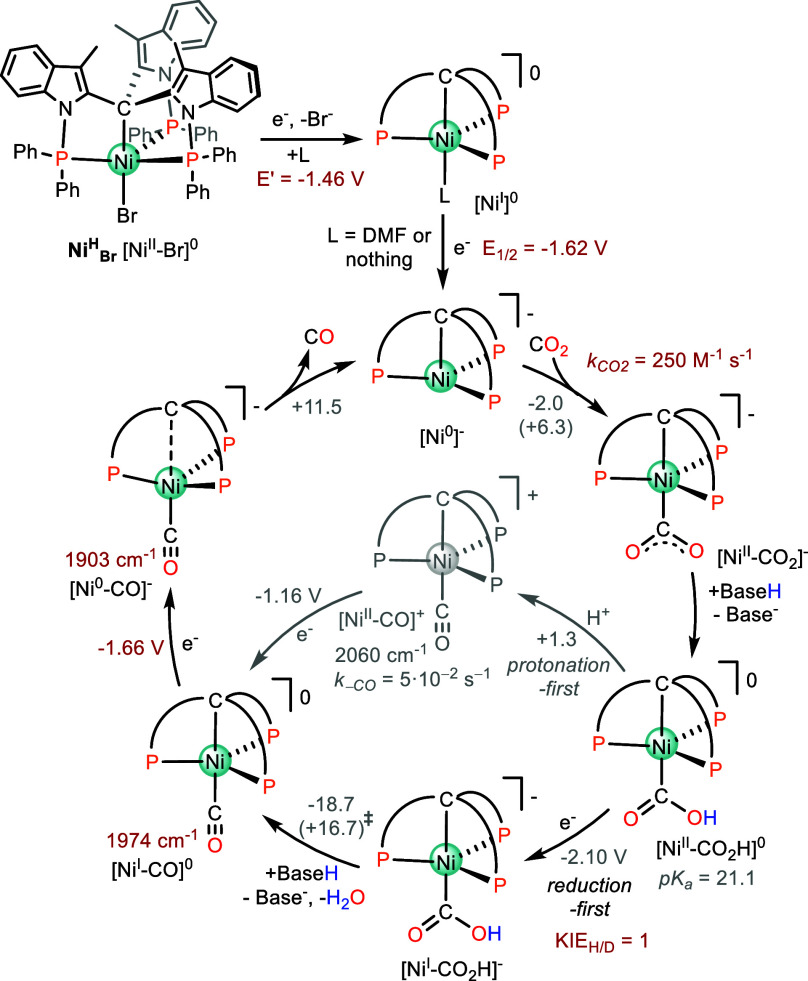
Mechanistic Overview for the Electrocatalytic CO_2_ Reduction to CO with the Catalyst **Ni** ^**H**^_**Br**_

From CV titration experiments, we have determined a first-order dependence of the catalytic current on the water concentration reaching its maximum at 3.6 M H_2_O. Using D_2_O as the added proton source excluded an isotopic effect in the kinetics of the CO_2_ reduction process. These results are in line with a proton-assisted reaction where the rate-determining step does not involve the protonation of an intermediate. The modeled Gibbs energy profile provides a reasonable explanation for the latter experimental observations: water is essential for the reaction to occur, but at high water concentration (≥3.5 M), the energy barrier is only dependent on the CO_2_ binding and CO release steps. Among the two, CO release emerges as the main contributor to the kinetic barrier, as we have previously observed in other CO_2_ reduction catalysts based first–row transition metals.^24,25^

## Conclusions

For the first time, we report the CO_2_ reduction electrocatalytic activity of an organometallic Ni complex (**Ni** ^**H**^_**Br**_) supported by a tris­(phosphino)­alkyl ligand. This complex shows a trigonal-bipyramidal geometry with a metalated alkyl ligand trans to the CO_2_ binding site. **Ni** ^**H**^_**Br**_ shows an excellent catalytic performance under preparative CPE conditions, providing >90% FY for CO production at −2.3 V in DMF/[TBA]­PF_6_ (0.1 M) with 3.5 M of added H_2_O. Under these conditions, **Ni** ^**H**^_**Br**_ shows a canonical *S-*shaped catalytic wave that allowed for the determination of a TO*F*_max_ = 64 s^–1^. Moreover, the catalytic process starts to occur at 350 mV more negative than the Ni^0/–^ process, which is in line with one electron reduction after the formation of the [Ni–CO_2_H]^0^ intermediate. Titration experiments with H_2_O and D_2_O revealed a KIE_H/D_ of 1, which is in line with a proton-assisted mechanism in which the protonation events are not rate-determining. Starting from **Ni** ^**H**^_**MeCN**_, we could synthesize and characterize the corresponding [Ni^II^–CO]^+^ complex (**Ni** ^**H**^_**CO**_). FTIR-SEC experiments allowed for the detection of three different CO stretching signals at 2060, 1974, and 1903 cm^–1^ corresponding to [Ni–CO]^+^, [Ni–CO]^0^, and [Ni–CO]^−^, respectively.

The DFT calculated Gibbs energy profile shows that the proton-assisted reduction first mechanism can operate at room temperature. At −2.1 V, the rate of the reaction is limited by the CO_2_ binding and CO release steps, which is in line with the absence of KIE_H/D_. This highly chelating tris­(phosphino)­alkyl ligand imposes unique geometric and electronic features that are beneficial for the CO_2_ reduction to CO. We envision that this new ligand platform can serve as a model to understand how the trans effect of a strongly σ-donating alkyl ligand impacts the coordination and reactivity of CO as an intermediate in the multielectron cascade reduction of CO_2_.

## Experimental Section

All manipulations were carried out under an inert atmosphere (N_2_, Ar) using glovebox and Schlenk techniques or under CO_2_. Anhydrous solvents (MeCN, DMF) and TBAPF_6_ as well as other commercial reagents were purchased and stored under N_2_. **Ni** ^**H**^_**Br**_ was synthesized following our reported procedure and converted to **Ni** ^**H**^_**MeCN**_ (Section S2 of the Supporting Information). NMR spectra were collected at 298 K on a Bruker AV400 spectrometer. Single-crystal X-ray diffraction data were obtained at 100 K on a Bruker Kappa Apex II DUO with Mo Kα radiation. Electrochemical experiments employed Bio-Logic VSP-50 or CHI700E potentiostats. CVs were recorded in Ar- or CO_2_-sparged 0.1 M TBAPF_6_/MeCN using glassy carbon working electrodes, a Pt counter electrode, and an Ag pseudoreference; voltammograms are represented following the IUPAC convention with potentials referenced to Fc^+/0^. CPE experiments were performed in an H-cell (3 mL catholyte) with glassy carbon rod working electrodes under stirring. Gas products were quantified by GC-TCD (CP-CarboPlot P7). FTIR spectroelectrochemical measurements were conducted in an OTTLE cell (Pt minigrid electrode, CaF_2_ window) at 4 mM catalyst concentration in 0.2 M TBAPF_6_/MeCN. DFT calculations have been performed with the *Gaussian09* and *Gaussian16* software packages. A more detailed explanation of the experimental and computational details can be found in the Supporting Information.

## Supplementary Material







## Notes

Experimental section with materials and instrumentation, synthetic, characterization, electrochemical, spectroelectrochemical, computational methods and geometric coordinates (PDF)
Crystallographic information for [NiHMeCN]­BF4 and [NiHCO]­BF_4_ (CIF)
Crystallographic information for [NiHCO]­BF_4_ (CIF)
