# Supplementary material for: Selective CO2 Electroreduction to CO by an Organometallic Nickel Catalyst Featuring a C3–Symmetric Tris(Phosphino)Alkyl Ligand
Source: ACS Catal. 2026 Feb 4;16(4):3831–40. doi: 10.1021/acscatal.5c08299 (PMC12930349; doi:10.1021/acscatal.5c08299)
Supplement: Supplementary file 1 [file cs5c08299_si_001.pdf]

Supplementary material for:

Selective CO_2 Electroreduction to CO by an Organometallic Nickel Catalyst Featuring a C_3-Symmetric Tris(phosphino)alkyl Ligand

Sergio Fernández,[†] Klaudia Michaliszyn,[†] Ekaterina S. Smirnova,[†] Marc Robert,[§] Josep M. Luis,[‡] Julio Lloret-Fillol[†,‖]

[†] Institute of Chemical Research of Catalonia (ICIQ), The Barcelona Institute of Science and Technology, Avinguda Països Catalans 16, 43007 Tarragona, Spain.

[§] Sorbonne Université, CNRS, Institut Parisien de Chimie Moléculaire, IPCM, F-75005 Paris, France.

[‡] Institut de Química Computacional i Catàlisi (IQCC) and Departament de Química, Universitat de Girona, Campus Montilivi, Girona, E-17003 Catalonia, Spain.

[‖] Catalan Institution for Research and Advanced Studies (ICREA), Passeig Lluïs Companys, 23, 08010 Barcelona, Spain.

1. Materials and methods

All the procedures and manipulations were carried out under inert (N_2 or Ar) using standard glovebox and Schlenk techniques or under CO_2. The quality of the employed gases (N_2, Ar or CO_2) was 99.9995 % (5.5). Reagents and solvents were purchased from commercial sources and used as received unless otherwise stated. Anhydrous CH_3CN and DMF (≥99.8%) as well as Tetrabutylammonium hexafluorophosphate (≥99.0%, for electrochemical analysis) were purchased from Sigma-Aldrich. Complex $\mathbf{Ni^H_{Br}}$ was synthesized following the procedure previously reported by our group[1] and used as precursor for the synthesis of $\mathbf{Ni^H_{MeCN}}$ (Section 2).

NMR spectra were recorded on Bruker AV400 spectrometers using standard conditions (298 K) unless otherwise indicated. All ^1H chemical shifts are reported in ppm and have been internally calibrated to the residual protons of the deuterated solvent.

Full sphere **single crystal data** collection was performed at 100 K on a Bruker Kappa Apex II DUO diffractometer equiped with a Cryostream 700 plus low temperature device, a microsource anode with Mo Kα (λ= 0.71073 Å).

All the **electrochemical experiments** were carried out with a VSP-50 potentiostat from Bio-Logic, equipped with the EC-Lab software or with a CHI700E Bipotentiostat.

For **Cyclic Voltammetry** (CV) measurements, a double-wall jacketed single-compartment cell was used. It was filled with Ar or CO_2-sparged $TBAPF_6$/MeCN (0.1 M) electrolyte solutions of the complex under study. A freshly polished 3 mm diameter glassy carbon disks was used as working electrode, a Pt wire as a counter electrode and a Ag wire as pseudo-reference, immerged in a bridge tube containing the same electrolyte solution (0.1 M, $TBAPF_6$/MeCN) and separated from the working solution by a porous tip. Ferrocene (Fc) was used as an internal or external standard and all the potentials are referenced *vs.* the $Fc^{+/0}$ redox couple. The Ohmic drop has been corrected ≥85% for all CV experiments using either a EIS-based method implemented in EC-Lab (ZIR) or by a positive feedback compensation implemented in the CHI Bipotentiostat.

For **Controlled-Potential electrolysis** (CPE), the cathodic compartment of an H-type electrochemical cell was filled with 3 ml of $TBAPF_6$/MeCN (0.1 M) solution of the catalyst, is maintained under constant stirring in the course of the experiment. A glassy carbon rod (1.8 cm^2) was used as a working electrode, and an Ag wire as a pseudo-reference, immerged in a bridge tube filled with the same electrolyte solution and separated from the working solution by a porous tip. The redox potential of the $Fc^{+/0}$ couple was checked in a separate cell prior to and after electrolysis. The counter electrode was a Pt wire immerged in a bridge tube containing the electrolyte solution and separated from the cathodic compartment by a ceramic frit. Solutions were magnetically stirred during the electrolysis.

Gas products were analyzed during electrolysis experiments by manual injection in a calibrated 7820A GC-TCD from Agilent Technologies equipped with a CP-CarboPlot P7 capillary column (27.46 m in length and 25 µm internal diameter) using argon as a carrier gas.

FTIR Spectroelectrochemistry experiments were performed in a optically transparent thin-layer electrode (OTTLE) cell equipped with a Pt minigrid working and auxiliary electrodes, an Ag microwire pseudo-reference electrode and a CaF_2 window. For electrochemical measurements we have used used an SP-50 potentiostat from Bio-Logic and FTIR spectra were recorded on a Nicolet iS50 FT-IR spectrometer equipped with an MCT detector. All the experiments were

carried out under an inert (Ar or N_2) atmosphere at 4 mM solution of complex in $TBAPF_6$/MeCN (0.2 M).

2. Synthesis and characterization of complexes

Synthesis of $\mathbf{Ni^H_{MeCN}}$. Under N_2 atmosphere, a suspension of $AgBF_4$ (15 mg, 0.077 mmol) in MeCN was added dropwise to a solution of $\mathbf{Ni^H_{Br}}$ (82 mg, 0.075 mmol) in CH_2Cl_2 (2 ml). The resulting suspension was stirred for 3 hours and filtered over Celite®. The resulting liquid phase was evaporated under reduced pressure and ($\mathbf{Ni^H_{MeCN}}$)BF_4 was obtained as a shiny black solid (74 mg, 0.064 mmol) in 86% yield. Suitable crystals for X-ray diffraction were obtained by slow diffusion of hexane into a solution of ($\mathbf{Ni^H_{MeCN}}$)BF_4 in $CDCl_3$. ^1H NMR (400 MHz, $CDCl_3$) δ 7.41–7.32 (m, 9H), 7.26–7.05 (m, 27H), 6.95 (t, 3H), 6.65 (d, 3H), 1.35 (s, 9H), 1.25 (s, 3H). $^{31}P\{^1H\}$ NMR (162 MHz, $CDCl_3$) δ 60.10 (s).

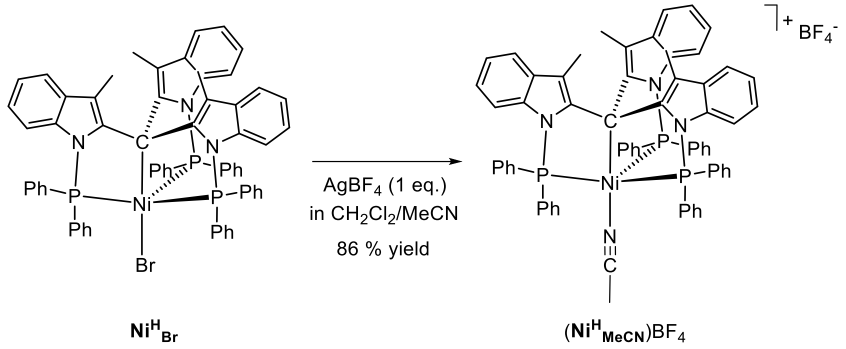

Scheme S 1. Synthesis of $\mathbf{Ni^H_{MeCN}}$ from $\mathbf{Ni^H_{Br}}$.

Synthesis of $\mathbf{Ni^H_{CO}}$. A solution of ($\mathbf{Ni^H_{MeCN}}$)BF_4 (11 mg, 0.009 mmol) in CH_2Cl_2 (2 ml) was stirred overnight under CO atmosphere (1 atm). A dark red powder (8.7 mg, 0.008 mmol, 84% yield) was obtained after precipitation with hexane followed by cannula filtration and after drying the solid under reduced pressure. Suitable crystals for X-ray diffraction were obtained by slow diffusion of pentane into a solution of ($\mathbf{Ni^H_{MeCN}}$)BF_4 in $CDCl_3$. ^1H NMR (400 MHz, $CDCl_3$) δ 7.44–7.42 (m, 9H), 7.26–7.13 (m, 21H), 6.96-6.92 (m, 9H), 6.46 (d, 3H), 1.56 (s, 9H). $^{31}P\{^1H\}$ NMR (162 MHz, $CDCl_3$) δ 69.51 (s).

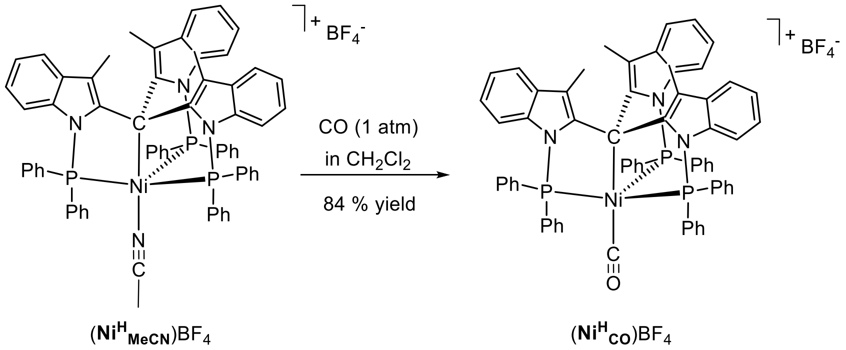

Scheme S 2. Synthesis of $\mathbf{Ni^H_{CO}}$ from $\mathbf{Ni^H_{MeCN}}$.

2.1. $^{31}P\{^1H\}$-NMR in $CDCl_3$

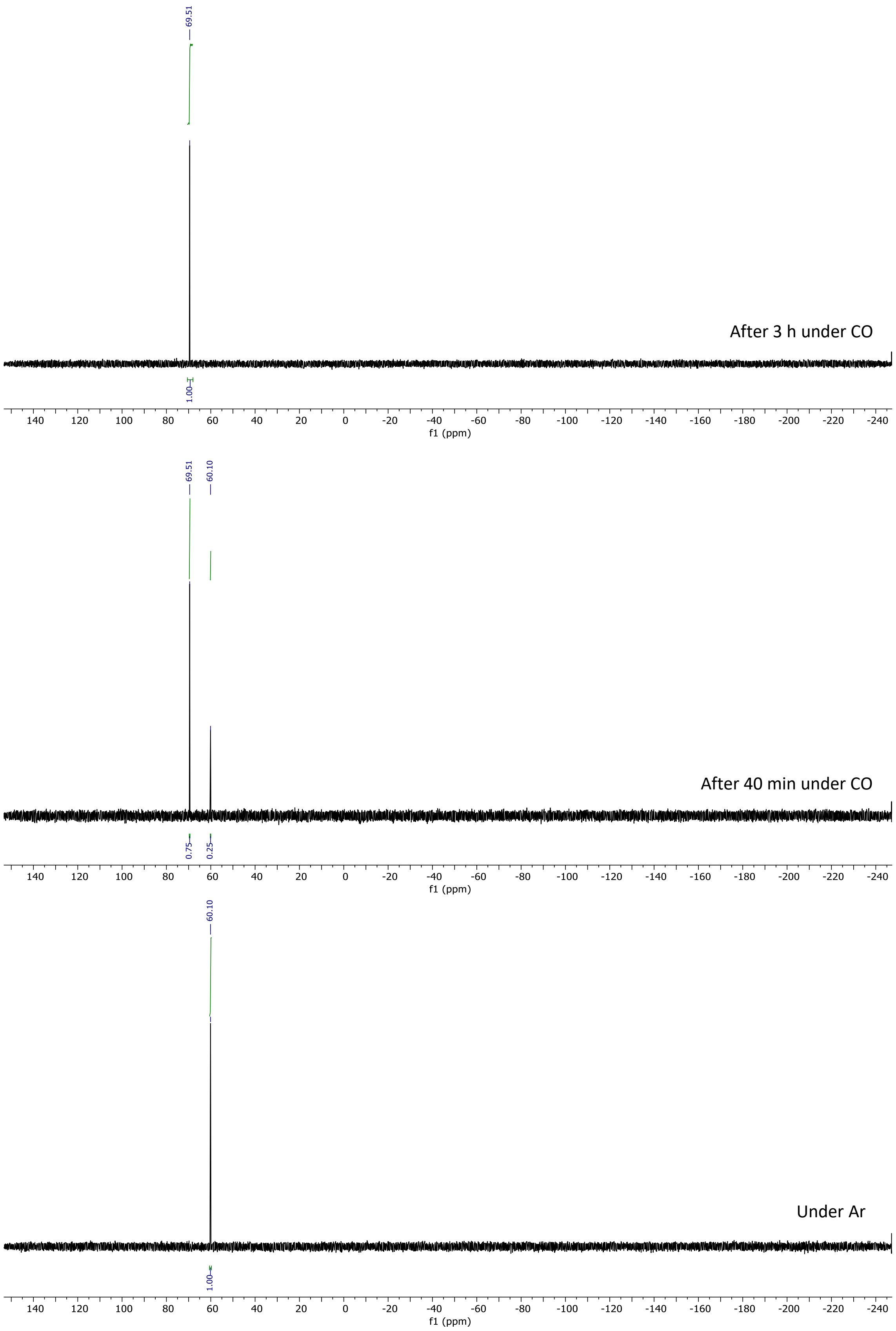

Figure S 1. $^{31}P\{^1H\}$-NMR (162 MHz, $CDCl_3$) of $\mathbf{Ni^H_{MeCN}}$ (bottom), $\mathbf{Ni^H_{CO}}$ (top) and the reaction mixture before completion (middle) [Ni-MeCN]BF_4 under Ar and CO.

2.2. ^{1}H-NMR in $CDCl_3$

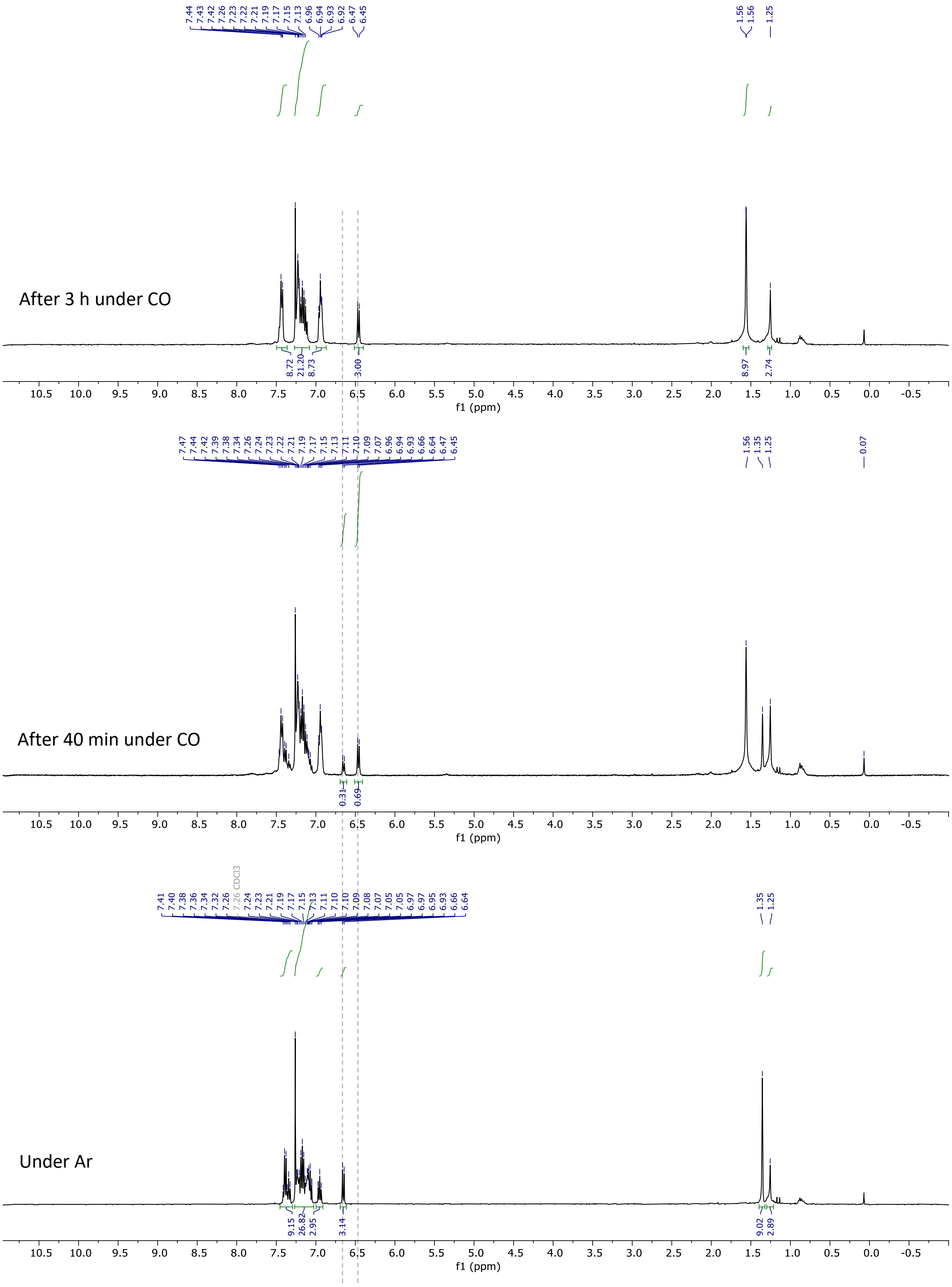

Figure S 2. ^{1}H-NMR (400 MHz, $CDCl_3$) of **Ni^{H}_{MeCN}** (bottom), **Ni^{H}_{CO}** (top) and the reaction mixture before completion (middle) [Ni-MeCN]BF_4 under Ar and CO.

3. Single crystal X-ray diffraction

3.1. Crystal structure of (Ni$^{H}_{MeCN}$)BF$_4$

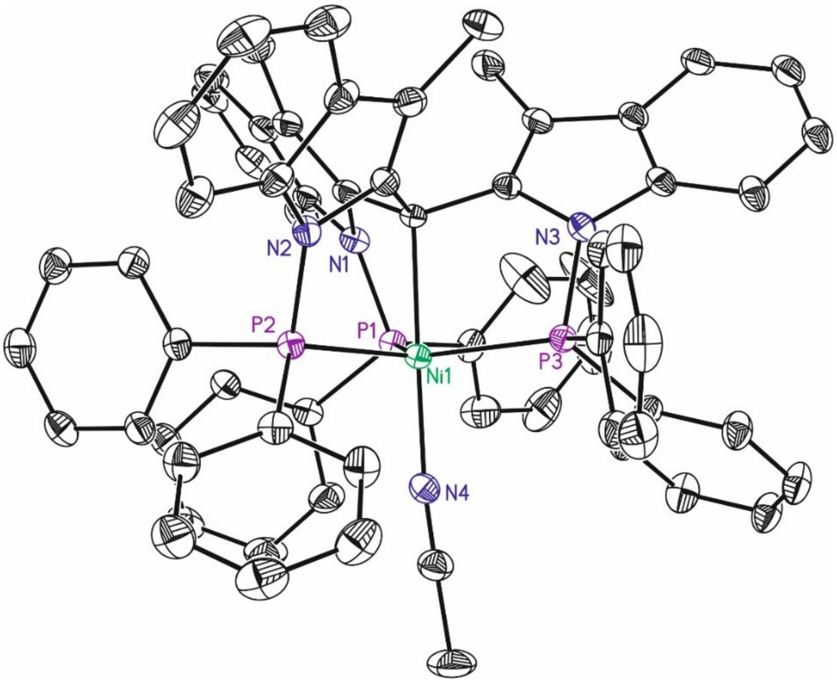

Figure S 3. X-ray structure of (**Ni$^{H}_{MeCN}$**)BF$_4$ with thermal ellipsoids at 50% probability. Non relevant hydrogen atoms, anions and solvent molecules have been ommited for clarity.

Table S 1. Crystal data and structure refinement for (**Ni$^{H}_{MeCN}$**)BF$_4$.

Identification code	mo_SFL30861_0m	
Empirical formula	C69 H57 B Cl9 F4 N4 Ni P3	
Formula weight	1499.66	
Temperature	100(2)K	
Wavelength	0.71073 Å	
Crystal system	triclinic	
Space group	P -1	
Unit cell dimensions	a = 12.6092(7)Å	a= 83.3883(16)°.
	b = 15.0493(9)Å	b = 72.8276(14)°.
	c = 19.7946(11)Å	g = 78.1182(17)°.
Volume	3505.9(3) Å3	
Z	2	
Density (calculated)	1.421 Mg/m3	
Absorption coefficient	0.743 mm-1	
F(000)	1532	
Crystal size	0.150 x 0.100 x 0.050 mm3	
Theta range for data collection	1.705 to 30.147°.	
Index ranges	-17<=h<=10,-21<=k<=15,-27<=l<=27	
Reflections collected	39711	
Independent reflections	19603[R(int) = 0.0372]	
Completeness to theta =30.147°	94.5%	
Absorption correction	Multi-scan	
Max. and min. transmission	0.74 and 0.61	
Refinement method	Full-matrix least-squares on F2	
Data / restraints / parameters	19603/ 126/ 968	
Goodness-of-fit on F2	1.039	
Final R indices [I>2sigma(I)]	R1 = 0.0618, wR2 = 0.1642	

R indices (all data)	R1 = 0.1029, wR2 = 0.1907
Largest diff. peak and hole	1.538 and -1.109 e.Å-3

Bond lengths----

Ni1	N4	1.928(2)
Ni1	C1	2.054(3)
Ni1	P2	2.2378(8)
Ni1	P3	2.2485(8)
Ni1	P1	2.2640(8)
P1	N1	1.706(2)
P1	C11	1.820(3)
P1	C17	1.832(7)
P1	C17'	1.832(8)
P2	N2	1.714(3)
P2	C38	1.816(3)
P2	C32	1.814(3)
P3	N3	1.711(2)
P3	C59	1.811(3)
P3	C53	1.831(3)
C3	C2	1.371(4)
C3	C4	1.435(4)
C3	C10	1.500(4)
N1	C9	1.395(4)
N1	C2	1.403(4)
N2	C30	1.397(4)
N2	C23	1.417(3)
N3	C51	1.398(3)
N3	C44	1.413(4)
N4	C67	1.134(4)
C1	C23	1.494(4)
C1	C44	1.499(4)
C1	C2	1.498(4)
C4	C9	1.411(4)
C4	C5	1.407(4)
C5	C6	1.387(5)
C6	C7	1.397(5)
C7	C8	1.387(5)
C8	C9	1.388(5)
C11	C12	1.386(4)
C11	C16	1.398(4)
C12	C13	1.386(4)
C13	C14	1.375(5)
C14	C15	1.386(5)
C15	C16	1.389(5)
C17	C18	1.3900
C17	C22	1.3900
C18	C19	1.3900
C19	C20	1.3900
C20	C21	1.3900
C21	C22	1.3900
C17'	C18'	1.387(4)
C17'	C22'	1.387(4)
C18'	C19'	1.390(4)
C19'	C20'	1.385(5)
C20'	C21'	1.384(5)
C21'	C22'	1.392(4)
C23	C24	1.367(4)
C24	C25	1.444(4)
C24	C31	1.493(4)
C26	C27	1.378(5)
C26	C25	1.398(4)
C25	C30	1.413(4)
C27	C28	1.403(5)
C29	C28	1.385(5)
C29	C30	1.392(4)
C53	C54	1.385(4)
C53	C58	1.396(4)
C32	C37	1.389(4)
C32	C33	1.400(4)
C33	C34	1.378(4)
C34	C35	1.396(5)
C35	C36	1.383(5)
C38	C39	1.392(4)
C38	C43	1.404(4)
C56	C57	1.380(5)
C56	C55	1.381(4)
C37	C36	1.391(4)
C39	C40	1.389(5)
C40	C41	1.386(5)
C44	C45	1.368(4)
C41	C42	1.378(5)
C43	C42	1.385(5)
C48	C49	1.392(5)
C48	C47	1.392(5)
C47	C46	1.408(4)
C49	C50	1.391(4)
C46	C51	1.407(4)
C46	C45	1.440(4)
C45	C52	1.491(4)
C50	C51	1.392(4)
C55	C54	1.388(4)
C57	C58	1.396(4)
C59	C64	1.398(4)
C59	C60	1.398(4)
C63	C62	1.386(5)
C63	C64	1.389(4)
C62	C61	1.378(5)
C61	C60	1.394(5)
C67	C68	1.460(4)
F1B	B1B	1.374(5)
F2B	B1B	1.394(5)
F3B	B1B	1.388(6)
F4B	B1B	1.366(5)
Cl1R	C1R	1.769(10)
Cl1R	Cl2R	2.382(5)
Cl2R	C1R	1.779(10)
Cl3R	C1R	1.774(9)
C1R'	Cl5R	1.690(16)
C1R'	Cl6R	1.757(17)
C1R'	Cl4R	1.821(14)
Cl1S	C1S	1.771(4)
Cl2S	C1S	1.742(4)
Cl3S	C1S	1.749(4)
C1T	Cl2T	1.679(7)
C1T	Cl1T	1.721(6)
C1T	Cl3T	1.736(6)
C1'	Cl2'	1.674(8)
C1'	Cl1'	1.712(8)
C1'	Cl3'	1.729(8)
C1U	Cl3U	1.766(8)
C1U	Cl2U	1.770(9)
C1U	Cl1U	1.770(9)

Angles----------

N4	Ni1	C1	178.69(11)
N4	Ni1	P2	96.26(7)
C1	Ni1	P2	84.02(8)
N4	Ni1	P3	93.83(8)
C1	Ni1	P3	84.93(8)
P2	Ni1	P3	120.63(3)
N4	Ni1	P1	96.06(8)
C1	Ni1	P1	84.95(8)
P2	Ni1	P1	116.11(3)
P3	Ni1	P1	120.67(3)
N1	P1	C11	107.20(13)
N1	P1	C17	103.9(3)
C11	P1	C17	104.2(3)
N1	P1	C17'	101.3(4)
C11	P1	C17'	105.8(3)
N1	P1	Ni1	99.29(9)
C11	P1	Ni1	113.11(10)
C17	P1	Ni1	127.2(3)
C17'	P1	Ni1	127.5(3)
N2	P2	C38	107.19(13)
N2	P2	C32	105.70(13)
C38	P2	C32	103.77(13)
N2	P2	Ni1	99.79(8)
C38	P2	Ni1	118.68(10)
C32	P2	Ni1	120.32(9)
N3	P3	C59	104.17(13)
N3	P3	C53	107.12(12)
C59	P3	C53	104.64(13)
N3	P3	Ni1	99.47(8)
C59	P3	Ni1	124.16(9)
C53	P3	Ni1	115.45(10)
C2	C3	C4	106.2(3)
C2	C3	C10	129.5(3)
C4	C3	C10	124.2(3)
C9	N1	C2	107.8(2)
C9	N1	P1	132.6(2)
C2	N1	P1	116.72(19)
C30	N2	C23	107.7(2)
C30	N2	P2	133.8(2)
C23	N2	P2	115.68(18)
C51	N3	C44	107.7(2)
C51	N3	P3	132.0(2)
C44	N3	P3	115.84(18)
C67	N4	Ni1	174.7(3)
C23	C1	C44	112.2(2)
C23	C1	C2	112.6(2)
C44	C1	C2	111.5(2)
C23	C1	Ni1	106.91(18)
C44	C1	Ni1	106.08(17)
C2	C1	Ni1	107.06(18)
C3	C2	N1	110.2(3)
C3	C2	C1	133.4(3)
N1	C2	C1	115.7(2)
C9	C4	C5	118.9(3)
C9	C4	C3	108.3(3)
C5	C4	C3	132.7(3)
C6	C5	C4	118.6(3)
C5	C6	C7	121.1(3)
C8	C7	C6	121.5(3)

C7	C8	C9	117.3(3)
C8	C9	N1	130.2(3)
C8	C9	C4	122.5(3)
N1	C9	C4	107.2(3)
C12	C11	C16	118.6(3)
C12	C11	P1	123.5(2)
C16	C11	P1	117.7(2)
C13	C12	C11	120.4(3)
C14	C13	C12	120.6(3)
C13	C14	C15	119.9(3)
C14	C15	C16	119.6(3)
C15	C16	C11	120.8(3)
C18	C17	C22	120.0
C18	C17	P1	124.3(5)
C22	C17	P1	115.5(5)
C19	C18	C17	120.0
C18	C19	C20	120.0
C21	C20	C19	120.0
C20	C21	C22	120.0
C21	C22	C17	120.0
C18'	C17'	C22'	118.5(4)
C18'	C17'	P1	124.4(6)
C22'	C17'	P1	117.1(6)
C17'	C18'	C19'	120.6(5)
C20'	C19'	C18'	120.5(5)
C21'	C20'	C19'	119.3(5)
C20'	C21'	C22'	120.0(5)
C17'	C22'	C21'	121.0(5)
C24	C23	N2	110.3(2)
C24	C23	C1	133.8(3)
N2	C23	C1	115.5(2)
C23	C24	C25	106.3(3)
C23	C24	C31	130.4(3)
C25	C24	C31	123.0(3)
C27	C26	C25	118.3(3)
C26	C25	C30	119.6(3)
C26	C25	C24	132.1(3)
C30	C25	C24	108.3(3)
C26	C27	C28	121.5(3)
C28	C29	C30	117.2(3)
C54	C53	C58	119.0(3)
C54	C53	P3	118.0(2)
C58	C53	P3	123.0(2)
C29	C30	N2	130.6(3)
C29	C30	C25	122.1(3)
N2	C30	C25	107.4(3)
C37	C32	C33	119.3(3)
C37	C32	P2	122.0(2)
C33	C32	P2	118.4(2)
C34	C33	C32	120.1(3)
C33	C34	C35	120.3(3)
C36	C35	C34	119.7(3)
C39	C38	C43	118.5(3)
C39	C38	P2	117.4(2)
C43	C38	P2	124.0(2)
C57	C56	C55	120.0(3)
C32	C37	C36	120.3(3)
C38	C39	C40	121.0(3)
C41	C40	C39	119.8(3)
C45	C44	N3	110.0(2)
C45	C44	C1	133.9(3)
N3	C44	C1	115.7(2)
C42	C41	C40	119.9(3)
C42	C43	C38	120.0(3)
C49	C48	C47	121.2(3)
C48	C47	C46	118.6(3)
C43	C42	C41	120.8(3)
C48	C49	C50	121.4(3)
C51	C46	C47	119.0(3)
C51	C46	C45	108.2(2)
C47	C46	C45	132.8(3)
C44	C45	C46	106.5(3)
C44	C45	C52	129.5(3)
C46	C45	C52	123.9(3)
C49	C50	C51	117.3(3)
C50	C51	N3	129.9(3)
C50	C51	C46	122.5(3)
N3	C51	C46	107.5(2)
C29	C28	C27	121.3(3)
C56	C55	C54	120.2(3)
C56	C57	C58	120.0(3)
C35	C36	C37	120.2(3)
C53	C54	C55	120.6(3)
C53	C58	C57	120.2(3)
C64	C59	C60	119.2(3)
C64	C59	P3	120.9(2)
C60	C59	P3	119.5(2)
C62	C63	C64	120.2(3)
C61	C62	C63	120.1(3)
C62	C61	C60	120.3(3)
C63	C64	C59	120.2(3)
N4	C67	C68	177.9(4)
C59	C60	C61	120.0(3)
F4B	B1B	F1B	110.0(4)
F4B	B1B	F3B	110.1(3)
F1B	B1B	F3B	108.4(4)
F4B	B1B	F2B	109.7(3)
F1B	B1B	F2B	109.0(3)
F3B	B1B	F2B	109.6(4)
C1R	Cl1R	Cl2R	48.0(3)
C1R	Cl2R	Cl1R	47.6(3)
Cl1R	C1R	Cl3R	109.2(7)
Cl1R	C1R	Cl2R	84.3(5)
Cl3R	C1R	Cl2R	112.5(7)
Cl5R	C1R'	Cl6R	112.7(9)
Cl5R	C1R'	Cl4R	135.7(9)
Cl6R	C1R'	Cl4R	100.1(9)
Cl2S	C1S	Cl3S	111.6(2)
Cl2S	C1S	Cl1S	110.6(2)
Cl3S	C1S	Cl1S	109.6(2)
Cl2T	C1T	Cl1T	110.8(4)
Cl2T	C1T	Cl3T	115.7(5)
Cl1T	C1T	Cl3T	114.0(4)
Cl2'	C1'	Cl1'	113.1(8)
Cl2'	C1'	Cl3'	114.4(8)
Cl1'	C1'	Cl3'	117.6(8)
Cl3U	C1U	Cl2U	110.4(6)
Cl3U	C1U	Cl1U	110.0(6)
Cl2U	C1U	Cl1U	110.4(6)

Torsion angles [°]

C11	P1	N1	C9	-41.3(3)
C17	P1	N1	C9	68.6(4)
C17'	P1	N1	C9	69.3(4)
Ni1	P1	N1	C9	-159.2(3)
C11	P1	N1	C2	117.0(2)
C17	P1	N1	C2	-133.1(4)
C17'	P1	N1	C2	-132.4(4)
Ni1	P1	N1	C2	-0.9(2)
C38	P2	N2	C30	-27.6(3)
C32	P2	N2	C30	82.6(3)
Ni1	P2	N2	C30	-151.9(3)
C38	P2	N2	C23	130.6(2)
C32	P2	N2	C23	-119.2(2)
Ni1	P2	N2	C23	6.3(2)
C59	P3	N3	C51	79.5(3)
C53	P3	N3	C51	-31.0(3)
Ni1	P3	N3	C51	-151.5(3)
C59	P3	N3	C44	-127.7(2)
C53	P3	N3	C44	121.8(2)
Ni1	P3	N3	C44	1.3(2)
C4	C3	C2	N1	-3.0(3)
C10	C3	C2	N1	-178.2(3)
C4	C3	C2	C1	166.6(3)
C10	C3	C2	C1	-8.6(5)
C9	N1	C2	C3	4.8(3)
P1	N1	C2	C3	-158.6(2)
C9	N1	C2	C1	-166.8(2)
P1	N1	C2	C1	29.8(3)
C23	C1	C2	C3	28.6(4)
C44	C1	C2	C3	-98.6(4)
Ni1	C1	C2	C3	145.8(3)
C23	C1	C2	N1	-162.3(2)
C44	C1	C2	N1	70.5(3)
Ni1	C1	C2	N1	-45.1(3)
C2	C3	C4	C9	0.1(3)
C10	C3	C4	C9	175.6(3)
C2	C3	C4	C5	-176.8(3)
C10	C3	C4	C5	-1.3(5)
C9	C4	C5	C6	-2.3(4)
C3	C4	C5	C6	174.4(3)
C4	C5	C6	C7	0.8(5)
C5	C6	C7	C8	1.4(5)
C6	C7	C8	C9	-1.9(5)
C7	C8	C9	N1	-177.7(3)
C7	C8	C9	C4	0.4(4)
C2	N1	C9	C8	173.8(3)
P1	N1	C9	C8	-26.5(5)
C2	N1	C9	C4	-4.6(3)
P1	N1	C9	C4	155.1(2)
C5	C4	C9	C8	1.7(4)
C3	C4	C9	C8	-175.7(3)
C5	C4	C9	N1	-179.8(3)
C3	C4	C9	N1	2.8(3)
N1	P1	C11	C12	-11.1(3)
C17	P1	C11	C12	-120.8(4)
C17'	P1	C11	C12	-118.6(5)
Ni1	P1	C11	C12	97.3(2)
N1	P1	C11	C16	174.6(2)
C17	P1	C11	C16	64.8(4)
C17'	P1	C11	C16	67.0(5)
Ni1	P1	C11	C16	-77.0(3)
C16	C11	C12	C13	-2.5(5)
P1	C11	C12	C13	-176.8(2)
C11	C12	C13	C14	0.6(5)
C12	C13	C14	C15	1.0(6)
C13	C14	C15	C16	-0.8(6)
C14	C15	C16	C11	-1.2(6)

C12	C11	C16	C15	2.8(5)
P1	C11	C16	C15	177.5(3)
N1	P1	C17	C18	-140.6(5)
C11	P1	C17	C18	-28.5(6)
Ni1	P1	C17	C18	106.0(5)
N1	P1	C17	C22	45.2(5)
C11	P1	C17	C22	157.3(4)
Ni1	P1	C17	C22	-68.2(5)
C22	C17	C18	C19	0.0
P1	C17	C18	C19	-174.0(8)
C17	C18	C19	C20	0.0
C18	C19	C20	C21	0.0
C19	C20	C21	C22	0.0
C20	C21	C22	C17	0.0
C18	C17	C22	C21	0.0
P1	C17	C22	C21	174.5(8)
N1	P1	C17'	C18'	-109.5(11)
C11	P1	C17'	C18'	2.2(13)
Ni1	P1	C17'	C18'	139.3(10)
N1	P1	C17'	C22'	69.0(9)
C11	P1	C17'	C22'	-179.3(7)
Ni1	P1	C17'	C22'	-42.2(10)
C22'	C17'	C18'	C19'	0(2)
P1	C17'	C18'	C19'	178.3(10)
C17'	C18'	C19'	C20'	0(3)
C18'	C19'	C20'	C21'	0(3)
C19'	C20'	C21'	C22'	0(2)
C18'	C17'	C22'	C21'	0.8(17)
P1	C17'	C22'	C21'	-177.8(8)
C20'	C21'	C22'	C17'	-0.9(18)
C30	N2	C23	C24	1.6(3)
P2	N2	C23	C24	-162.1(2)
C30	N2	C23	C1	-171.9(2)
P2	N2	C23	C1	24.4(3)
C44	C1	C23	C24	27.4(4)
C2	C1	C23	C24	-99.4(4)
Ni1	C1	C23	C24	143.3(3)
C44	C1	C23	N2	-161.1(2)
C2	C1	C23	N2	72.1(3)
Ni1	C1	C23	N2	-45.2(3)
N2	C23	C24	C25	-1.3(3)
C1	C23	C24	C25	170.5(3)
N2	C23	C24	C31	-175.9(3)
C1	C23	C24	C31	-4.0(5)
C27	C26	C25	C30	0.0(5)
C27	C26	C25	C24	177.4(3)
C23	C24	C25	C26	-177.0(3)
C31	C24	C25	C26	-1.9(5)
C23	C24	C25	C30	0.6(3)
C31	C24	C25	C30	175.6(3)
C25	C26	C27	C28	0.4(5)
N3	P3	C53	C54	-90.0(2)
C59	P3	C53	C54	159.8(2)
Ni1	P3	C53	C54	19.7(3)
N3	P3	C53	C58	90.3(3)
C59	P3	C53	C58	-19.9(3)
Ni1	P3	C53	C58	-160.0(2)
C28	C29	C30	N2	-177.8(3)
C28	C29	C30	C25	0.9(5)
C23	N2	C30	C29	177.8(3)
P2	N2	C30	C29	-22.8(5)
C23	N2	C30	C25	-1.1(3)
P2	N2	C30	C25	158.3(2)
C26	C25	C30	C29	-0.7(4)
C24	C25	C30	C29	-178.7(3)
C26	C25	C30	N2	178.3(3)
C24	C25	C30	N2	0.3(3)
N2	P2	C32	C37	17.9(3)
C38	P2	C32	C37	130.6(2)
Ni1	P2	C32	C37	-93.7(2)
N2	P2	C32	C33	-168.2(2)
C38	P2	C32	C33	-55.6(3)
Ni1	P2	C32	C33	80.1(2)
C37	C32	C33	C34	-1.5(4)
P2	C32	C33	C34	-175.5(2)
C32	C33	C34	C35	1.6(5)
C33	C34	C35	C36	-0.3(5)
N2	P2	C38	C39	-85.6(3)
C32	P2	C38	C39	162.8(2)
Ni1	P2	C38	C39	26.2(3)
N2	P2	C38	C43	98.2(3)
C32	P2	C38	C43	-13.4(3)
Ni1	P2	C38	C43	-150.0(2)
C33	C32	C37	C36	0.1(4)
P2	C32	C37	C36	173.8(2)
C43	C38	C39	C40	-0.4(5)
P2	C38	C39	C40	-176.8(3)
C38	C39	C40	C41	0.8(6)
C51	N3	C44	C45	1.9(3)
P3	N3	C44	C45	-157.2(2)
C51	N3	C44	C1	-171.6(2)
P3	N3	C44	C1	29.3(3)
C23	C1	C44	C45	-101.7(4)
C2	C1	C44	C45	25.6(4)
Ni1	C1	C44	C45	141.9(3)
C23	C1	C44	N3	69.8(3)
C2	C1	C44	N3	-162.8(2)
Ni1	C1	C44	N3	-46.6(3)
C39	C40	C41	C42	-1.1(6)
C39	C38	C43	C42	0.2(5)
P2	C38	C43	C42	176.4(3)
C49	C48	C47	C46	1.2(5)
C38	C43	C42	C41	-0.6(5)
C40	C41	C42	C43	1.0(6)
C47	C48	C49	C50	-0.6(5)
C48	C47	C46	C51	-0.9(4)
C48	C47	C46	C45	177.0(3)
N3	C44	C45	C46	-1.8(3)
C1	C44	C45	C46	170.1(3)
N3	C44	C45	C52	178.5(3)
C1	C44	C45	C52	-9.6(6)
C51	C46	C45	C44	1.0(3)
C47	C46	C45	C44	-177.0(3)
C51	C46	C45	C52	-179.3(3)
C47	C46	C45	C52	2.7(5)
C48	C49	C50	C51	-0.3(5)
C49	C50	C51	N3	-177.5(3)
C49	C50	C51	C46	0.6(4)
C44	N3	C51	C50	177.1(3)
P3	N3	C51	C50	-28.4(5)
C44	N3	C51	C46	-1.2(3)
P3	N3	C51	C46	153.2(2)
C47	C46	C51	C50	0.0(5)
C45	C46	C51	C50	-178.3(3)
C47	C46	C51	N3	178.5(3)
C45	C46	C51	N3	0.1(3)
C30	C29	C28	C27	-0.4(5)
C26	C27	C28	C29	-0.2(5)
C57	C56	C55	C54	0.2(6)
C55	C56	C57	C58	-0.4(6)
C34	C35	C36	C37	-1.2(5)
C32	C37	C36	C35	1.3(5)
C58	C53	C54	C55	-1.1(5)
P3	C53	C54	C55	179.1(3)
C56	C55	C54	C53	0.6(5)
C54	C53	C58	C57	1.0(5)
P3	C53	C58	C57	-179.3(3)
C56	C57	C58	C53	-0.2(5)
N3	P3	C59	C64	22.1(3)
C53	P3	C59	C64	134.4(2)
Ni1	P3	C59	C64	-90.0(2)
N3	P3	C59	C60	-165.2(2)
C53	P3	C59	C60	-52.9(3)
Ni1	P3	C59	C60	82.7(2)
C64	C63	C62	C61	-0.5(5)
C63	C62	C61	C60	0.1(5)
C62	C63	C64	C59	0.1(4)
C60	C59	C64	C63	0.7(4)
P3	C59	C64	C63	173.4(2)
C64	C59	C60	C61	-1.1(4)
P3	C59	C60	C61	-173.9(2)
C62	C61	C60	C59	0.7(5)
Cl2R	Cl1R	C1R	Cl3R	-111.8(8)
Cl1R	Cl2R	C1R	Cl3R	108.5(8)

3.2. X-ray structure of $(Ni^{H}_{CO})BF_4$

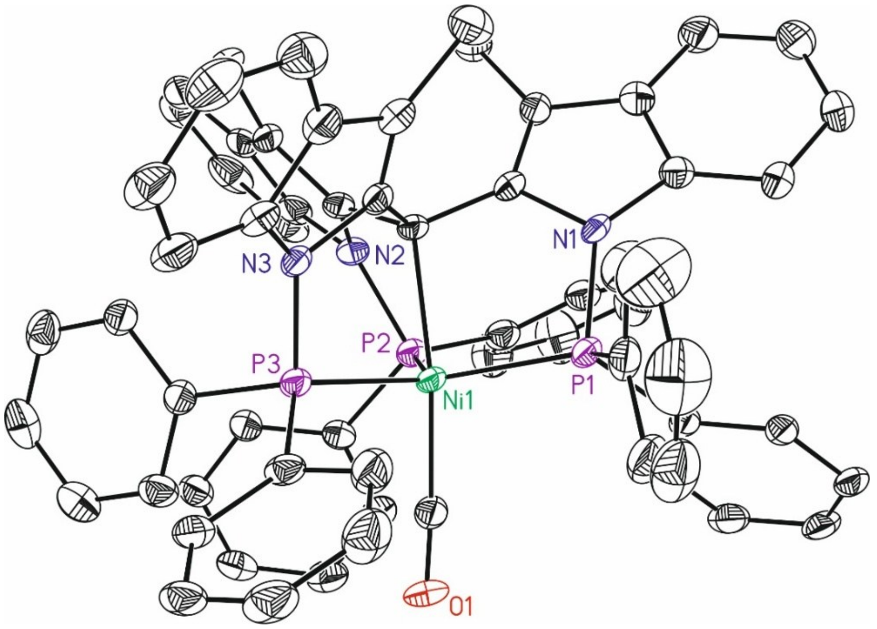

Figure S 4. X-ray structure of $(\mathbf{Ni^{H}_{CO}})BF_4$ with thermal ellipsoids at 50% probability. Non relevant hydrogen atoms, anions and solvent molecules have been omitted for clarity.

Table S 2. Crystal data and structure refinement for $(\mathbf{Ni^{H}_{CO}})BF_4$.

Identification code	sfl4006nico	
Empirical formula	C74.50 H72 B Cl4 F4 N3 Ni O P3	
Formula weight	1405.58	
Temperature	100(2)K	
Wavelength	0.71073 Å	
Crystal system	monoclinic	
Space group	P 21/n	
Unit cell dimensions	a = 10.13636(18)Å	a= 90°.
	b = 28.1668(5)Å	b = 95.0427(18)°.
	c = 23.8979(5)Å	g = 90°.
Volume	6796.7(2) $Å^3$	
Z	4	
Density (calculated)	1.374 Mg/m^3	
Absorption coefficient	0.572 mm^{-1}	
F(000)	2920	
Crystal size	0.150 x 0.100 x 0.100 mm^3	
Theta range for data collection	2.240 to 28.711°.	
Index ranges	-13<=h<=11,-37<=k<=37,-32<=l<=32	
Reflections collected	111808	
Independent reflections	15458[R(int) = 0.0805]	
Completeness to theta =28.711°	87.9%	
Absorption correction	Multi-scan	
Max. and min. transmission	1.00 and 0.47	
Refinement method	Full-matrix least-squares on F^2	
Data / restraints / parameters	15458/ 242/ 954	
Goodness-of-fit on F^2	1.148	
Final R indices [I>2sigma(I)]	R1 = 0.0946, wR2 = 0.2297	
R indices (all data)	R1 = 0.1347, wR2 = 0.2483	
Largest diff. peak and hole	1.604 and -1.118 e.$Å^{-3}$	

Bond lengths----

P1	N1	1.727(4)	
P1	C17	1.809(6)	
P1	C11	1.834(5)	
P1	Ni1	2.2497(14)	
O1	C65	1.122(6)	
C1	C2	1.486(7)	
C1	C44	1.491(7)	
C1	C23	1.502(7)	
C1	Ni1	2.092(4)	
N1	C9	1.411(6)	
N1	C2	1.421(6)	
Ni1	C65	1.822(5)	
Ni1	P3	2.2276(14)	
Ni1	P2	2.2678(14)	
P2	N2	1.705(4)	
P2	C32	1.815(5)	
P2	C38	1.833(5)	
N2	C30	1.404(6)	
N2	C23	1.415(7)	
C2	C3	1.368(7)	
P3	N3	1.714(4)	
P3	C59	1.801(5)	
P3	C53	1.820(5)	
N3	C51	1.391(7)	
N3	C44	1.416(6)	
C3	C4	1.423(7)	
C3	C10	1.512(7)	
C4	C5	1.392(7)	
C4	C9	1.416(7)	
C5	C6	1.372(9)	
C5	H5	0.9500	
C6	C7	1.395(9)	
C6	H6	0.9500	
C7	C8	1.395(8)	
C7	H7	0.9500	
C8	C9	1.380(7)	
C8	H8	0.9500	
C10	H10A	0.9800	
C10	H10B	0.9800	
C10	H10C	0.9800	
C11	C12	1.388(8)	
C11	C16	1.392(7)	
C12	C13	1.391(7)	
C12	H12	0.9500	
C13	C14	1.370(8)	
C13	H13	0.9500	
C14	C15	1.388(8)	
C14	H14	0.9500	
C15	C16	1.394(7)	
C15	H15	0.9500	
C16	H16	0.9500	
C17	C18	1.392(8)	
C17	C22	1.399(8)	
C18	C19	1.401(10)	
C18	H18	0.9500	
C19	C20	1.367(11)	
C19	H19	0.9500	
C20	C21	1.375(11)	
C20	H20	0.9500	
C21	C22	1.385(9)	
C21	H21	0.9500	
C22	H22	0.9500	
C23	C24	1.373(7)	
C24	C25	1.437(8)	
C24	C31	1.490(7)	
C25	C26	1.399(7)	
C25	C30	1.400(8)	
C26	C27	1.380(9)	
C26	H26	0.9500	
C27	C28	1.404(10)	
C27	H27	0.9500	
C28	C29	1.397(8)	
C28	H28	0.9500	
C29	C30	1.387(8)	
C29	H29	0.9500	
C31	H31A	0.9800	
C31	H31B	0.9800	
C31	H31C	0.9800	
C32	C37	1.388(7)	
C32	C33	1.399(7)	
C33	C34	1.381(8)	
C33	H33	0.9500	
C34	C35	1.385(8)	
C34	H34	0.9500	
C35	C36	1.386(8)	
C35	H35	0.9500	
C36	C37	1.392(7)	
C36	H36	0.9500	
C37	H37	0.9500	
C38	C39	1.384(8)	
C38	C43	1.390(8)	
C39	C40	1.379(8)	
C39	H39	0.9500	
C40	C41	1.383(9)	
C40	H40	0.9500	
C41	C42	1.369(10)	
C41	H41	0.9500	
C42	C43	1.390(9)	
C42	H42	0.9500	
C43	H43	0.9500	
C44	C45	1.363(7)	
C45	C46	1.445(8)	
C45	C52	1.514(7)	
C46	C47	1.378(8)	
C46	C51	1.419(7)	
C47	C48	1.381(8)	
C47	H47	0.9500	
C48	C49	1.411(9)	
C48	H48	0.9500	
C49	C50	1.385(8)	
C49	H49	0.9500	
C50	C51	1.389(8)	
C50	H50	0.9500	
C52	H52A	0.9800	
C52	H52B	0.9800	
C52	H52C	0.9800	
C53	C54	1.391(8)	
C53	C58	1.392(7)	
C54	C55	1.394(8)	
C54	H54	0.9500	
C55	C56	1.374(9)	
C55	H55	0.9500	
C56	C57	1.376(9)	
C56	H56	0.9500	
C57	C58	1.400(7)	
C57	H57	0.9500	
C58	H58	0.9500	
C59	C60	1.395(7)	
C59	C64	1.402(7)	
C60	C61	1.384(8)	
C60	H60	0.9500	
C61	C62	1.386(8)	
C61	H61	0.9500	
C62	C63	1.388(8)	
C62	H62	0.9500	
C63	C64	1.373(8)	
C63	H63	0.9500	
C64	H64	0.9500	
F1A	B1A	1.367(8)	
B1A	F2A	1.389(8)	
B1A	F4A	1.394(9)	
B1A	F3A	1.405(7)	
C1B	Cl2B	1.704(13)	
C1B	Cl1B	1.736(16)	
C1B	H1BA	0.9900	
C1B	H1BB	0.9900	
C1B'	Cl2'	1.790(5)	
C1B'	Cl1'	1.790(5)	
C1B'	H1BC	0.9900	
C1B'	H1BD	0.9900	
Cl1C	C1C	1.744(16)	
C1C	Cl2C	1.749(18)	
C1C	H1CA	0.9900	
C1C	H1CB	0.9900	
C1D	Cl1D	1.54(2)	
C1D	Cl2D	1.70(2)	
C1D	H1D	0.9500	
C1A	C2A	1.541(5)	
C1A	H1AA	0.9800	
C1A	H1AB	0.9800	
C1A	H1AC	0.9800	
C2A	C3A	1.537(5)	
C2A	H2AA	0.9900	
C2A	H2AB	0.9900	
C3A	C4A	1.539(5)	
C3A	H3AA	0.9900	
C3A	H3AB	0.9900	
C4A	C5A	1.538(5)	
C4A	H4AA	0.9900	
C4A	H4AB	0.9900	
C5A	C6A	1.535(5)	
C5A	H5AA	0.9900	
C5A	H5AB	0.9900	
C6A	H6AA	0.9800	
C6A	H6AB	0.9800	
C6A	H6AC	0.9800	
C1H	C2H	1.543(5)	
C1H	H1HA	0.9800	
C1H	H1HB	0.9800	
C1H	H1HC	0.9800	
C2H	C3H	1.539(5)	
C2H	H2HC	0.9900	
C2H	H2HD	0.9900	
C3H	C3H#	1.20(7)	3_766
C3H	H3HA	0.9900	
C3H	H3HB	0.9900	
C1H'	C2H'	1.542(5)	
C1H'	H1HD	0.9800	

C1H' H1HE 0.9800
C1H' H1HF 0.9800
C2H' C3H' 1.534(5)
C2H' H2HH 0.9900
C2H' H2HI 0.9900
C3H' C3H'# 1.56(5) 3_766
C3H' H3HE 0.9900
C3H' H3HD 0.9900
C1HH C2HH 1.35(5)
C1HH C1HH# 1.49(8) 3_666
C1HH H1AH 0.9900
C1HH H1BH 0.9900
C2HH C3HH 1.40(4)
C2HH H2HA 0.9900
C2HH H2HB 0.9900
C3HH H3AH 0.9800
C3HH H3BH 0.9800
C3HH H3HC 0.9800

Angles----------
N1 P1 C17 105.2(2)
N1 P1 C11 109.4(2)
C17 P1 C11 102.6(2)
N1 P1 Ni1 99.02(15)
C17 P1 Ni1 122.15(17)
C11 P1 Ni1 117.48(18)
C2 C1 C44 113.4(4)
C2 C1 C23 112.0(4)
C44 C1 C23 111.9(4)
C2 C1 Ni1 105.6(3)
C44 C1 Ni1 107.6(3)
C23 C1 Ni1 105.7(3)
C9 N1 C2 107.9(4)
C9 N1 P1 131.8(3)
C2 N1 P1 116.3(3)
C65 Ni1 C1 173.2(2)
C65 Ni1 P3 91.97(16)
C1 Ni1 P3 82.88(14)
C65 Ni1 P1 101.16(17)
C1 Ni1 P1 85.40(14)
P3 Ni1 P1 121.48(6)
C65 Ni1 P2 94.88(17)
C1 Ni1 P2 83.86(14)
P3 Ni1 P2 119.63(5)
P1 Ni1 P2 115.69(5)
N2 P2 C32 107.2(2)
N2 P2 C38 105.6(2)
C32 P2 C38 104.0(2)
N2 P2 Ni1 99.23(15)
C32 P2 Ni1 113.65(16)
C38 P2 Ni1 125.62(18)
C30 N2 C23 107.7(4)
C30 N2 P2 133.4(4)
C23 N2 P2 117.0(3)
C3 C2 N1 109.2(4)
C3 C2 C1 133.2(4)
N1 C2 C1 117.3(4)
N3 P3 C59 107.9(2)
N3 P3 C53 102.8(2)
C59 P3 C53 105.8(2)
N3 P3 Ni1 101.97(14)
C59 P3 Ni1 115.70(17)
C53 P3 Ni1 121.16(17)
C51 N3 C44 108.1(4)
C51 N3 P3 131.8(3)
C44 N3 P3 114.9(3)
C2 C3 C4 107.7(4)
C2 C3 C10 128.6(5)
C4 C3 C10 123.6(4)
C5 C4 C9 119.0(5)
C5 C4 C3 132.5(5)
C9 C4 C3 108.5(4)
C6 C5 C4 119.4(5)
C6 C5 H5 120.3
C4 C5 H5 120.3
C5 C6 C7 121.0(5)
C5 C6 H6 119.5
C7 C6 H6 119.5
C8 C7 C6 121.2(5)
C8 C7 H7 119.4
C6 C7 H7 119.4
C9 C8 C7 117.5(5)
C9 C8 H8 121.3
C7 C8 H8 121.3
C8 C9 N1 131.3(5)
C8 C9 C4 122.0(5)
N1 C9 C4 106.7(4)
C3 C10 H10A 109.5
C3 C10 H10B 109.5
H10A C10 H10B 109.5
C3 C10 H10C 109.5
H10A C10 H10C 109.5
H10B C10 H10C 109.5
C12 C11 C16 119.7(5)
C12 C11 P1 119.0(4)
C16 C11 P1 121.3(4)
C11 C12 C13 120.3(5)
C11 C12 H12 119.9
C13 C12 H12 119.9
C14 C13 C12 120.0(5)
C14 C13 H13 120.0
C12 C13 H13 120.0
C13 C14 C15 120.4(5)
C13 C14 H14 119.8
C15 C14 H14 119.8
C14 C15 C16 120.0(5)
C14 C15 H15 120.0
C16 C15 H15 120.0
C11 C16 C15 119.5(5)
C11 C16 H16 120.2
C15 C16 H16 120.2
C18 C17 C22 118.9(5)
C18 C17 P1 121.8(4)
C22 C17 P1 119.2(5)
C17 C18 C19 120.3(6)
C17 C18 H18 119.9
C19 C18 H18 119.9
C20 C19 C18 120.1(7)
C20 C19 H19 120.0
C18 C19 H19 120.0
C19 C20 C21 120.0(7)
C19 C20 H20 120.0
C21 C20 H20 120.0
C20 C21 C22 121.1(6)
C20 C21 H21 119.5
C22 C21 H21 119.5
C21 C22 C17 119.7(6)
C21 C22 H22 120.1
C17 C22 H22 120.1
C24 C23 N2 109.9(4)
C24 C23 C1 133.6(5)
N2 C23 C1 115.7(4)
C23 C24 C25 106.2(5)
C23 C24 C31 130.6(5)
C25 C24 C31 123.1(4)
C26 C25 C30 119.3(5)
C26 C25 C24 131.6(5)
C30 C25 C24 109.0(4)
C27 C26 C25 118.7(6)
C27 C26 H26 120.7
C25 C26 H26 120.7
C26 C27 C28 121.2(5)
C26 C27 H27 119.4
C28 C27 H27 119.4
C29 C28 C27 121.0(6)
C29 C28 H28 119.5
C27 C28 H28 119.5
C30 C29 C28 116.9(6)
C30 C29 H29 121.5
C28 C29 H29 121.5
C29 C30 C25 122.9(5)
C29 C30 N2 130.0(5)
C25 C30 N2 107.2(5)
C24 C31 H31A 109.5
C24 C31 H31B 109.5
H31A C31 H31B 109.5
C24 C31 H31C 109.5
H31A C31 H31C 109.5
H31B C31 H31C 109.5
C37 C32 C33 118.7(5)
C37 C32 P2 122.9(4)
C33 C32 P2 118.0(4)
C34 C33 C32 120.5(5)
C34 C33 H33 119.8
C32 C33 H33 119.8
C33 C34 C35 120.7(5)
C33 C34 H34 119.7
C35 C34 H34 119.7
C34 C35 C36 119.2(5)
C34 C35 H35 120.4
C36 C35 H35 120.4
C35 C36 C37 120.5(5)
C35 C36 H36 119.8
C37 C36 H36 119.8
C32 C37 C36 120.4(5)
C32 C37 H37 119.8
C36 C37 H37 119.8
C39 C38 C43 118.6(5)
C39 C38 P2 118.0(4)
C43 C38 P2 123.4(5)
C40 C39 C38 121.2(5)
C40 C39 H39 119.4
C38 C39 H39 119.4
C39 C40 C41 120.0(6)
C39 C40 H40 120.0
C41 C40 H40 120.0
C42 C41 C40 119.4(6)
C42 C41 H41 120.3
C40 C41 H41 120.3

C41	C42	C43	120.9(6)	
C41	C42	H42	119.5	
C43	C42	H42	119.5	
C38	C43	C42	119.9(6)	
C38	C43	H43	120.0	
C42	C43	H43	120.0	
C45	C44	N3	109.8(4)	
C45	C44	C1	134.8(5)	
N3	C44	C1	115.0(4)	
C44	C45	C46	107.0(5)	
C44	C45	C52	129.9(5)	
C46	C45	C52	123.0(5)	
C47	C46	C51	120.2(5)	
C47	C46	C45	132.2(5)	
C51	C46	C45	107.6(5)	
C46	C47	C48	118.7(5)	
C46	C47	H47	120.6	
C48	C47	H47	120.6	
C47	C48	C49	120.7(5)	
C47	C48	H48	119.6	
C49	C48	H48	119.6	
C50	C49	C48	121.6(5)	
C50	C49	H49	119.2	
C48	C49	H49	119.2	
C49	C50	C51	117.0(5)	
C49	C50	H50	121.5	
C51	C50	H50	121.5	
C50	C51	N3	130.7(5)	
C50	C51	C46	121.8(5)	
N3	C51	C46	107.5(4)	
C45	C52	H52A	109.5	
C45	C52	H52B	109.5	
H52A	C52	H52B	109.5	
C45	C52	H52C	109.5	
H52A	C52	H52C	109.5	
H52B	C52	H52C	109.5	
C54	C53	C58	119.6(5)	
C54	C53	P3	117.3(4)	
C58	C53	P3	123.0(4)	
C53	C54	C55	119.8(5)	
C53	C54	H54	120.1	
C55	C54	H54	120.1	
C56	C55	C54	120.5(6)	
C56	C55	H55	119.8	
C54	C55	H55	119.8	
C55	C56	C57	120.1(5)	
C55	C56	H56	119.9	
C57	C56	H56	119.9	
C56	C57	C58	120.3(5)	
C56	C57	H57	119.8	
C58	C57	H57	119.8	
C53	C58	C57	119.7(5)	
C53	C58	H58	120.2	
C57	C58	H58	120.2	
C60	C59	C64	119.4(5)	
C60	C59	P3	122.4(4)	
C64	C59	P3	117.9(4)	
C61	C60	C59	120.0(5)	
C61	C60	H60	120.0	
C59	C60	H60	120.0	
C60	C61	C62	120.2(5)	
C60	C61	H61	119.9	
C62	C61	H61	119.9	
C61	C62	C63	119.9(5)	
C61	C62	H62	120.0	
C63	C62	H62	120.0	
C64	C63	C62	120.4(5)	
C64	C63	H63	119.8	
C62	C63	H63	119.8	
C63	C64	C59	120.0(5)	
C63	C64	H64	120.0	
C59	C64	H64	120.0	
O1	C65	Ni1	176.5(5)	
F1A	B1A	F2A	111.1(6)	
F1A	B1A	F4A	111.5(6)	
F2A	B1A	F4A	109.8(5)	
F1A	B1A	F3A	108.3(5)	
F2A	B1A	F3A	108.1(6)	
F4A	B1A	F3A	108.0(6)	
Cl2B	C1B	Cl1B	115.7(10)	
Cl2B	C1B	H1BA	108.4	
Cl1B	C1B	H1BA	108.4	
Cl2B	C1B	H1BB	108.4	
Cl1B	C1B	H1BB	108.4	
H1BA	C1B	H1BB	107.4	
Cl2'	C1B'	Cl1'	111.8(11)	
Cl2'	C1B'	H1BC	109.3	
Cl1'	C1B'	H1BC	109.3	
Cl2'	C1B'	H1BD	109.3	
Cl1'	C1B'	H1BD	109.3	
H1BC	C1B'	H1BD	107.9	
Cl1C	C1C	Cl2C	113.1(10)	
Cl1C	C1C	H1CA	109.0	
Cl2C	C1C	H1CA	109.0	
Cl1C	C1C	H1CB	109.0	
Cl2C	C1C	H1CB	109.0	
H1CA	C1C	H1CB	107.8	
Cl1D	C1D	Cl2D	120.8(15)	
Cl1D	C1D	H1D	119.6	
Cl2D	C1D	H1D	119.6	
C2A	C1A	H1AA	109.5	
C2A	C1A	H1AB	109.5	
H1AA	C1A	H1AB	109.5	
C2A	C1A	H1AC	109.5	
H1AA	C1A	H1AC	109.5	
H1AB	C1A	H1AC	109.5	
C3A	C2A	C1A	107.7(12)	
C3A	C2A	H2AA	110.2	
C1A	C2A	H2AA	110.2	
C3A	C2A	H2AB	110.2	
C1A	C2A	H2AB	110.2	
H2AA	C2A	H2AB	108.5	
C2A	C3A	C4A	108.7(12)	
C2A	C3A	H3AA	110.0	
C4A	C3A	H3AA	110.0	
C2A	C3A	H3AB	110.0	
C4A	C3A	H3AB	110.0	
H3AA	C3A	H3AB	108.3	
C5A	C4A	C3A	105.9(12)	
C5A	C4A	H4AA	110.6	
C3A	C4A	H4AA	110.6	
C5A	C4A	H4AB	110.6	
C3A	C4A	H4AB	110.6	
H4AA	C4A	H4AB	108.7	
C6A	C5A	C4A	102(2)	
C6A	C5A	H5AA	111.4	
C4A	C5A	H5AA	111.4	
C6A	C5A	H5AB	111.4	
C4A	C5A	H5AB	111.4	
H5AA	C5A	H5AB	109.3	
C5A	C6A	H6AA	109.5	
C5A	C6A	H6AB	109.5	
H6AA	C6A	H6AB	109.5	
C5A	C6A	H6AC	109.5	
H6AA	C6A	H6AC	109.5	
H6AB	C6A	H6AC	109.5	
C2H	C1H	H1HA	109.5	
C2H	C1H	H1HB	109.5	
H1HA	C1H	H1HB	109.5	
C2H	C1H	H1HC	109.5	
H1HA	C1H	H1HC	109.5	
H1HB	C1H	H1HC	109.5	
C3H	C2H	C1H	108.9(13)	
C3H	C2H	H2HC	109.9	
C1H	C2H	H2HC	109.9	
C3H	C2H	H2HD	109.9	
C1H	C2H	H2HD	109.9	
H2HC	C2H	H2HD	108.3	
C3H#	C3H	C2H	120(6)	3_766
C3H#	C3H	H3HA	107.4	3_766
C2H	C3H	H3HA	107.4	
C3H#	C3H	H3HB	107.4	3_766
C2H	C3H	H3HB	107.4	
H3HA	C3H	H3HB	107.0	
C2H'	C1H'	H1HD	109.5	
C2H'	C1H'	H1HE	109.5	
H1HD	C1H'	H1HE	109.5	
C2H'	C1H'	H1HF	109.5	
H1HD	C1H'	H1HF	109.5	
H1HE	C1H'	H1HF	109.5	
C3H'	C2H'	C1H'	110.1(12)	
C3H'	C2H'	H2HH	109.6	
C1H'	C2H'	H2HH	109.6	
C3H'	C2H'	H2HI	109.6	
C1H'	C2H'	H2HI	109.7	
H2HH	C2H'	H2HI	108.1	
C2H'	C3H'	C3H'#	100(2)	3_766
C2H'	C3H'	H3HE	111.8	
C3H'#	C3H'	H3HE	111.8	3_766
C2H'	C3H'	H3HD	111.8	
C3H'#	C3H'	H3HD	111.8	3_766
H3HE	C3H'	H3HD	109.5	
C2HH	C1HH	C1HH#	128(6)	3_666
C2HH	C1HH	H1AH	105.3	
C1HH#	C1HH	H1AH	105.3	3_666
C2HH	C1HH	H1BH	105.3	
C1HH#	C1HH	H1BH	105.3	3_666
H1AH	C1HH	H1BH	106.0	
C1HH	C2HH	C3HH	128(4)	
C1HH	C2HH	H2HA	105.3	
C3HH	C2HH	H2HA	105.3	
C1HH	C2HH	H2HB	105.3	
C3HH	C2HH	H2HB	105.3	
H2HA	C2HH	H2HB	106.0	
C2HH	C3HH	H3AH	109.5	

C2HH	C3HH	H3BH	109.5
H3AH	C3HH	H3BH	109.5
C2HH	C3HH	H3HC	109.5
H3AH	C3HH	H3HC	109.5
H3BH	C3HH	H3HC	109.5

Torsion angles [°] for sfl4006nico.

___

C17	P1	N1	C9	83.3(5)
C11	P1	N1	C9	-26.3(5)
Ni1	P1	N1	C9	-149.7(5)
C17	P1	N1	C2	-122.4(4)
C11	P1	N1	C2	128.0(4)
Ni1	P1	N1	C2	4.5(4)
C32	P2	N2	C30	-37.7(5)
C38	P2	N2	C30	72.7(5)
Ni1	P2	N2	C30	-156.1(4)
C32	P2	N2	C23	124.3(4)
C38	P2	N2	C23	-125.3(4)
Ni1	P2	N2	C23	5.9(4)
C9	N1	C2	C3	0.6(6)
P1	N1	C2	C3	-159.5(4)
C9	N1	C2	C1	-174.3(4)
P1	N1	C2	C1	25.6(6)
C44	C1	C2	C3	-99.3(7)
C23	C1	C2	C3	28.5(8)
Ni1	C1	C2	C3	143.1(5)
C44	C1	C2	N1	74.0(5)
C23	C1	C2	N1	-158.1(4)
Ni1	C1	C2	N1	-43.5(5)
C59	P3	N3	C51	89.5(5)
C53	P3	N3	C51	-22.0(5)
Ni1	P3	N3	C51	-148.2(4)
C59	P3	N3	C44	-119.7(4)
C53	P3	N3	C44	128.8(4)
Ni1	P3	N3	C44	2.6(4)
N1	C2	C3	C4	-0.8(6)
C1	C2	C3	C4	173.0(5)
N1	C2	C3	C10	-177.6(5)
C1	C2	C3	C10	-3.8(10)
C2	C3	C4	C5	-178.8(6)
C10	C3	C4	C5	-1.8(10)
C2	C3	C4	C9	0.7(6)
C10	C3	C4	C9	177.7(5)
C9	C4	C5	C6	-1.3(9)
C3	C4	C5	C6	178.2(6)
C4	C5	C6	C7	1.1(10)
C5	C6	C7	C8	-0.6(11)
C6	C7	C8	C9	0.3(10)
C7	C8	C9	N1	-178.3(6)
C7	C8	C9	C4	-0.5(9)
C2	N1	C9	C8	177.9(6)
P1	N1	C9	C8	-26.3(9)
C2	N1	C9	C4	-0.1(6)
P1	N1	C9	C4	155.7(4)
C5	C4	C9	C8	1.0(8)
C3	C4	C9	C8	-178.6(5)
C5	C4	C9	N1	179.3(5)
C3	C4	C9	N1	-0.3(6)
N1	P1	C11	C12	-99.1(4)
C17	P1	C11	C12	149.6(4)
Ni1	P1	C11	C12	12.7(4)
N1	P1	C11	C16	84.4(4)
C17	P1	C11	C16	-26.9(4)
Ni1	P1	C11	C16	-163.8(3)
C16	C11	C12	C13	-3.1(7)
P1	C11	C12	C13	-179.7(4)
C11	C12	C13	C14	1.0(8)
C12	C13	C14	C15	0.4(8)
C13	C14	C15	C16	0.3(8)
C12	C11	C16	C15	3.7(7)
P1	C11	C16	C15	-179.8(4)
C14	C15	C16	C11	-2.3(7)
N1	P1	C17	C18	18.0(5)
C11	P1	C17	C18	132.4(5)
Ni1	P1	C17	C18	-93.2(5)
N1	P1	C17	C22	-166.7(4)
C11	P1	C17	C22	-52.3(5)
Ni1	P1	C17	C22	82.0(5)
C22	C17	C18	C19	0.2(9)
P1	C17	C18	C19	175.5(6)
C17	C18	C19	C20	0.0(12)
C18	C19	C20	C21	0.5(13)
C19	C20	C21	C22	-1.1(12)
C20	C21	C22	C17	1.3(10)
C18	C17	C22	C21	-0.8(8)
P1	C17	C22	C21	-176.3(5)
C30	N2	C23	C24	3.0(5)
P2	N2	C23	C24	-163.4(3)
C30	N2	C23	C1	-168.0(4)
P2	N2	C23	C1	25.6(5)
C2	C1	C23	C24	-99.4(6)
C44	C1	C23	C24	29.2(7)
Ni1	C1	C23	C24	146.1(5)
C2	C1	C23	N2	68.9(5)
C44	C1	C23	N2	-162.5(4)
Ni1	C1	C23	N2	-45.6(4)
N2	C23	C24	C25	-2.1(5)
C1	C23	C24	C25	166.7(5)
N2	C23	C24	C31	-177.5(5)
C1	C23	C24	C31	-8.7(9)
C23	C24	C25	C26	-177.3(5)
C31	C24	C25	C26	-1.5(9)
C23	C24	C25	C30	0.5(5)
C31	C24	C25	C30	176.4(5)
C30	C25	C26	C27	-1.3(7)
C24	C25	C26	C27	176.3(5)
C25	C26	C27	C28	0.6(8)
C26	C27	C28	C29	0.7(9)
C27	C28	C29	C30	-1.3(8)
C28	C29	C30	C25	0.6(8)
C28	C29	C30	N2	-177.8(5)
C26	C25	C30	C29	0.7(8)
C24	C25	C30	C29	-177.4(5)
C26	C25	C30	N2	179.4(4)
C24	C25	C30	N2	1.3(5)
C23	N2	C30	C29	176.0(5)
P2	N2	C30	C29	-20.7(8)
C23	N2	C30	C25	-2.6(5)
P2	N2	C30	C25	160.7(4)
N2	P2	C32	C37	-22.1(5)
C38	P2	C32	C37	-133.7(4)
Ni1	P2	C32	C37	86.4(4)
N2	P2	C32	C33	164.5(4)
C38	P2	C32	C33	52.9(5)
Ni1	P2	C32	C33	-86.9(4)
C37	C32	C33	C34	2.3(8)
P2	C32	C33	C34	175.9(5)
C32	C33	C34	C35	-0.4(9)
C33	C34	C35	C36	-0.8(9)
C34	C35	C36	C37	0.0(8)
C33	C32	C37	C36	-3.1(8)
P2	C32	C37	C36	-176.4(4)
C35	C36	C37	C32	1.9(8)
N2	P2	C38	C39	84.7(4)
C32	P2	C38	C39	-162.6(4)
Ni1	P2	C38	C39	-29.2(5)
N2	P2	C38	C43	-92.1(5)
C32	P2	C38	C43	20.6(5)
Ni1	P2	C38	C43	154.0(4)
C43	C38	C39	C40	-1.3(8)
P2	C38	C39	C40	-178.3(4)
C38	C39	C40	C41	0.6(8)
C39	C40	C41	C42	0.7(10)
C40	C41	C42	C43	-1.1(11)
C39	C38	C43	C42	0.9(9)
P2	C38	C43	C42	177.7(5)
C41	C42	C43	C38	0.4(11)
C51	N3	C44	C45	-0.6(5)
P3	N3	C44	C45	-158.1(3)
C51	N3	C44	C1	-174.6(4)
P3	N3	C44	C1	27.8(5)
C2	C1	C44	C45	25.4(8)
C23	C1	C44	C45	-102.5(6)
Ni1	C1	C44	C45	141.7(5)
C2	C1	C44	N3	-162.5(4)
C23	C1	C44	N3	69.6(5)
Ni1	C1	C44	N3	-46.1(5)
N3	C44	C45	C46	0.1(5)
C1	C44	C45	C46	172.6(5)
N3	C44	C45	C52	-175.3(5)
C1	C44	C45	C52	-2.9(10)
C44	C45	C46	C47	-178.8(6)
C52	C45	C46	C47	-3.0(9)
C44	C45	C46	C51	0.4(6)
C52	C45	C46	C51	176.2(5)
C51	C46	C47	C48	0.3(8)
C45	C46	C47	C48	179.4(6)
C46	C47	C48	C49	0.2(9)
C47	C48	C49	C50	-0.6(9)
C48	C49	C50	C51	0.4(8)
C49	C50	C51	N3	-178.7(5)
C49	C50	C51	C46	0.0(8)
C44	N3	C51	C50	179.7(5)
P3	N3	C51	C50	-28.1(8)
C44	N3	C51	C46	0.8(5)
P3	N3	C51	C46	153.0(4)
C47	C46	C51	C50	-0.4(8)
C45	C46	C51	C50	-179.7(5)
C47	C46	C51	N3	178.6(5)
C45	C46	C51	N3	-0.7(5)
N3	P3	C53	C54	-64.4(4)
C59	P3	C53	C54	-177.5(4)
Ni1	P3	C53	C54	48.3(5)
N3	P3	C53	C58	111.7(4)
C59	P3	C53	C58	-1.4(5)
Ni1	P3	C53	C58	-135.7(4)
C58	C53	C54	C55	-1.9(8)
P3	C53	C54	C55	174.3(5)

C53	C54	C55	C56	1.3(9)
C54	C55	C56	C57	-0.6(9)
C55	C56	C57	C58	0.3(9)
C54	C53	C58	C57	1.7(8)
P3	C53	C58	C57	-174.3(4)
C56	C57	C58	C53	-0.9(8)
N3	P3	C59	C60	15.1(5)
C53	P3	C59	C60	124.5(4)
Ni1	P3	C59	C60	-98.4(4)
N3	P3	C59	C64	-171.2(4)
C53	P3	C59	C64	-61.7(4)
Ni1	P3	C59	C64	75.4(4)

C64	C59	C60	C61	0.5(8)
P3	C59	C60	C61	174.2(4)
C59	C60	C61	C62	1.5(8)
C60	C61	C62	C63	-1.1(8)
C61	C62	C63	C64	-1.4(8)
C62	C63	C64	C59	3.4(8)
C60	C59	C64	C63	-3.0(8)
P3	C59	C64	C63	-177.0(4)
C1A	C2A	C3A	C4A	-172(3)
C2A	C3A	C4A	C5A	124(3)
C3A	C4A	C5A	C6A	146(3)
C1H	C2H	C3H	C3H#	-179(7)

3_766
C1H' C2H' C3H' C3H'# -170(3)
3_766
C1HH# C1HH C2HH C3HH 160(6)
3_666

Symetry operations
____
1 'x, y, z'
2 '-x+1/2, y+1/2, -z+1/2'
3 '-x, -y, -z'
4 'x-1/2, -y-1/2, z-1/2'

4. Electrochemistry

4.1. Cyclic voltammetry and electrolysis results

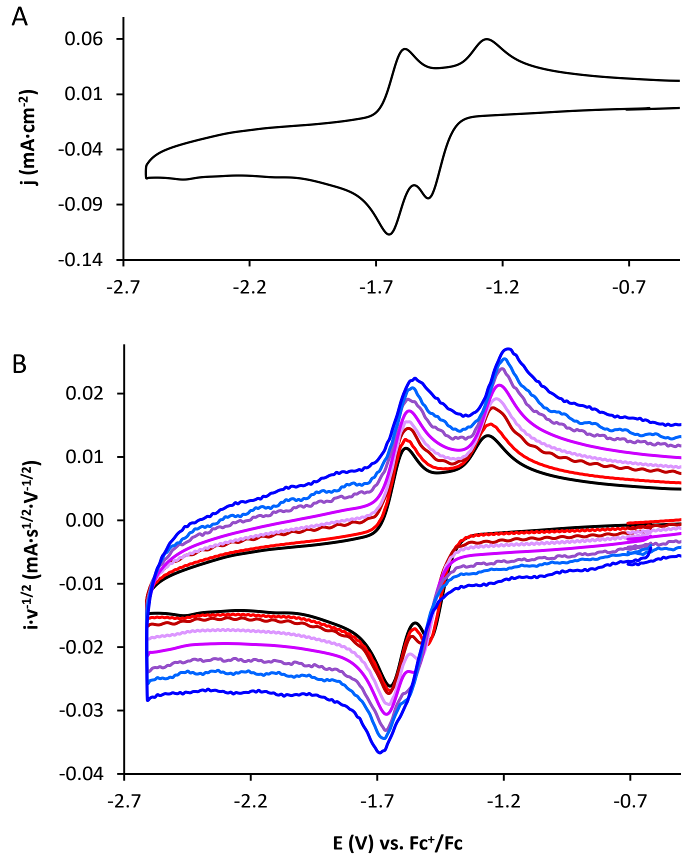

Figure S 5. CVs of **Ni^{H}_{Br}** (0.5 mM) under argon atmosphere. A) Current density *vs* redox potential plot at v = 0.1 V·s^{-1}. B) Scan rate normalized current *vs* redox potential at v (V·s^{-1}) = 0.1 (black), 0.2 (red), 0.6 (dark red), 1 (pink), 2 (magenta), 4 (purple), 6 (light blue), 10 (dark blue). Conditions: glassy carbon (ø = 0.3 cm) in 0.1 M $TBAPF_6$/DMF electrolyte.

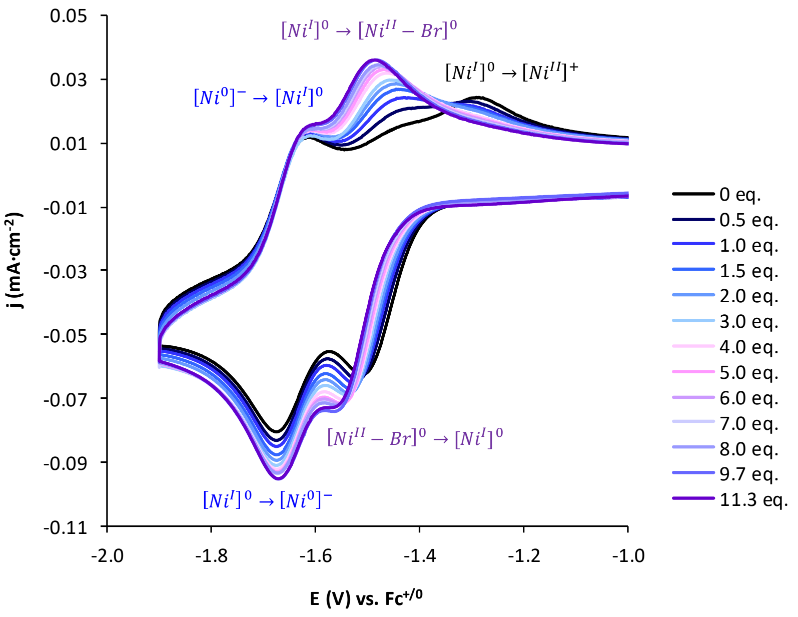

Figure S 6. CVs of $\mathbf{Ni^H_{Br}}$ (0.5 mM) with increasing ammounts of TBABr under argon atmosphere over glassy carbon (ø = 0.3 cm) in 0.1 M $TBAPF_6$/DMF electrolyte. Conditions: glassy carbon (ø = 0.3 cm) in 0.1 M $TBAPF_6$/DMF electrolyte.

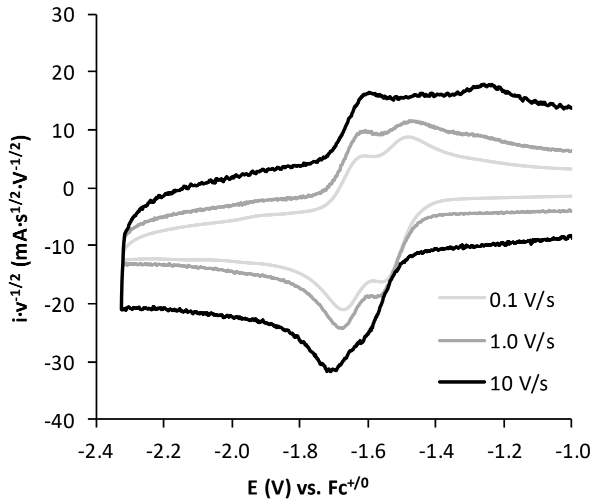

Figure S 7. CVs of $\mathbf{Ni^H_{Br}}$ (0.5 mM) in the presence of 8 eq. of TBABr at 0.1, 1 and 10 V s^{-1}. The CV at fast scan rates reveals again the oxidation peak observed without added TBABr at 0.1 V/s. This is due to the fast 1e^- oxidation of the electrochemically formed $[Ni^I]^0$ at the second wave. Conditions: glassy carbon (ø = 0.3 cm) in 0.1 M $TBAPF_6$/DMF electrolyte.

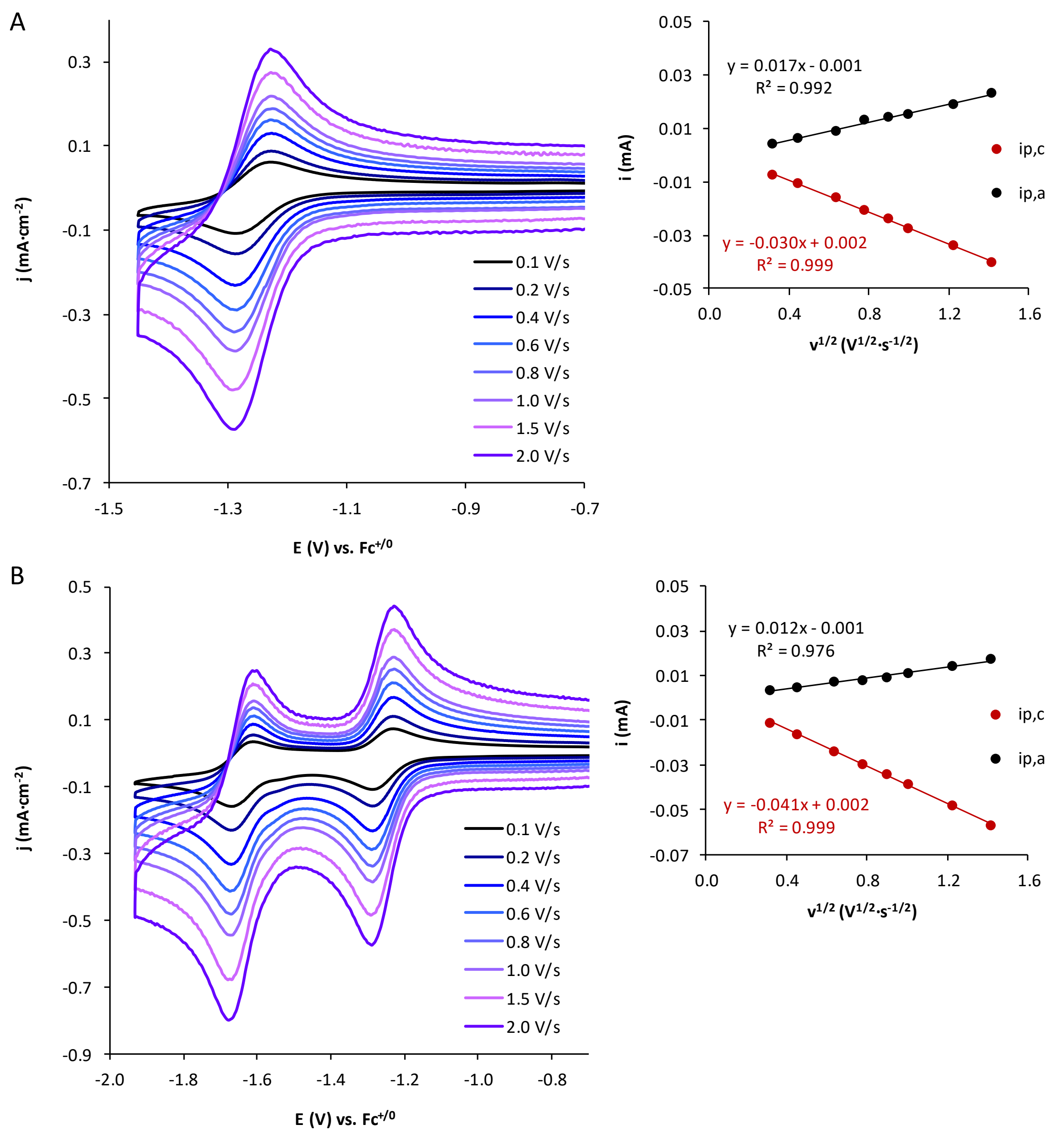

Figure S 8. CVs of $\mathbf{Ni^{H}_{MeCN}}$ (0.5 mM) under Ar in anhydrous solvent at increasing scan rates. A) Analysis of the $Ni^{+/0}$ wave. B) Analysis of the $Ni^{0/-}$ wave. Conditions: glassy carbon (ø = 0.3 cm) in 0.1 M $TBAPF_6$/DMF electrolyte.

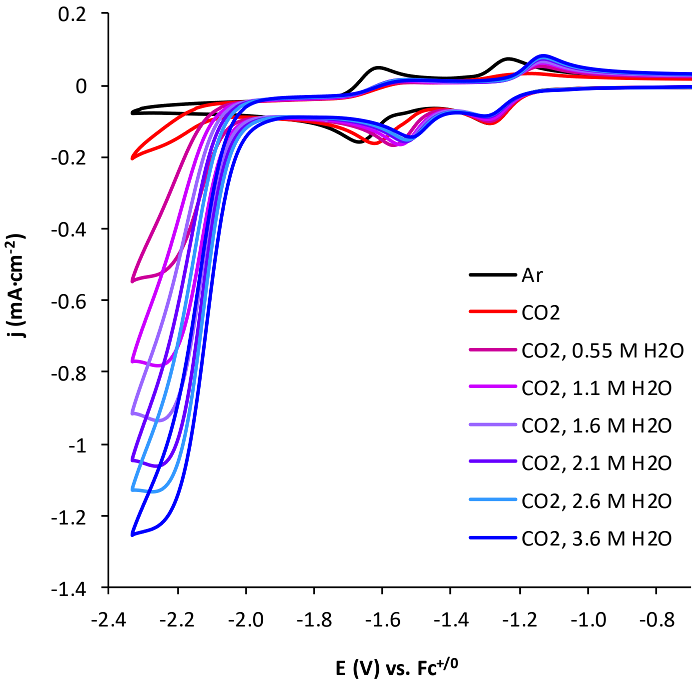

Figure S 9. CVs of **Ni^{H}$_{MeCN}$** (0.5 mM) under Ar (black) and CO_2 (red) in anhydrous solvent and under CO_2 with increasing ammounts of water (0.55 – 3.6 M H_2O). Conditions: glassy carbon (ø = 0.3 cm) in 0.1 M $TBAPF_6$/DMF electrolyte.

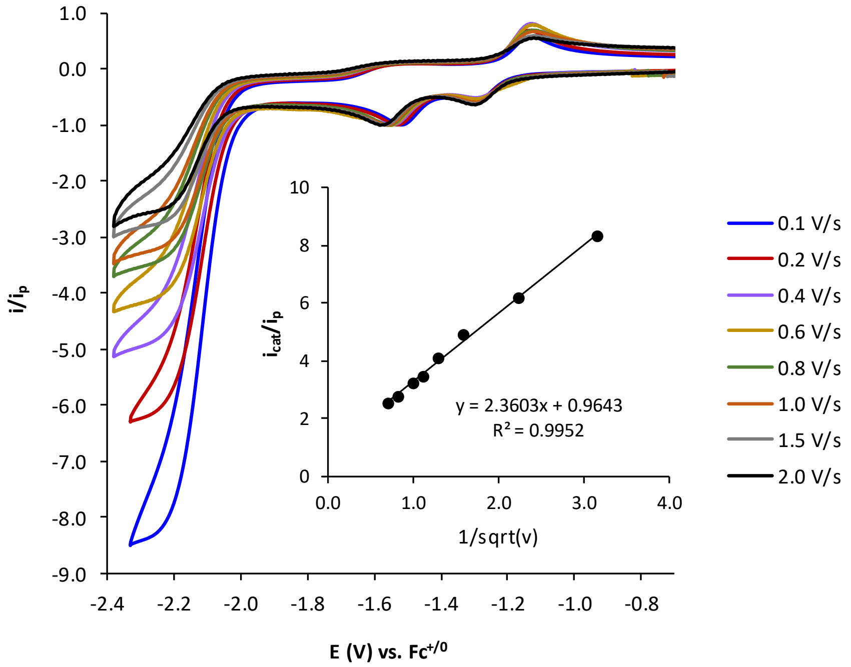

Figure S 10. CVs of **Ni^{H}$_{MeCN}$** (0.5 mM) under CO_2 with 3.6 M H_2O at increasing scan rates (0.1 – 2 V/s). Conditions: glassy carbon (ø = 0.3 cm) in 0.1 M $TBAPF_6$/DMF electrolyte.

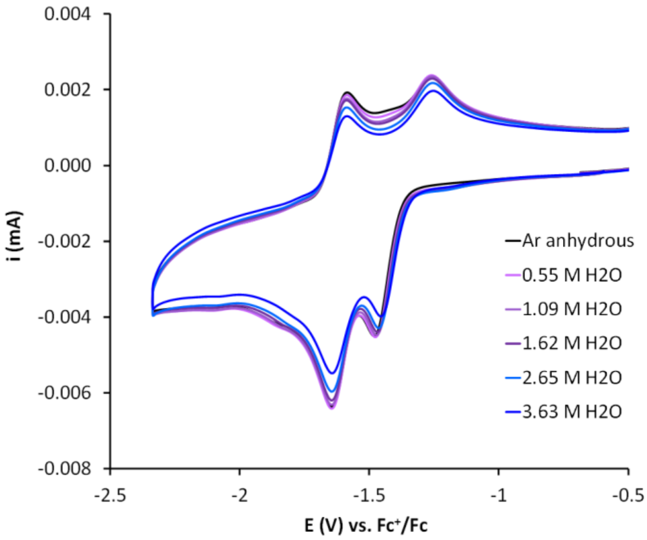

Figure S 11. CVs of **Ni$^{H}_{Br}$** (0.5 mM) under Ar atmosphere with increasing ammounts of added water (0.55 – 3.6 M H_2O). Conditions: glassy carbon (ø = 0.3 cm) in 0.1 M $TBAPF_6$/DMF electrolyte.

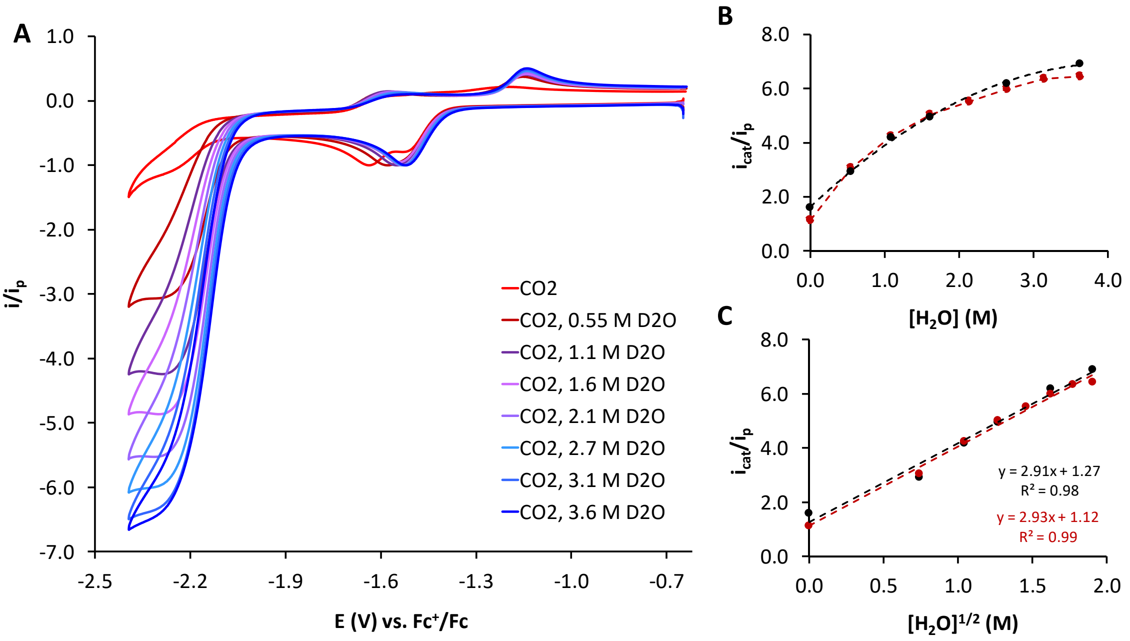

Figure S 12. A) CV of **Ni$^{H}_{Br}$** (0.5 mM) in CO_2-saturated electrolyte without added D_2O (red) and with increasing concentration of added D_2O. B) Plot of the i_{cat}/i_p vs. [water]. C) Plot of the i_{cat}/i_p vs. the square root of [water] and linear fitting. Comparison between D_2O (red) and H_2O (black). Conditions: glassy carbon (ø = 0.3 cm) in 0.1 M $TBAPF_6$/DMF electrolyte.

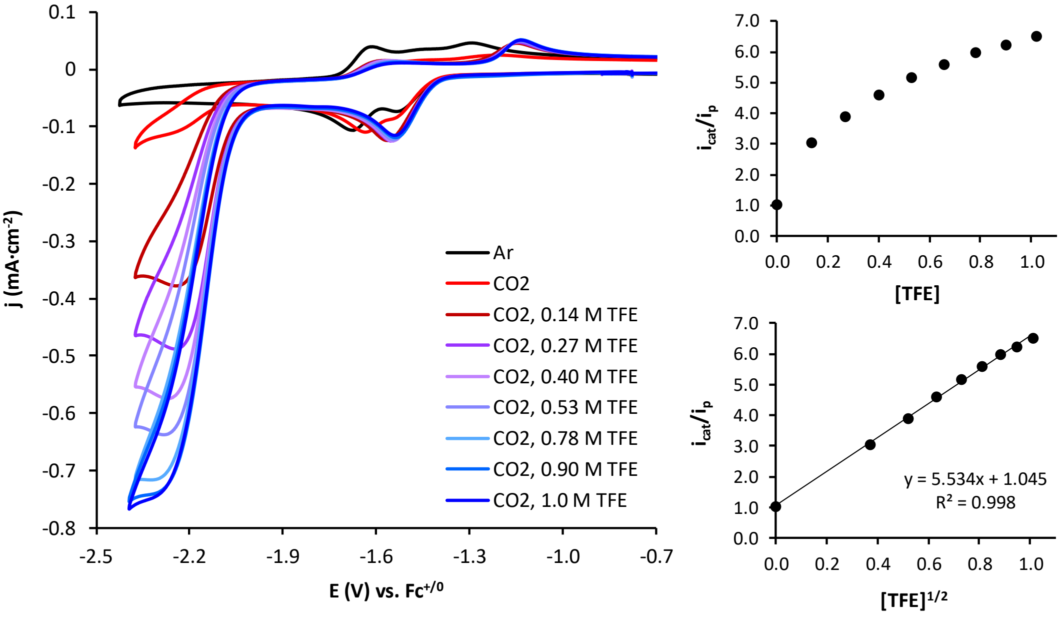

Figure S 13. A) CV of $\mathbf{Ni^{H}_{Br}}$ (0.5 mM) under Ar (black) and in CO_2-saturated DMF/$TBAPF_6$ 0.1 M electrolyte without added TFE (red) and with increasing concentration of added TFE. B) Plot of the i_{cat}/i_p *vs.* [TFE]. C) Plot of the i_{cat}/i_p *vs.* the square root of [TFE] and linear fitting.

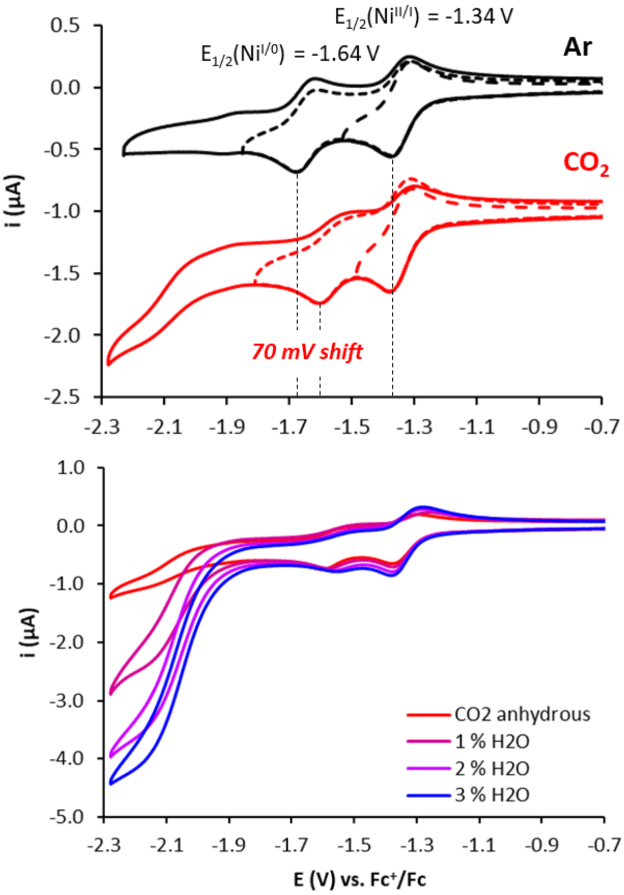

Figure S 14. CVs of $\mathbf{Ni^{H}_{MeCN}}$ (0.5 mM). Top) Under Ar and CO_2 in anhydrous solvent. Bottom) Under CO_2 with added water (from 1 to 3 %). Conditions: glassy carbon (ø = 0.3 cm) in MeCN/$TBAPF_6$ 0.1 M electrolyte.

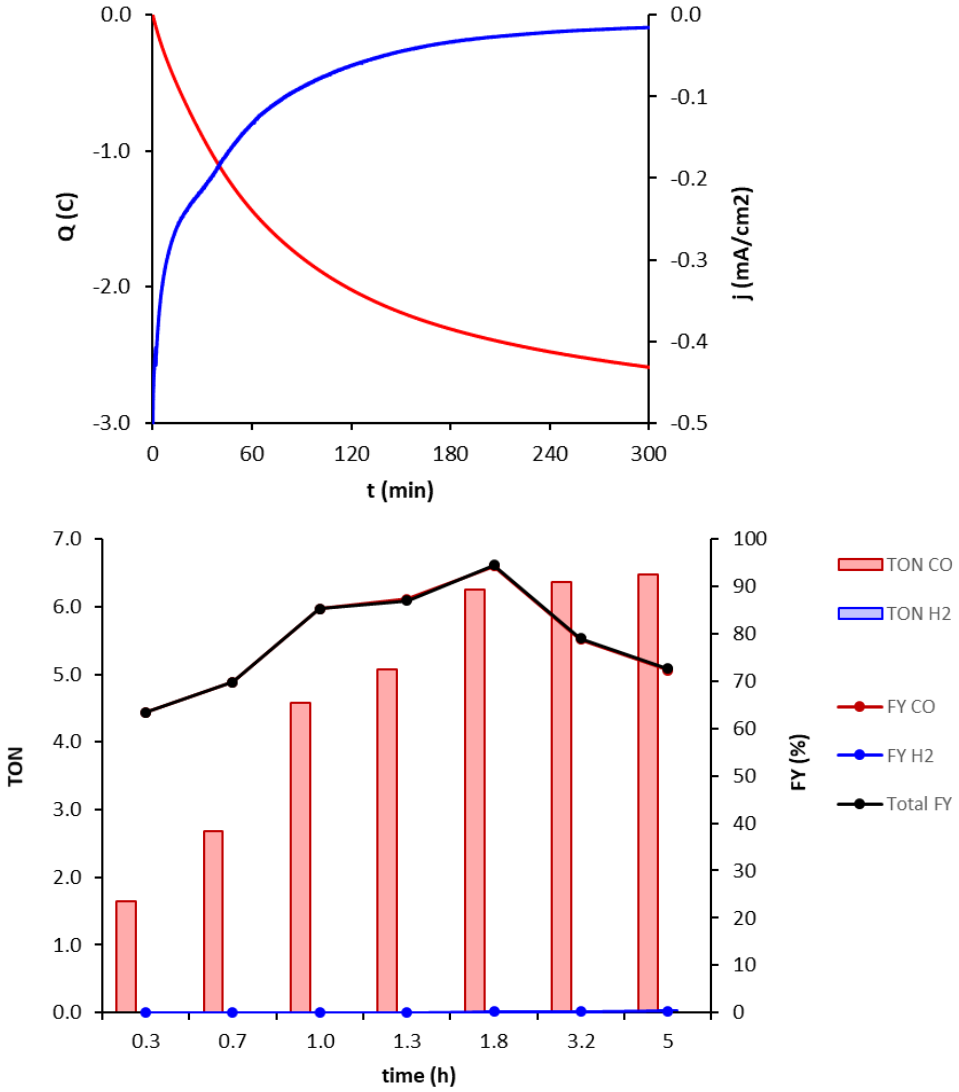

Figure S 15. CPE data of **Ni$^{H}_{Br}$** (0.5 mM) in DMF/$TBAPF_6$ 0.1 M under CO_2 with added water (3.5 M). Top) Charge and current *versus* time profile. Bottom) Quantification of gas products by GC-TCD: TON and FY% *versus* time. Conditions: E_{app} = -2.3 V vs. $Fc^{+/0}$, [H_2O] = 3.5 M in a divided cell; GC Rod Area = 1.8 cm^2 (WE), Pt wire (CE) and Ag wire (pseudo)reference (CE) using the $Fc^{+/0}$ couple as an external standard.

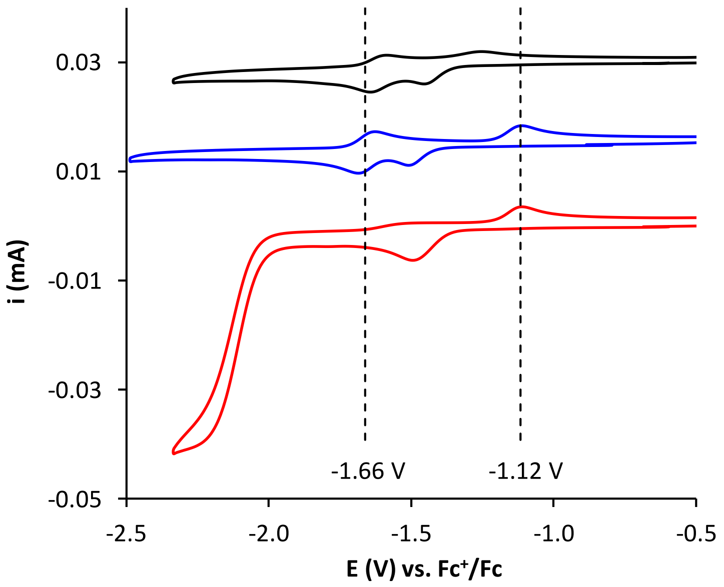

Figure S 16. A) CV of **Ni^{H}$_{Br}$** (0.5 mM) under Ar (black), CO (blue) and CO_2 (red). Conditions: glassy carbon (ø = 0.3 cm) in 0.1 M TBAPF$_6$/DMF electrolyte.

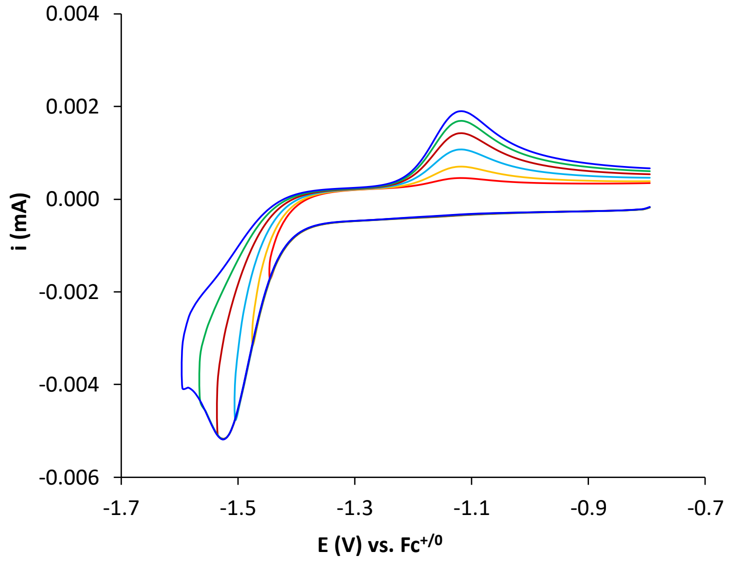

Figure S 17. CV of **Ni^{H}$_{Br}$** (0.5 mM) under CO. Conditions: glassy carbon (ø = 0.3 cm) in 0.1 M TBAPF$_6$/DMF electrolyte.

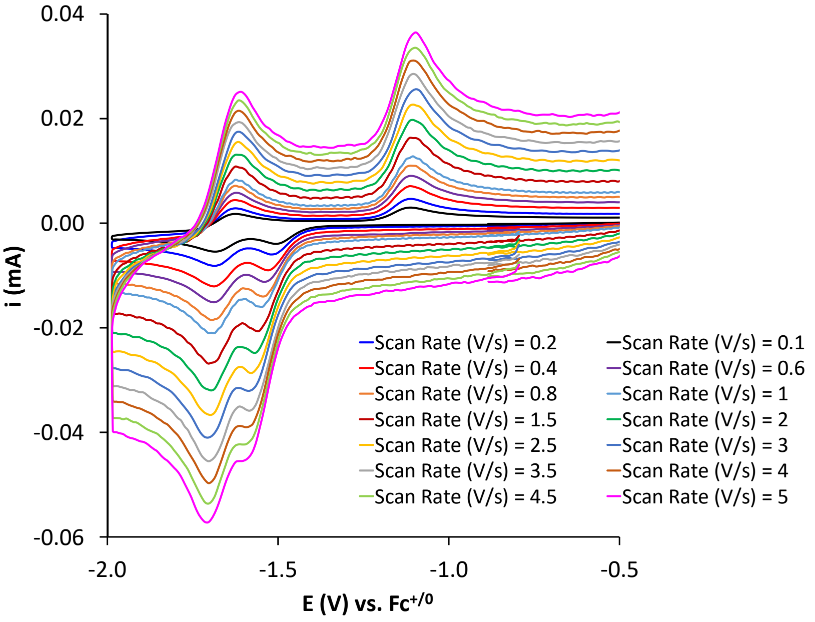

Figure S 18. A) CV of **Ni^{H}$_{Br}$** (0.5 mM) under CO at increasing scan rates. Conditions: glassy carbon (ø = 0.3 cm) in 0.1 M $TBAPF_6$/DMF electrolyte.

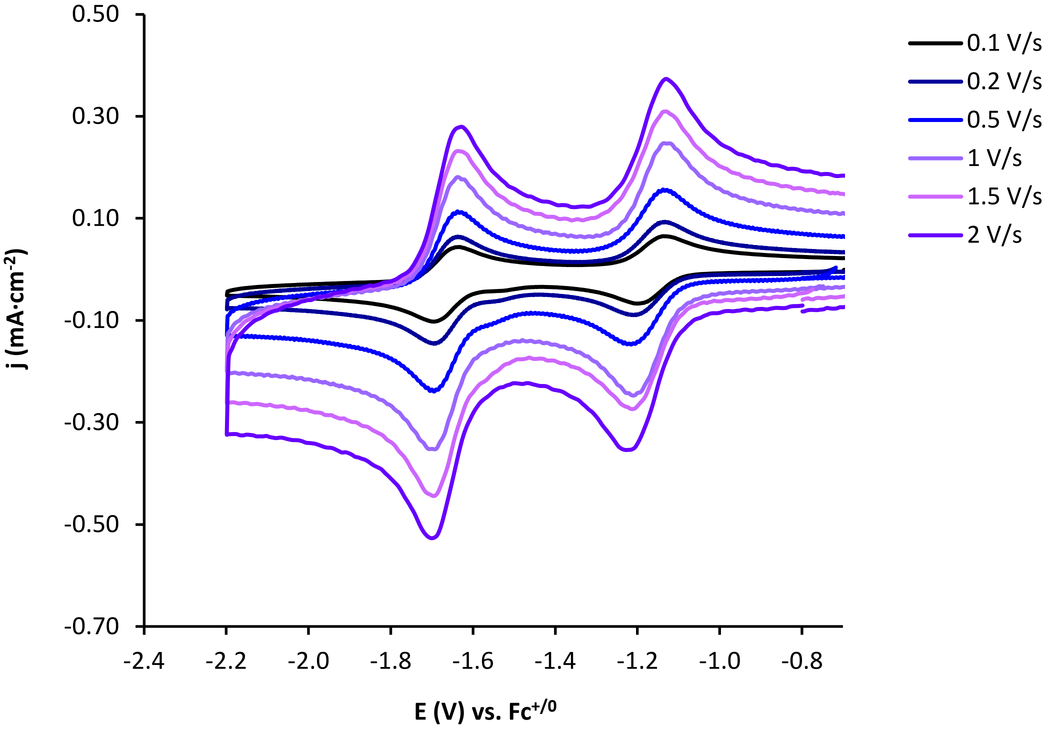

Figure S 19. A) CV of **Ni^{H}$_{CO}$** (0.5 mM) under Ar at increasing scan rates. Conditions: glassy carbon (ø = 0.3 cm) in 0.1 M $TBAPF_6$/DMF electrolyte.

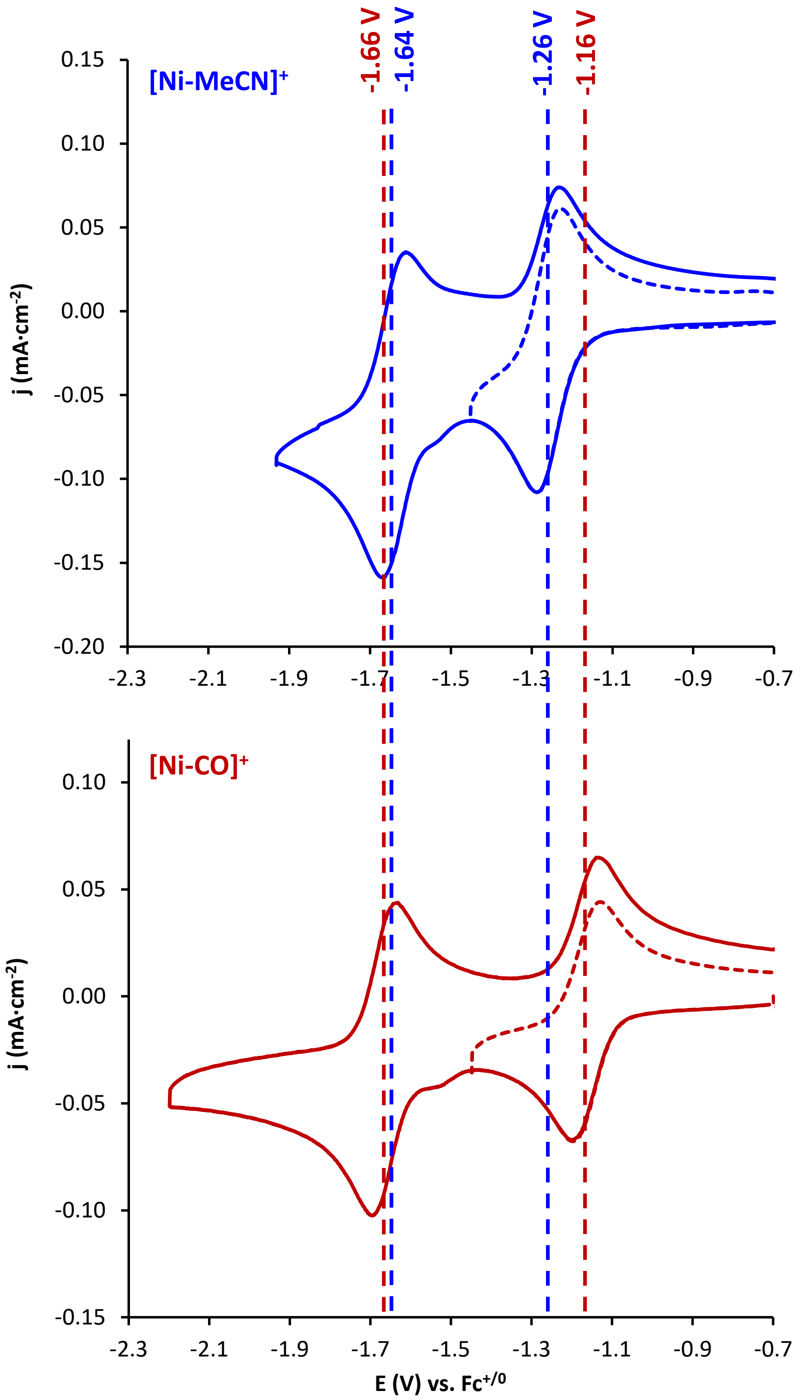

Figure S 20. Comparison between the voltammograms of **Ni^{H}_{MeCN}** (top, blue) and **Ni^{H}_{CO}** (bottom, red) under Ar at 100 $mV{\cdot}s^{-1}$. Conditions: glassy carbon (ø = 0.3 cm), 0.5 mM catalyst concentration in 0.1 M $TBAPF_6$/DMF electrolyte.

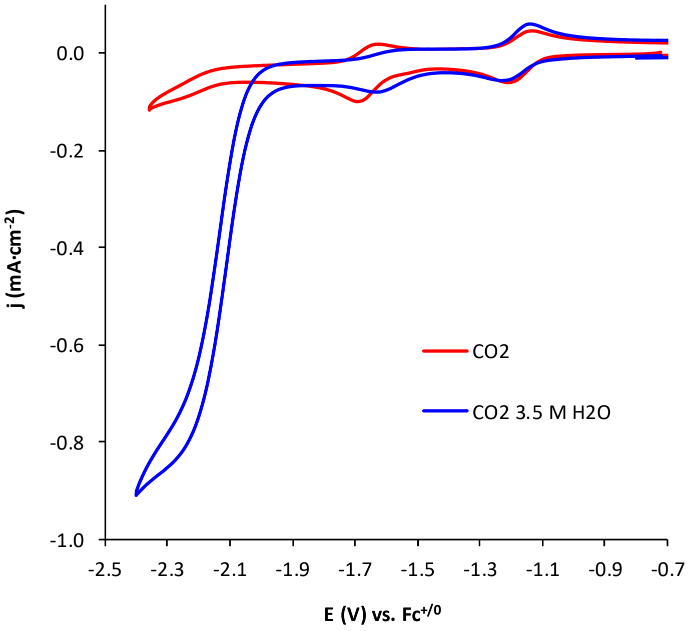

Figure S 21. CVs of $\mathbf{Ni^{H}_{CO}}$ (0.5 mM) under CO_2 without (red) and with added water (3.5 M, blue). Conditions: glassy carbon (ø = 0.3 cm) in 0.1 M $TBAPF_6$/DMF electrolyte. Conditions: glassy carbon (ø = 0.3 cm) in 0.1 M $TBAPF_6$/DMF electrolyte.

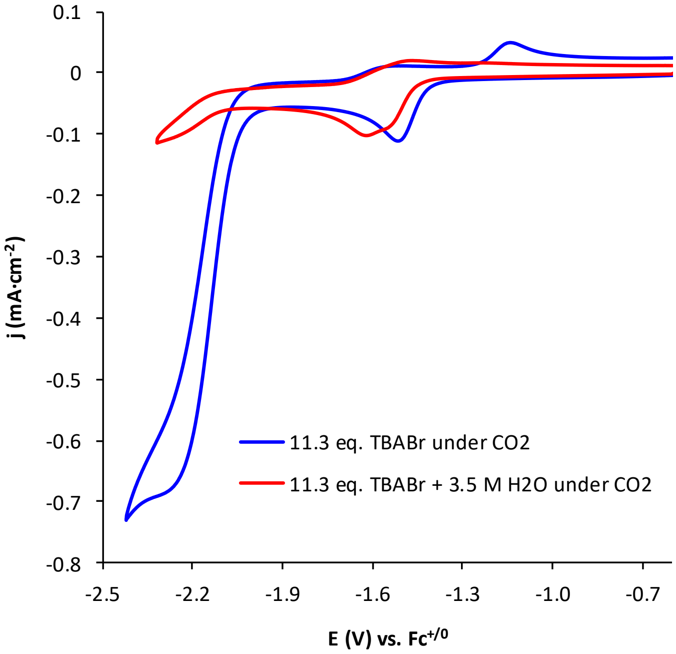

Figure S 22. CVs of $\mathbf{Ni^{H}_{Br}}$ (0.5 mM) in the presence of an excess of TBABr under CO_2 without (red) and with added water (3.5 M, blue). Conditions: glassy carbon (ø = 0.3 cm) in 0.1 M $TBAPF_6$/DMF electrolyte. Conditions: glassy carbon (ø = 0.3 cm) in 0.1 M $TBAPF_6$/DMF electrolyte.

4.2. Estimation of rate constants by the working curve method

The rate constant (*k*) of the chemically irreversible step of an EC process can be detemined by employing the following working curve:[2]

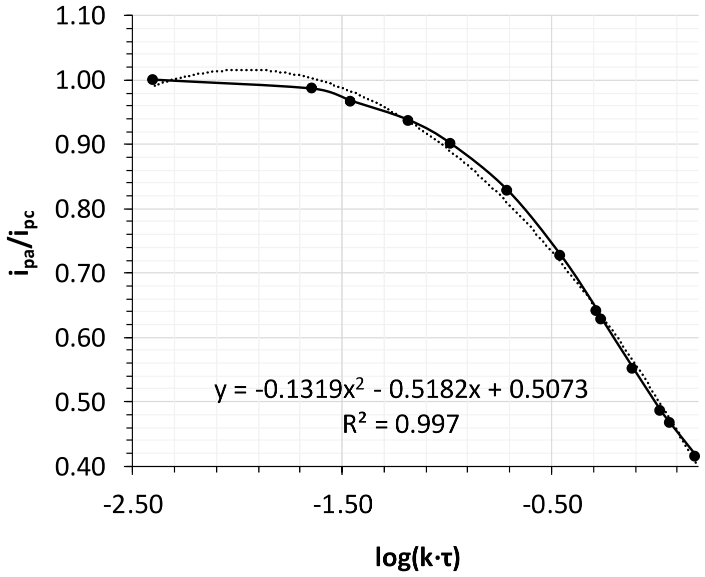

Figure S 23. Working curve employed to obtain the kinetic constant (*k*) of an EC process in a quasi-reversible CV. A quadratic function has been fitted to the working curve.

The working curve in Figure S21 relates the anodic and cathodic peak current ratio with the logarithm of *k*τ, where *k* is the kinetic constant of the chemical step and τ is the time from the $E_{1/2}$ of the themodynamic electrochemical process and the switching potential at a given scan rate.[3] This method can be applied for quasi reversible CV (i. e. in the KO region of the zone diagram) like the one showed in Figure S22. For a more accurate description of the i_{pa}/i_{pc} ratio, we have considered the following semi-empirical equation:[4]

$$\frac{i_{pa}}{i_{pc}} = \frac{(i_{pa})_0}{(i_{pc})_0} + \frac{0.485(i_{ps})_0}{(i_{pc})_0} + 0.086 \quad (S1)$$

where $(i_{pa})_0$ is the anodic current, $(i_{pc})_0$ is the cathodic current and $(i_{ps})_0$ is the switching potential current, with respect the baseline defined in the forward sweep.

By playing with the scan rate and eq. S1, different i_{pa}/i_{pc} values have been experimentally measured. These values have been graphically identified with a log(*k*τ) value in the empirical working curve (Figure S21). Also, the parameter τ has different value at each scan rate; the higher is the scan rate (faster CV) the lower is τ. Table S3 summarizes the experimental i_{pa}/i_{pc} and τ (see tau column) values as well as the log(*k*τ) estimated from the working curve.

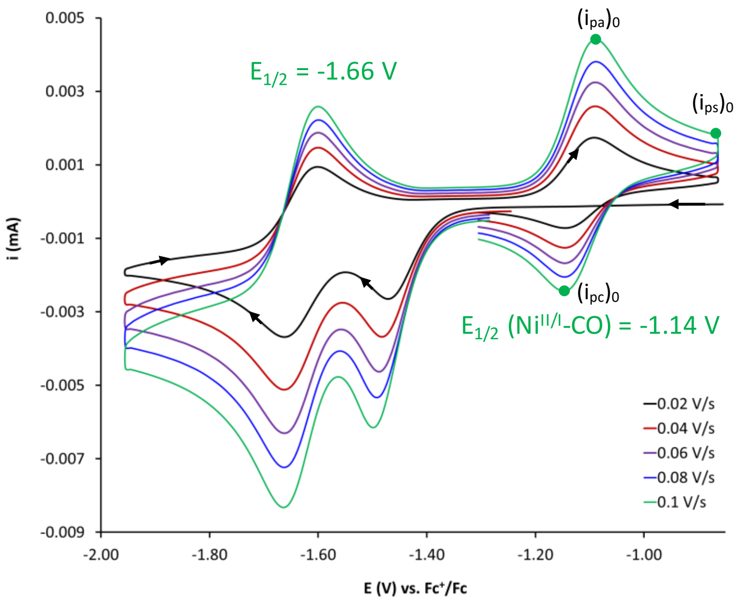

Figure S 24. A) CV of $\mathbf{Ni^{H}_{Br}}$ (0.5 mM) under CO at increasing scan rate in saturated DMF/TBAPF$_6$ 0.1 M electrolyte.

Table S 3. Data obtained from the working curve analysis of the CVs in Figure S23.

v (V/s)	sqrt(v)	(ipa)0	(ipc)0	(ips)0	ipc/ipa	log(k*tau)	tau*k	tau (s)	k (s-1)
0.02	0.14	0.0017	0.0007	0.0007	0.70	-0.40	0.40	13.00	0.03
0.04	0.20	0.0026	0.0013	0.0010	0.76	-0.50	0.32	6.50	0.05
0.06	0.24	0.0032	0.0017	0.0013	0.80	-0.64	0.23	4.33	0.05
0.08	0.28	0.0038	0.0021	0.0016	0.82	-0.70	0.20	3.25	0.06
0.10	0.32	0.0044	0.0024	0.0018	0.84	-0.72	0.19	2.60	0.07

From the data in Table 1, we can estimate an average *k* constant of **0.05 ± 0.02** s^{-1}.

4.3. FTIR Spectroelectrochemistry

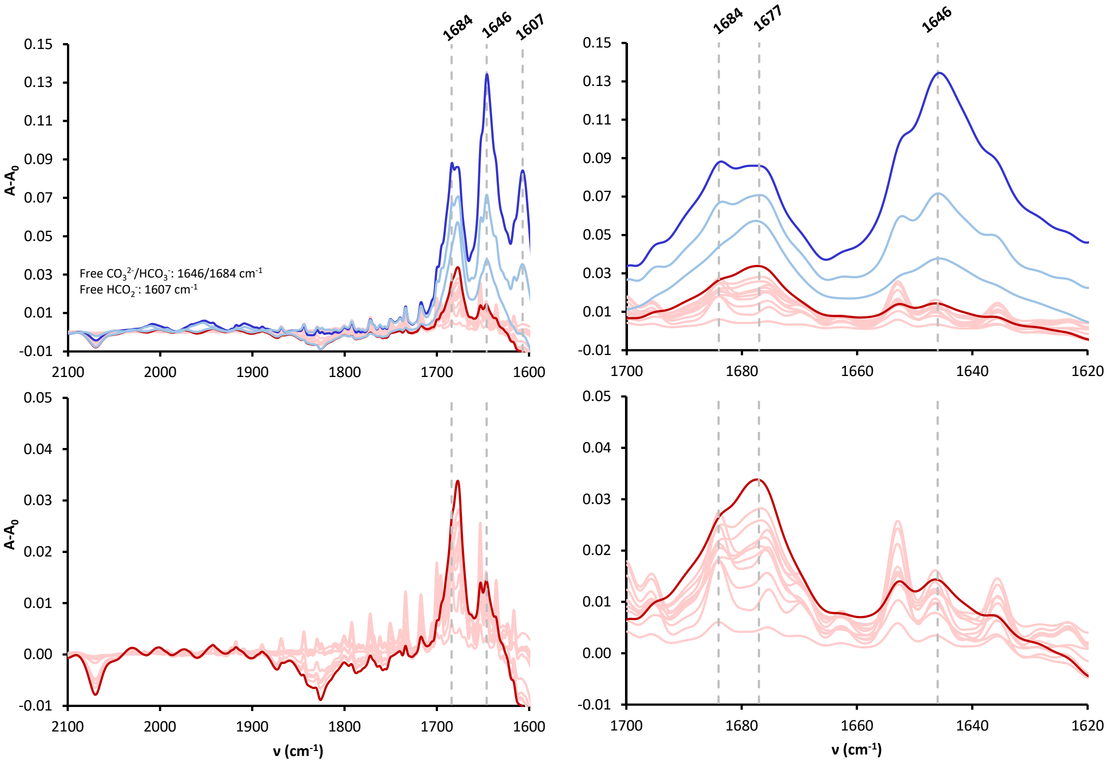

Figure S 25. Left) Carbonyl and carboxylate spectral region of an FTIR-SEC experiment of a **Ni^{H}_{MeCN}** (4 mM) solution in CO_2-saturated electrolyte mixture (in acetonitrile). Right) Magnification in the carboxylate spectral window. Red traces correspond to the non-catalytic faradaic region between ca. -1.2 and -1.6 V vs $Fc^{+/0}$, blue traces correspond to catalytically relevant potentials (<-1.6 V vs $Fc^{+/0}$).

5. DFT calculations

5.1. Computational details

DFT calculations have been performed with the *Gaussian09* and *Gaussian16* software packages.[5] Geometry optimizations and frequency calculations at the ground state structure have been performed at the B3LYP/6-31G* level of theory. Solvent effects and London interactions are considered through the SMD[6] model for acetonitrile and Grimme-D_3 correction[7], respectively. The free energy (G) was calculated following equation *S1*

$$G = E_{elec} + \Delta G_{corr} + \Delta G^{o/*} \qquad (S2)$$

in which E_{elec} is the MN15/6-311++G** single point energy at the B3LYP/6-31G* ground state geometry and ΔG_{corr} is the Gibbs energy correction obtained from the frequency calculation. $\Delta G^{o/*}$ is the free energy change associated with the conversion from the standard-state gas-phase pressure of 1 atm to the desired concentration in mol·L^{-1} according to equation S2

$$\Delta G^{o/*} = RT \ln(24.4 \cdot c) \qquad (S3)$$

where R is the universal gas constant (1.987 cal·mol^{-1}·K^{-1}), T is the temperature in Kelvin and *c* the concentration in mol·L^{-1}. Its value at 1 M concentration and 298.15 K is 1.89 kcal·mol^{-1}. All reactants and products have been corrected by an experimentally meaningful concentration ($[H_2O]_3$ = $[HCO_3(H_2O)]^-$ = 1.2 M; [CO_2] = 0.28 M; [CO] = 0.028 M).

The redox potentials (E°) have been evaluated through the Nernst equation in standard state conditions and using the Standard Hydrogen Electrode (SHE) as the reference.[8] To compare with the experimental values, the potentials are reported versus the $Fc^{+/0}$ reference.[9]

$$E^{o}(V) = -\left(\frac{\Delta G^{\circ}}{nF} - \frac{\Delta G^{\circ}_{SHE}}{F}\right) - 0.624\ V \qquad (S4)$$

where *n* is the number of electrons involved in the reduction step, *F* is the Faraday constant, ΔG°_{SHE} = -4.28 eV and 0.624 V is the $E_{1/2}(V)\ vs\ SHE$ of the $Fc^{+/0}$ redox pair in acetonitrile.

5.2. Complementary DFT results

Table S 4. Theoretical $Ni^{0/-}$ redox potentials (V vs. $Fc^{+/0}$) calculated with different combinations of DFT functionals and basis sets.

	Method	E(V)
A	B3LYP/6-31G*	-2.49
B	B3LYP/6-311++G**//B3LYP/6-31G*	-2.24
C	B3LYP/6-311++G**^{NiPC} 6-311G**^{NCH}//B3LYP/6-31G*	-2.26
D	B3LYP/ma-def2-TVPZ^{NiPC} def2-TVPZ^{NCH}//B3LYP/6-31G*	-2.26
E	MN15/6-311++G**//B3LYP/6-31G*	-1.92
F	MN15/6-311++G**^{NiPC} 6-311G**^{NCH}//B3LYP/6-31G*	-1.94
G	MN15/ma-def2-TVPZ^{NiPC}def2-TVPZ^{NCH}//B3LYP/6-31G*	-1.93
H	M06L/def2-TZVP^{Ni} def2-SVP^{HCNP}	-1.99
I	M06L/6-311++G**//M06L/def2-TZVP^{Ni} def2-SVP^{HCNP}	-1.93
J	M06L/ma-def2-TVPZ^{NiPC}def2-TVPZ^{NCH}//M06L/def2-TZVP^{Ni} def2-SVP^{HCNP}	-2.00
K	MN15/6-311++G**^{NiPC} 6-311G**^{NCH}//M06L/def2-TZVP^{Ni} def2-SVP^{HCNP}	-1.87
L	MN15/ma-def2-TVPZ^{NiPC} def2-TVPZ^{NCH}//M06L/def2-TZVP^{Ni} def2-SVP^{HCNP}	-1.86
M	wB97XD/ma-def2-TVPZ^{NiPC} def2-TVPZ^{NCH}//M06L/def2-TZVP^{Ni} def2-SVP^{HCNP}	-2.31
N	M06L/ma-def2-TZVP^{Ni} ma-def2-SVP^{CP} def2-SVP^{NCH}	-2.01
O	M06L/ma-def2-TVPZ^{NiPC} def2-TVPZ^{NCH}//M06L/ma-def2-TZVP^{Ni} ma-def2-SVP^{CP} def2-SVP^{NCH}//	-2.03

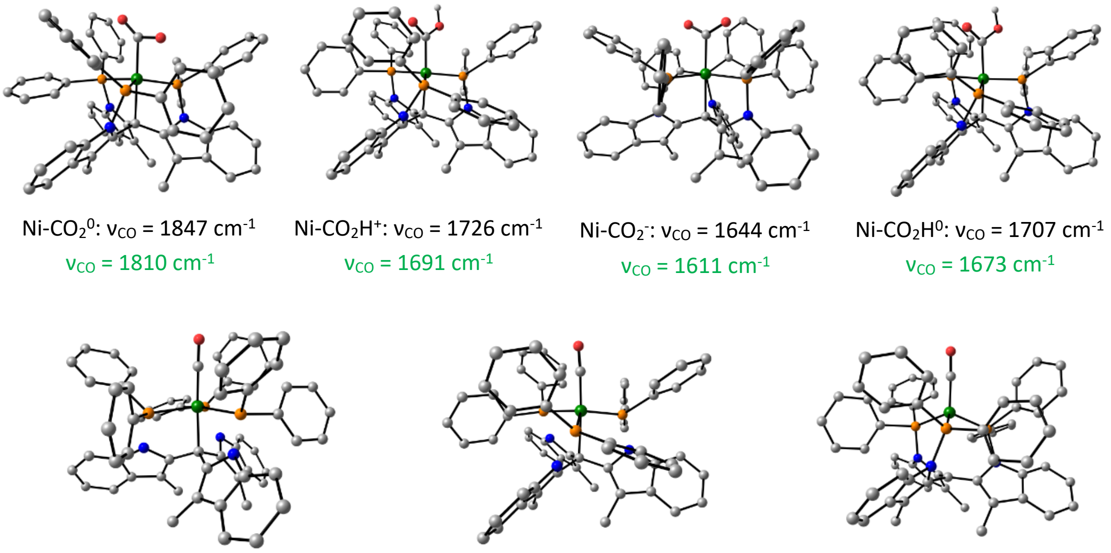

Ni-CO_2^0: ν_{CO} = 1847 cm^{-1} — ν_{CO} = 1810 cm^{-1}

Ni-CO_2H^+: ν_{CO} = 1726 cm^{-1} — ν_{CO} = 1691 cm^{-1}

Ni-CO_2^-: ν_{CO} = 1644 cm^{-1} — ν_{CO} = 1611 cm^{-1}

Ni-CO_2H^0: ν_{CO} = 1707 cm^{-1} — ν_{CO} = 1673 cm^{-1}

Ni-CO^+: ν_{CO} = 2103 cm^{-1} — ν_{CO} = 2061 cm^{-1}

Ni-CO^0: ν_{CO} = 2029 cm^{-1} — ν_{CO} = 1988 cm^{-1}

Ni-CO^-: ν_{CO} = 1963 cm^{-1} — ν_{CO} = 1924 cm^{-1}

Figure S 26. DFT structures of possible Ni-CO_2 and Ni-CO intermediates optimized at the B3LYP-D_3/6-31G* level of theory. An empirical factor of 0.98 has been used to correct the DFT values (shown in green). This correction factor is the ratio between the experimental Ni-CO^+ ν_{CO} (2060 cm^{-1}) and its DFT-calculated value (2103 cm^{-1}).

5.3. DFT xyz coordinates

The charge and spin state of each geometry are indicated at the beginning of each set of xyz coordinates as q,s where q = charge and s = spin multiplicity. The associated single point electronic energy value (E) calculated at the MN15/6-311++G** is given for each geometry.

CO2_s_q0_MN15_6-311++gdp
E = -188.4632401
0,1
C +1.85866 -4.27262
O +2.41050 -0.12683 -3.24971
O +1.30682 +0.12683 -5.29553

CO_s_q0_MN15_6-311++gdp
E = -113.230904226
0,1
C -0.14228 +0.04761 -2.62203
O -0.27543 +0.12348 -3.74932

H2O-2_HCO3_s_q-1_MN15_6-311++gdp
E = -417.1969552
-1,1
H +0.88941 -0.00819 -4.15893
O +0.76417 +0.73819 -3.54701
O +0.93011 -1.07721 -2.23044
C +0.77472 +0.16784 -2.27305
O +0.62276 +1.00276 -1.34861
O +0.87955 -2.33830 +0.28289
H +0.91313 -1.95771 -0.62399
H +0.74365 -1.52458 +0.81043
O +0.68810 +0.32082 +1.25387
H +0.62994 +0.54408 +0.28001
H +1.63737 +0.41501 +1.43855

H2O-3_HCO3_s_q-1_MN15_6-311++gdp
E = -493.5979092
-1,1
H +1.06978 +0.41564 -4.01345
O +1.07523 +1.08742 -3.30873
O +0.89184 -0.85986 -2.19525

C   +0.96821   +0.38284   -2.11338
O   +0.96238   +1.12802   -1.09283
O   +0.43149   -2.32417   +0.18823
H   +0.58575   -1.88195   -0.67461
H   +0.55952   -1.57246   +0.80239
O   +1.03836   +0.15378   +1.44023
H   +0.97418   +0.50294   +0.51055
H   +1.99080   -0.00847   +1.54496
H   +1.21870   +2.88137   -1.55324
O   +1.37039   +3.74793   -1.99497
H   +1.39141   +3.46272   -2.92190

H2O-3_OH_s_q-1_MN15_6-311++gdp
E = -305.0847944
-1,1
O   -1.75198   -0.47204   -5.56006
H   -1.36820   -1.24172   -5.08065
H   -0.92919   -0.05463   -5.90339
H   +1.12751   -0.49396   -6.53779
O   +0.87364   +0.37175   -6.17777
H   +0.92519   +0.21075   -5.13001
H   +0.34160   -2.62797   -5.05020
O   -0.15808   -2.39905   -4.24915
H   -0.12245   +0.27385   -3.62057
O   +0.76932   -0.10725   -3.69043
H   +0.29279   -1.48601   -3.94789

H2O-3_s_q0_MN15_6-311++gdp
E = -229.1797352
0,1
O   -1.26740   -0.64189   -2.50605
H   -0.71233   -0.18680   -1.84624
H   -3.41645   -1.56563   -0.33873
H   -0.08907   -1.36083   -3.82019
O   +0.55685   -1.65907   -4.49100
H   +1.06196   -0.85229   -4.68115
H   -1.78818   +0.07373   -2.91520
O   -3.09962   -2.24186   -0.95891
H   -2.47048   -1.74681   -1.52039

H2O-4_q0_1_MN15
E = -305.5820357
0,1
O   -2.18442   +1.50972   -4.78144
H   -1.90196   +0.96773   -4.02317
H   -1.62755   +1.15947   -5.50986
H   -1.13426   -0.44312   -7.03110
O   -0.52421   +0.24291   -6.71127
H   +0.02190   -0.23121   -6.03425
H   -1.57983   -0.96933   -2.39887
O   -1.02230   -0.25241   -2.75229
H   -0.65679   +0.18943   -1.96445
H   +1.62886   -0.49690   -4.49170
O   +0.83351   -1.02422   -4.68059
H   +0.22241   -0.80925   -3.93895

H2O_s_q0_MN15_6-311++gdp
E = -76.3894152
0,1
O   -0.35655   -0.13807   +0.09009
H   -0.35655   +0.62186   +0.69553
H   -0.35655   -0.89801   +0.69553

MeCN_s_q0_MN15_6-311++gdp
E = -132.6410211
0,1
C   -1.57927   -5.2e-05   +1.00406
N   -1.57950   +4.8e-05   -0.15714
C   -1.57932   -1e-05   +2.46203
H   -0.55027   +1e-05   +2.83509
H   -2.09401   -0.89132   +2.83509
H   -2.09401   +0.89132   +2.83504

Ni-TIM_CO-OH_q-1_d_3H2O_CO2_1-7_R_MN15
E = -5770.762508
-1,2
Ni   +0.00581   -0.01247   -0.65845
P   -2.12511   +0.72825   -0.88978
P   +0.20885   -2.18502   +0.09277
P   +1.73948   +1.39295   -0.14020
C   -2.63099   -0.58193   +2.74847
N   -2.79281   +0.26863   +0.64302
N   +0.71374   -1.86173   +1.73495
N   +0.98339   +2.30654   +1.14020
C   -0.46933   +0.33055   +1.54837
C   -1.90253   -0.03823   +1.71286
C   -4.01375   -0.59678   +2.34263
C   -5.18922   -1.01134   +2.99059
H   -5.14350   -1.42785   +3.99372
C   -6.40905   -0.87480   +2.33116
H   -7.32611   -1.19101   +2.82184
C   -6.47006   -0.32906   +1.03454
H   -7.43144   -0.23305   +0.53678
C   -5.31662   +0.09295   +0.37109

H	-5.37448	+0.50397	-0.62888
C	-4.09541	-0.04269	+1.03683
C	-2.17762	-0.99828	+4.11470
H	-2.68178	-1.92307	+4.42313
H	-2.42595	-0.23592	+4.86762
H	-1.10172	-1.16948	+4.16019
C	-3.03563	-0.25569	-2.13014
C	-3.46714	-1.55613	-1.82910
H	-3.39880	-1.92818	-0.81381
C	-3.96949	-2.38136	-2.83534
H	-4.30261	-3.38411	-2.58482
C	-4.02669	-1.92890	-4.15658
H	-4.41093	-2.57726	-4.93932
C	-3.58062	-0.64137	-4.46694
H	-3.61463	-0.28324	-5.49229
C	-3.08667	+0.19196	-3.46158
H	-2.74061	+1.18888	-3.71741
C	-2.69146	+2.45892	-1.14089
C	-3.95192	+2.79857	-1.66211
H	-4.63274	+2.02520	-2.00066
C	-4.33482	+4.13624	-1.76656
H	-5.31211	+4.38471	-2.17171
C	-3.46836	+5.15222	-1.35042
H	-3.77143	+6.19271	-1.43106
C	-2.21101	+4.82552	-0.83925
H	-1.52627	+5.60537	-0.51762
C	-1.82491	+3.48780	-0.74131
H	-0.84482	+3.24450	-0.35707
C	+0.48398	-0.55760	+2.26086
C	+1.21157	-0.41001	+3.42347
C	+2.76973	-2.07118	+4.67927
H	+3.03200	-1.39246	+5.48694
C	+1.91279	-1.64613	+3.65079
C	+3.26437	-3.37343	+4.65302
H	+3.92979	-3.71537	+5.44182
C	+1.21256	+0.72447	+4.40306
H	+2.21853	+0.88316	+4.81076
H	+0.55197	+0.51366	+5.25697
H	+0.87785	+1.66135	+3.95590
C	+2.04147	-3.86008	+2.59334
H	+1.75007	-4.55972	+1.82252
C	+2.22594	+2.62554	-1.41767
C	+1.57083	-2.54456	+2.60277
C	-1.15751	-3.38635	+0.32252
C	-1.65776	-4.07197	-0.79795
H	-1.20643	-3.91589	-1.77480
C	-2.74166	-4.94011	-0.66033
H	-3.11938	-5.46857	-1.53172
C	-3.34706	-5.12253	+0.58694
H	-4.19734	-5.79175	+0.68836
C	+1.52469	-3.20006	-0.68836
C	+2.87490	+4.37484	-3.52295
H	+3.12742	+5.04744	-4.33814
C	-1.75574	-3.58685	+1.57429
H	-1.36842	-3.07351	+2.44618
C	+2.49035	-2.51028	-1.43389
H	+2.41738	-1.43492	-1.53114
C	+3.51520	-3.19526	-2.08933
H	+4.24274	-2.64238	-2.67517
C	-0.20262	+1.77626	+1.72116
C	+3.56873	-4.58872	-2.01871
H	+4.35713	-5.12955	-2.53611
C	+1.57411	-4.60378	-0.64538
H	+0.81652	-5.16362	-0.10666
C	+0.71969	+6.26052	+2.38128
H	+0.59616	+7.28416	+2.72602
C	-0.26128	+5.31297	+2.66880
H	-1.14354	+5.58953	+3.24057
C	+2.59328	-5.29156	-1.30381
H	+2.62290	-6.37724	-1.26096
C	+1.87602	+5.90748	+1.66034
H	+2.63570	+6.65825	+1.45949
C	-0.09232	+3.99219	+2.21782
C	-0.87825	+2.79184	+2.36928
C	+2.07070	+4.60210	+1.20435
H	+2.97081	+4.33229	+0.66568
C	+1.07279	+3.66189	+1.47029
C	+2.89746	-4.25868	+3.62228
H	+3.27807	-5.27677	+3.62701
C	+1.56165	+3.92403	-3.36572
H	+0.78557	+4.24346	-4.05590
C	-2.14746	+2.69802	+3.16743
H	-2.40989	+3.67623	+3.58395
H	-2.05331	+1.99848	+4.00670
H	-2.99617	+2.36153	+2.56083
C	+3.86386	+3.94892	-2.63249
H	+4.88842	+4.29154	-2.74946
C	-2.85007	-4.44516	+1.70322
H	-3.30972	-4.58555	+2.67816
C	+1.24246	+3.04767	-2.32820
H	+0.22590	+2.68470	-2.23243
C	+3.54294	+3.08243	-1.58601
H	+4.32309	+2.76502	-0.90244
C	+3.33377	+0.87602	+0.60869
C	+4.82140	+0.67794	+2.51712
H	+5.04366	+0.90054	+3.55746
C	+5.70817	-0.08890	+1.75748
H	+6.62157	-0.47103	+2.20561
C	+5.41921	-0.35498	+0.41575
H	+6.10934	-0.93794	-0.18875
C	+3.64253	+1.16258	+1.94595
H	+2.96238	+1.75929	+2.54194
C	+4.24134	+0.12489	-0.15816
H	+4.03502	-0.06823	-1.20518
C	+0.20579	-0.07191	-2.46269
O	+3.15128	+0.13417	-3.26300

O +0.05847 +0.19134 -3.58091
H +2.85774 +1.03128 -3.49004
O +1.81545 -1.59527 -4.95808
H +2.64474 -0.46998 -3.85744
H -1.09303 -2.21355 -3.74373
H +4.84602 -1.83706 -4.63605
O +5.44892 -1.24046 -4.13632
H +4.81524 -0.58299 -3.78577
H +1.11477 -1.15578 -5.47385
O -0.45590 -2.94441 -3.69737
H +0.39524 -2.54242 -3.96329
C +2.55883 -2.40597 -5.93070
O +3.60279 -2.89006 -5.45631
O +2.02396 -2.47747 -7.04229

Ni-TIM_CO-OH_q-1_d_3H2O_CO2_2-3_TS_MN15
E = -5770.73519395
-1,2
Ni +0.05758 +0.02208 -0.66724
P -2.09776 +0.69808 -0.90312
P +0.31015 -2.14552 +0.06825
P +1.77443 +1.42759 -0.10337
C -2.59978 -0.60793 +2.73252
N -2.76777 +0.26831 +0.63687
N +0.75789 -1.83957 +1.73591
N +0.98877 +2.33099 +1.16780
C -0.44954 +0.33965 +1.54524
C -1.87637 -0.04720 +1.70119
C -3.98486 -0.61374 +2.33217
C -5.16308 -1.02929 +2.97602
H -5.12015 -1.45908 +3.97358
C -6.38354 -0.87568 +2.32174
H -7.30096 -1.19252 +2.81140
C -6.44600 -0.31062 +1.03334
H -7.40844 -0.19983 +0.54074
C -5.29129 +0.11223 +0.37324
H -5.34715 +0.54130 -0.61933
C -4.06955 -0.04252 +1.03317
C -2.12734 -1.03929 +4.08914
H -2.79889 -1.80014 +4.50402
H -2.11177 -0.20130 +4.80176
H -1.12020 -1.45998 +4.06434
C -2.99874 -0.31418 -2.12958
C -3.49537 -1.58521 -1.80575
H -3.46608 -1.93743 -0.78140
C -4.01778 -2.41062 -2.80263
H -4.40522 -3.38877 -2.53408
C -4.02519 -1.99287 -4.13546
H -4.42566 -2.64233 -4.90912
C -3.50798 -0.73786 -4.46971
H -3.50215 -0.40599 -5.50443
C -2.99930 +0.09811 -3.47465
H -2.60408 +1.07160 -3.74848
C -2.68192 +2.41838 -1.19294
C -3.92262 +2.73866 -1.77015
H -4.58111 +1.95416 -2.12753
C -4.31425 +4.07106 -1.90731
H -5.27592 +4.30403 -2.35672
C -3.47632 +5.10125 -1.46854
H -3.78574 +6.13749 -1.57554
C -2.23835 +4.79378 -0.90085
H -1.57513 +5.58461 -0.56125
C -1.84328 +3.46146 -0.77042
H -0.87746 +3.23347 -0.34227
C +0.50316 -0.54061 +2.26548
C +1.19860 -0.39200 +3.44663
C +2.73677 -2.04233 +4.73635
H +2.96921 -1.36371 +5.55324
C +1.90464 -1.62189 +3.68580
C +3.24502 -3.33929 +4.71996
H +3.89146 -3.67790 +5.52584
C +1.16981 +0.74131 +4.42682
H +2.16518 +0.90472 +4.85827
H +0.49062 +0.52793 +5.26532
H +0.84198 +1.67701 +3.97207
C +2.08426 -3.82870 +2.62498
H +1.82315 -4.52951 +1.84541
C +2.29633 +2.68226 -1.34400
C +1.59880 -2.51835 +2.62401
C -1.05727 -3.35832 +0.23280
C -1.50900 -4.02305 -0.92055
H -1.03350 -3.82490 -1.87904
C -2.57703 -4.91740 -0.83705
H -2.91765 -5.43083 -1.73247
C -3.21779 -5.14307 +0.38528
H -4.05594 -5.83262 +0.44339
C +1.64270 -3.16095 -0.68462
C +2.98487 +4.51156 -3.36632
H +3.25235 +5.21731 -4.14808
C -1.69134 -3.59960 +1.45913
H -1.34095 -3.10191 +2.35529
C +2.56675 -2.48289 -1.48828
H +2.46970 -1.41480 -1.62577
C +3.56956 -3.17474 -2.17001
H +4.22519 -2.63764 -2.84971
C -0.20337 +1.78771 +1.72339
C +3.65558 -4.56216 -2.04939
H +4.42548 -5.10984 -2.58710
C +1.71442 -4.56258 -0.60172
H +0.97535 -5.11726 -0.03264
C +0.65442 +6.28328 +2.39821
H +0.51204 +7.30569 +2.73939
C -0.32177 +5.32488 +2.66580

H -1.21877 +5.59204 +3.21882
C +2.72461 -5.25551 -1.26759
H +2.77448 -6.33858 -1.18976
C +1.82935 +5.94286 +1.70183
H +2.58440 +6.70210 +1.51570
C -0.12882 +4.00564 +2.21977
C -0.90480 +2.79719 +2.35492
C +2.04818 +4.63917 +1.25154
H +2.96167 +4.37952 +0.73060
C +1.05595 +3.68760 +1.49794
C +2.91545 -4.22316 +3.67573
H +3.30529 -5.23774 +3.68661
C +1.66075 +4.08853 -3.22507
H +0.89095 +4.46203 -3.89492
C -2.19298 +2.69386 +3.12084
H -2.48611 +3.67515 +3.50886
H -2.10878 +2.01486 +3.97797
H -3.01803 +2.32855 +2.49878
C +3.96543 +4.01584 -2.50294
H +4.99838 +4.33668 -2.60808
C -2.77297 -4.48059 +1.53227
H -3.26162 -4.65221 +2.48796
C +1.32193 +3.17133 -2.22994
H +0.29618 +2.83229 -2.14555
C +3.62502 +3.10941 -1.49748
H +4.39758 +2.74248 -0.83030
C +3.34658 +0.89412 +0.67843
C +4.80193 +0.68990 +2.61016
H +5.01290 +0.91789 +3.65172
C +5.68516 -0.10066 +1.87083
H +6.58444 -0.49546 +2.33631
C +5.41147 -0.37418 +0.52734
H +6.10109 -0.97577 -0.05936
C +3.64000 +1.18887 +2.01721
H +2.96011 +1.80115 +2.59783
C +4.25107 +0.12092 -0.06989
H +4.05440 -0.07082 -1.12027
C +0.27673 +0.07980 -2.48015
O +3.25145 +0.10863 -3.18788
O +0.16905 +0.45803 -3.56735
H +2.84519 +0.97192 -3.35996
O +2.01021 -1.67120 -4.71314
H +2.69714 -0.56185 -3.71087
H -0.91125 -2.03610 -3.87669
H +3.76646 -1.76058 -5.03240
O +4.73706 -1.55047 -4.99310
H +4.69422 -0.77898 -4.39955
H +1.61985 -1.05850 -5.35837
O -0.23601 -2.69067 -3.63948
H +0.62717 -2.27821 -3.95164
C +1.62915 -3.51810 -5.90365
O +2.20539 -4.32133 -5.26117
O +0.99286 -3.03354 -6.77439

Ni-TIM_CO-OH_q-1_d_3H2O_CO2_2-7_P_MN15
E = -5770.7369758
-1,2
Ni +0.07506 +0.02040 -0.67688
P -2.08537 +0.67945 -0.91893
P +0.32909 -2.14567 +0.05863
P +1.78662 +1.43093 -0.10500
C -2.59252 -0.61125 +2.72128
N -2.75738 +0.25678 +0.62205
N +0.77221 -1.83940 +1.72822
N +0.99352 +2.33084 +1.16510
C -0.44157 +0.33608 +1.53523
C -1.86767 -0.05403 +1.68902
C -3.97701 -0.61825 +2.31918
C -5.15646 -1.02964 +2.96349
H -5.11514 -1.45546 +3.96283
C -6.37606 -0.87674 +2.30747
H -7.29441 -1.19024 +2.79757
C -6.43659 -0.31636 +1.01690
H -7.39849 -0.20581 +0.52317
C -5.28066 +0.10231 +0.35626
H -5.33487 +0.52829 -0.63779
C -4.05979 -0.05185 +1.01794
C -2.12176 -1.03747 +4.08010
H -2.79391 -1.79664 +4.49708
H -2.10679 -0.19690 +4.78973
H -1.11464 -1.45844 +4.05790
C -2.98145 -0.34145 -2.14226
C -3.48785 -1.60707 -1.81272
H -3.46801 -1.95222 -0.78581
C -4.00857 -2.43668 -2.80720
H -4.40403 -3.41028 -2.53396
C -4.00385 -2.02928 -4.14309
H -4.40277 -2.68221 -4.91465
C -3.47679 -0.77984 -4.48301
H -3.46189 -0.45561 -5.52007
C -2.97090 +0.06070 -3.49058
H -2.56971 +1.03044 -3.76872
C -2.67616 +2.39609 -1.21883
C -3.91165 +2.70912 -1.81078
H -4.56282 +1.92046 -2.17252
C -4.30748 +4.03939 -1.95688
H -5.26498 +4.26660 -2.41801
C -3.47897 +5.07467 -1.51233
H -3.79145 +6.10923 -1.62657
C -2.24632 +4.77437 -0.92931
H -1.59037 +5.56920 -0.58492
C -1.84697 +3.44422 -0.79002
H -0.88513 +3.22183 -0.34990
C +0.51200 -0.54142 +2.25746

C +1.20420 -0.39054 +3.44027
C +2.74540 -2.03522 +4.73314
H +2.97368 -1.35564 +5.55042
C +1.91385 -1.61791 +3.68088
C +3.25866 -3.33021 +4.71788
H +3.90475 -3.66633 +5.52508
C +1.17001 +0.74316 +4.41981
H +2.16430 +0.91063 +4.85222
H +0.49072 +0.52789 +5.25776
H +0.83920 +1.67731 +3.96403
C +2.10416 -3.82384 +2.62039
H +1.84775 -4.52555 +1.84012
C +2.30984 +2.69411 -1.33639
C +1.61348 -2.51540 +2.61823
C -1.04153 -3.35577 +0.22064
C -1.48870 -4.02304 -0.93306
H -1.01054 -3.82462 -1.89043
C -2.55413 -4.92058 -0.85075
H -2.89086 -5.43664 -1.74613
C -3.19765 -5.14600 +0.37017
H -4.03382 -5.83806 +0.42722
C +1.65993 -3.17035 -0.68578
C +2.99865 +4.54237 -3.34110
H +3.26620 +5.25578 -4.11587
C -1.67836 -3.59657 +1.44555
H -1.33139 -3.09685 +2.34189
C +2.59114 -2.50198 -1.48838
H +2.50260 -1.43419 -1.63540
C +3.59265 -3.20537 -2.16206
H +4.25617 -2.67455 -2.83913
C -0.20034 +1.78478 +1.71398
C +3.66560 -4.59343 -2.03889
H +4.43470 -5.14905 -2.57004
C +1.72004 -4.57278 -0.59828
H +0.97553 -5.11928 -0.02847
C +0.64188 +6.28293 +2.39220
H +0.49478 +7.30513 +2.73203
C -0.33368 +5.32223 +2.65384
H -1.23476 +5.58745 +3.20114
C +2.72483 -5.27703 -1.25937
H +2.76470 -6.36027 -1.17842
C +1.82212 +5.94504 +1.70365
H +2.57664 +6.70595 +1.52219
C -0.13474 +4.00324 +2.20954
C -0.90839 +2.79299 +2.34067
C +2.04689 +4.64166 +1.25530
H +2.96439 +4.38409 +0.74047
C +1.05530 +3.68773 +1.49525
C +2.93464 -4.21520 +3.67288
H +3.32844 -5.22826 +3.68438
C +1.67443 +4.11841 -3.20366
H +0.90459 +4.49865 -3.86970
C -2.20167 +2.68829 +3.09785
H -2.49952 +3.66983 +3.48167
H -2.12230 +2.01139 +3.95705
H -3.02165 +2.32019 +2.47076
C +3.97928 +4.03765 -2.48296
H +5.01238 +4.35891 -2.58543
C -2.75823 -4.47991 +1.51716
H -3.24919 -4.65106 +2.47177
C +1.33557 +3.19178 -2.21734
H +0.30992 +2.85183 -2.13617
C +3.63868 +3.12159 -1.48631
H +4.41114 +2.74734 -0.82307
C +3.35642 +0.89888 +0.68291
C +4.80657 +0.69937 +2.61897
H +5.01522 +0.93031 +3.66036
C +5.69072 -0.09453 +1.88425
H +6.58839 -0.48890 +2.35325
C +5.42003 -0.37178 +0.54089
H +6.11083 -0.97578 -0.04205
C +3.64663 +1.19762 +2.02153
H +2.96559 +1.81192 +2.59868
C +4.26170 +0.12250 -0.06132
H +4.06650 -0.06919 -1.11257
C +0.29895 +0.09384 -2.49209
O +3.28497 +0.12552 -3.14987
O +0.17964 +0.48429 -3.57316
H +2.88948 +0.99296 -3.32330
O +2.06458 -1.65005 -4.59129
H +2.71894 -0.55174 -3.68553
H -0.82925 -2.05002 -3.90850
H +3.74942 -1.79486 -4.95171
O +4.73310 -1.61037 -4.94316
H +4.71943 -0.82515 -4.36640
H +1.68314 -1.09645 -5.29203
O -0.16285 -2.69843 -3.63224
H +0.72048 -2.27488 -3.90967
C +1.05116 -3.72957 -5.98362
O +1.62353 -4.57119 -5.40706
O +0.45481 -2.98968 -6.66910

Ni-TIM_CO-OH_q-1_d_3H2O_MN15_6-311++Gdp_P
E = -5582.2578168
-1,2
Ni -0.01166 +0.04138 -2.64791
P -2.10664 +0.36392 -1.94062
P +0.66494 -1.97653 -1.99133
P +1.40045 +1.62042 -1.94366
C -1.73352 -1.55732 +1.45156
N -2.39920 -0.24922 -0.31035
N +1.35089 -1.97446 -0.36327
N +0.99634 +2.18285 -0.30141

C	+0.00147	-0.01678	+0.33608
C	-1.29585	-0.66747	+0.48569
C	-3.16046	-1.66006	+1.31203
C	-4.13155	-2.36444	+2.04424
H	-3.83424	-3.00302	+2.87217
C	-5.47126	-2.22115	+1.69581
H	-6.23565	-2.75722	+2.25226
C	-5.85028	-1.37883	+0.63210
H	-6.90240	-1.27285	+0.38134
C	-4.90340	-0.66945	-0.10710
H	-5.20948	-0.02477	-0.92027
C	-3.55690	-0.81964	+0.23702
C	-0.94745	-2.20371	+2.54905
H	-1.40673	-2.00734	+3.52672
H	+0.08344	-1.84634	+2.57856
H	-0.91998	-3.29472	+2.42463
C	-3.22268	-0.59815	-3.04222
C	-3.69324	-1.88517	-2.75951
H	-3.46530	-2.35728	-1.81252
C	-4.45258	-2.58226	-3.70404
H	-4.81533	-3.57695	-3.46248
C	-4.72317	-2.01938	-4.95074
H	-5.30906	-2.56757	-5.68356
C	-4.22346	-0.74870	-5.25718
H	-4.41544	-0.30363	-6.22981
C	-3.48001	-0.04463	-4.31183
H	-3.10154	+0.94204	-4.55979
C	-2.96429	+2.00494	-1.86218
C	-4.31744	+2.20286	-2.18477
H	-4.91931	+1.38184	-2.55849
C	-4.90576	+3.46103	-2.04309
H	-5.95363	+3.59370	-2.29904
C	-4.15492	+4.54295	-1.57441
H	-4.61648	+5.52064	-1.46437
C	-2.80765	+4.36087	-1.25698
H	-2.20619	+5.19195	-0.89898
C	-2.22166	+3.10340	-1.40650
H	-1.17384	+2.98047	-1.18281
C	+1.20646	-0.82284	+0.45116
C	+2.22209	-0.78751	+1.39259
C	+4.10539	-2.51130	+1.90110
H	+4.54636	-1.96206	+2.72905
C	+3.00241	-1.98289	+1.20900
C	+4.61091	-3.74969	+1.51423
H	+5.46317	-4.17344	+2.03908
C	+2.41241	+0.18994	+2.50964
H	+3.40040	+0.66546	+2.45485
H	+2.35536	-0.31124	+3.48523
H	+1.65813	+0.97963	+2.48916
C	+2.92495	-3.96589	-0.24403
H	+2.47302	-4.52836	-1.05280
C	+1.32948	+3.12585	-3.02839
C	+2.42793	-2.71633	+0.13592

C	-0.50268	-3.39081	-1.89034
C	-1.20474	-3.77533	-3.04383
H	-1.07993	-3.23247	-3.98196
C	-2.08393	-4.86004	-2.99593
H	-2.61558	-5.15360	-3.89706
C	-2.30686	-5.54064	-1.79708
H	-3.00567	-6.37245	-1.76159
C	+2.07494	-2.65273	-2.97884
C	+1.12115	+5.30313	-4.80815
H	+1.04222	+6.14205	-5.49450
C	-0.72303	-4.08159	-0.68845
H	-0.18797	-3.79343	+0.20878
C	+3.33478	-2.06049	-2.80088
H	+3.46418	-1.28886	-2.05262
C	+4.42761	-2.46462	-3.56522
H	+5.39171	-1.98878	-3.41051
C	+0.07556	+1.42372	+0.47286
C	+4.27936	-3.46870	-4.52592
H	+5.12757	-3.77803	-5.13048
C	+1.93765	-3.67181	-3.93174
H	+0.98414	-4.16224	-4.08490
C	+0.47988	+5.90690	+1.49939
H	+0.30317	+6.85781	+1.99516
C	-0.26897	+4.78968	+1.85783
H	-1.02650	+4.85305	+2.63453
C	+3.03320	-4.07256	-4.70124
H	+2.90654	-4.85875	-5.44088
C	+1.48136	+5.81328	+0.51356
H	+2.07402	+6.68995	+0.26602
C	-0.02506	+3.56846	+1.20583
C	-0.57837	+2.25281	+1.37204
C	+1.74180	+4.61125	-0.14618
H	+2.53547	+4.54759	-0.87761
C	+0.96689	+3.49775	+0.18698
C	+4.02218	-4.46954	+0.45554
H	+4.42449	-5.44052	+0.17917
C	+0.00756	+4.50285	-4.54390
H	-0.94507	+4.71086	-5.02388
C	-1.6135	+1.86041	+2.38062
H	-1.68293	+2.60608	+3.17957
H	-1.38296	+0.89335	+2.83731
H	-2.60804	+1.77195	+1.92265
C	+2.34064	+5.01117	-4.19227
H	+3.21615	+5.62196	-4.39599
C	-1.63003	-5.14181	-0.64052
H	-1.79999	-5.66034	+0.29965
C	+0.11722	+3.41802	-3.67381
H	-0.74374	+2.78497	-3.50377
C	+2.44401	+3.93416	-3.31050
H	+3.40175	+3.72857	-2.84508
C	+3.21434	+1.38275	-1.79355
C	+5.25893	+1.25169	-0.49127
H	+5.76281	+1.31797	+0.46961

C +5.98493 +0.95512 -1.64691
H +7.05712 +0.78620 -1.59028
C +5.32513 +0.87669 -2.87770
H +5.88229 +0.64213 -3.78099
C +3.88146 +1.47571 -0.56520
H +3.32815 +1.72698 +0.33195
C +3.94662 +1.07763 -2.95383
H +3.44120 +0.96421 -3.91172
C -0.19230 +0.27159 -4.37154
O +2.58518 +0.29820 -5.84073
O -0.46649 +0.62171 -5.44910
H +2.02765 +1.08578 -5.93442
O +1.21799 -1.58270 -6.97435
H +1.98007 -0.46506 -6.19617
H -1.52992 -1.60271 -5.81175
H +2.80893 -1.75938 -7.58363
O +3.76682 -1.56240 -7.80175
H +3.86356 -0.74750 -7.27971
H +0.83287 -1.01385 -7.66155
O -1.06371 -2.43568 -5.97486
H -0.15246 -2.13269 -6.32145

Ni-TIM_CO2H2_q1_s_MN15_6-311++Gdp
E = -5353.4318884
1,1
Ni -0.02699 -0.01438 -0.79863
P -2.14977 +0.82309 -0.61966
P +0.38085 -2.20037 -0.57373
P +1.75590 +1.41791 -0.50050
C -1.96793 -1.29375 +2.62845
N -2.55810 +0.00296 +0.84889
N +1.05945 -2.1909 +1.00388
N +1.22285 +2.05119 +1.03171
C -0.10602 -0.03493 +1.24457
C -1.48333 -0.50938 +1.60899
C -3.40968 -1.24899 +2.53110
C -4.43172 -1.83353 +3.29842
H -4.18868 -2.45723 +4.15423
C -5.75739 -1.59792 +2.94811
H -6.55687 -2.04282 +3.53439
C -6.07910 -0.78867 +1.84059
H -7.12141 -0.62132 +1.58336
C -5.08588 -0.19516 +1.06428
H -5.34189 +0.41610 +0.20838
C -3.75539 -0.42846 +1.42463
C -1.21993 -2.00330 +3.71897
H -1.91237 -2.57909 +4.34053
H -0.69590 -1.29858 +4.37517
H -0.47158 -2.69875 +3.32907
C -3.30134 +0.19651 -1.88539
C -3.81839 -1.10326 -1.80700
H -3.60814 -1.72223 -0.94392
C -4.60850 -1.60489 -2.84177
H -5.01072 -2.61080 -2.76565
C -4.87151 -0.82251 -3.96854
H -5.48566 -1.21629 -4.77366
C -4.33924 +0.46673 -4.06044
H -4.53566 +1.07819 -4.93668
C -3.55613 +0.97777 -3.02512
H -3.15329 +1.98335 -3.10399
C -2.62533 +2.57820 -0.39845
C -3.97215 +2.98163 -0.43683
H -4.75532 +2.26456 -0.65469
C -4.31117 +4.31528 -0.21246
H -5.35488 +4.61545 -0.24230
C -3.31396 +5.26182 +0.04418
H -3.58239 +6.30054 +0.21651
C -1.97442 +4.87089 +0.07205
H -1.19067 +5.59765 +0.26512
C -1.63321 +3.53588 -0.14789
H -0.59347 +3.24784 -0.12767
C +0.94973 -0.98602 +1.73463
C +1.79478 -1.02810 +2.81564
C +3.40546 -2.92228 +3.61241
H +3.77455 -2.40064 +4.49126
C +2.45788 -2.31532 +2.77345
C +3.85574 -4.20294 +3.29925
H +4.58959 -4.68653 +3.93835
C +1.99274 -0.04828 +3.92968
H +3.05990 +0.06180 +4.15686
H +1.50571 -0.40111 +4.84897
H +1.58961 +0.93676 +3.69705
C +2.42276 -4.30784 +1.32152
H +2.04282 -4.83547 +0.45330
C +1.88279 +2.80670 -1.69439
C +1.98087 -3.02303 +1.64218
C -0.97597 -3.41298 -0.54408
C -1.77525 -3.56881 -1.68753
H -1.59304 -2.96508 -2.57217
C -2.82856 -4.48368 -1.68400
H -3.43900 -4.60132 -2.57434
C -3.11078 -5.22295 -0.53242
H -3.94044 -5.92445 -0.52688
C +1.69949 -2.88631 -1.63694
C +2.00212 +4.87851 -3.58823
H +2.04952 +5.67948 -4.32086
C -1.25972 -4.15828 +0.61022
H -0.65210 -4.04166 +1.50000
C +3.00926 -2.42638 -1.41986
H +3.21879 -1.74500 -0.60412
C +4.04700 -2.84991 -2.24946
H +5.05460 -2.48778 -2.06962
C +0.14616 +1.38298 +1.66065
C +3.78632 -3.72562 -3.30769

H +4.59445 -4.05025 -3.95706
C +1.44210 -3.76978 -2.69481
H +0.44065 -4.14496 -2.87399
C +1.17292 +5.62597 +3.13451
H +1.11339 +6.54444 +3.71213
C +0.25937 +4.60229 +3.37024
H -0.50977 +4.71052 +4.13003
C +2.48526 -4.18290 -3.52652
H +2.27697 -4.86623 -4.34490
C +2.18313 +5.48405 +2.16298
H +2.89325 +6.29137 +2.00627
C +0.35056 +3.42206 +2.61432
C -0.41543 +2.19495 +2.61595
C +2.29858 +4.32149 +1.40187
H +3.08885 +4.21261 +0.67016
C +1.36842 +3.30525 +1.63057
C +3.36901 -4.88667 +2.16753
H +3.73373 -5.88636 +1.94829
C +0.82299 +4.14819 -3.42457
H -0.05249 +4.37569 -4.02602
C -1.56422 +1.90652 +3.53722
H -1.74565 +2.76030 +4.19713
H -1.37258 +1.03257 +4.16839
H -2.49021 +1.71553 +2.98320
C +3.12350 +4.57113 -2.81331
H +4.04572 +5.13111 -2.93974
C -2.33035 -5.05331 +0.61428
H -2.55070 -5.62014 +1.51453
C +0.76631 +3.11366 -2.49083
H -0.15630 +2.55703 -2.37628
C +3.06733 +3.54229 -1.87321
H +3.94909 +3.31277 -1.28493
C +3.49272 +0.94704 -0.17387
C +5.28281 +0.45899 +1.39087
H +5.64411 +0.41563 +2.41462
C +6.11710 +0.09233 +0.33330
H +7.13183 -0.24294 +0.52930
C +5.64427 +0.16579 -0.98034
H +6.28893 -0.10978 -1.81014
C +3.97871 +0.89032 +1.14053
H +3.34663 +1.19105 +1.96602
C +4.33979 +0.58709 -1.23617
H +3.98671 +0.61420 -2.26188
C -0.16016 -0.11968 -2.57242
O +2.72655 -0.19586 -4.12556
O -0.36159 -0.21880 -3.69841
H +2.26003 +0.59629 -3.80825
H +2.60903 -0.83715 -3.40403

Ni-TIM_CO2H_q-1_d_MN15_6-311++gdp_1neg
E = -5353.0696905
-1,2
Ni -0.02164 +0.01841 -0.95808
P +0.29394 -2.15859 -0.62437
P +1.75725 +1.32787 -0.53812
P -2.10263 +0.77994 -0.61794
C +1.91582 -1.13321 +2.77558
N +1.00938 -2.21184 +0.97862
N +1.47529 +1.93276 +1.07555
N -2.47056 +0.26169 +1.02801
C -0.02809 +0.00901 +1.45062
C +0.98010 -1.0241 +1.76377
C +2.51273 -2.43909 +2.66894
C +3.47332 -3.12082 +3.43709
H +3.93751 -2.63420 +4.29161
C +3.81505 -4.42825 +3.09607
H +4.55654 -4.96226 +3.68551
C +3.20736 -5.07073 +2.00053
H +3.48473 -6.09220 +1.75318
C +2.24755 -4.41771 +1.22405
H +1.78316 -4.91753 +0.38324
C +1.91085 -3.10478 +1.56223
C +2.18214 -0.16063 +3.88704
H +3.10172 -0.42708 +4.42027
H +1.36875 -0.14749 +4.62789
H +2.29581 +0.86547 +3.52545
C +1.54063 -2.89251 -1.75174
C +2.91987 -2.85691 -1.50882
H +3.29904 -2.47385 -0.56938
C +3.82019 -3.30905 -2.47696
H +4.88556 -3.28097 -2.26638
C +3.35914 -3.77922 -3.70788
H +4.06268 -4.12895 -4.45876
C +1.98557 -3.78585 -3.97266
H +1.61522 -4.13700 -4.93239
C +1.08482 -3.34186 -3.00535
H +0.01960 -3.35178 -3.22257
C -0.96728 -3.51341 -0.48882
C -0.71962 -4.86248 -0.79413
H +0.24241 -5.16588 -1.19331
C -1.70728 -5.83081 -0.60231
H -1.49482 -6.86880 -0.84540
C -2.95981 -5.47029 -0.09698
H -3.72623 -6.22602 +0.05405
C -3.22200 -4.13182 +0.20280
H -4.19350 -3.83222 +0.58700
C -2.23614 -3.16506 +7.5e-05
H -2.46285 -2.12988 +0.20733
C +0.37503 +1.38193 +1.79291
C -0.02871 +2.28973 +2.75509
C +0.95491 +4.61767 +3.40884
H +0.25253 +4.82622 +4.21243
C +0.85271 +3.42697 +2.66946
C +1.97259 +5.52160 +3.10173

H +2.05897 +6.44672 +3.66671
C -1.06586 +2.12160 +3.82494
H -1.60105 +3.06347 +4.00210
H -0.61373 +1.82856 +4.78501
H -1.80387 +1.35946 +3.56840
C +2.82160 +4.06754 +1.33259
H +3.53802 +3.85372 +0.54649
C -3.44785 +0.08874 -1.68342
C +1.79495 +3.17193 +1.63455
C +3.48328 +0.71441 -0.46136
C +4.05216 +0.17608 -1.62884
H +3.46259 +0.14527 -2.54165
C +5.34896 -0.34014 -1.60677
H +5.78032 -0.74686 -2.51738
C +6.07814 -0.36275 -0.41468
H +7.08169 -0.77958 -0.39627
C +1.88212 +2.89238 -1.50649
C -5.35625 -1.07947 -3.39472
H -6.09588 -1.53242 -4.04990
C +4.22157 +0.69109 +0.73235
H +3.79067 +1.09425 +1.64257
C +0.79138 +3.77213 -1.41182
H -0.03223 +3.53334 -0.74729
C +0.76629 +4.95689 -2.14617
H -0.08315 +5.62705 -2.05102
C -1.41554 -0.35461 +1.76645
C +1.82548 +5.27432 -3.00237
H +1.80531 +6.19523 -3.57921
C +2.94039 +3.21777 -2.36490
H +3.80018 +2.56237 -2.45201
C -5.76478 -1.31261 +3.02160
H -6.59009 -1.76019 +3.57016
C -4.45441 -1.67923 +3.32647
H -4.25319 -2.40040 +4.11490
C +2.90917 +4.40009 -3.11015
H +3.73908 +4.63858 -3.77063
C -6.03295 -0.35524 +2.02570
H -7.05978 -0.06519 +1.81855
C -3.39379 -1.09543 +2.61090
C -1.96137 -1.1987 +2.71632
C -4.99589 +0.24125 +1.30349
H -5.20529 +1.00091 +0.56212
C -3.68709 -0.15615 +1.58062
C +2.89625 +5.25014 +2.07479
H +3.68471 +5.96584 +1.85671
C -4.13416 -1.71730 -3.16633
H -3.91204 -2.66584 -3.64862
C -1.22947 -2.00144 +3.75287
H -1.93718 -2.50304 +4.42191
H -0.58118 -1.37169 +4.37454
H -0.59255 -2.77576 +3.30802
C -5.61541 +0.15348 -2.78990
H -6.55598 +0.66609 -2.97445
C +5.50658 +0.14513 +0.75600
H +6.06382 +0.12402 +1.68922
C -3.18474 -1.12815 -2.33114
H -2.22110 -1.60328 -2.19214
C -4.66779 +0.73328 -1.94398
H -4.88243 +1.69766 -1.4952
C -2.53230 +2.56065 -0.56586
C -2.90584 +4.63450 +0.64292
H -3.03505 +5.15723 +1.58732
C -2.94567 +5.34128 -0.56184
H -3.10437 +6.41637 -0.56023
C -2.78000 +4.65725 -1.77119
H -2.80711 +5.20042 -2.71227
C -2.70658 +3.25162 +0.64198
H -2.68561 +2.70800 +1.58019
C -2.56485 +3.27930 -1.77480
H -2.40488 +2.75676 -2.71456
C +0.19278 +0.31717 -2.92408
O -0.92444 +0.71738 -3.67227
O +1.25249 +0.31788 -3.56137
H -0.58124 +0.91024 -4.57476

Ni-TIM_CO2H_q-1_d_MN15_6-311++gdp_isomer
E = -5353.0695887
-1,2
Ni -0.02768 +0.03869 -0.97923
P -0.93251 -1.96038 -0.62969
P +2.20248 +0.16451 -0.54935
P -1.34825 +1.79388 -0.60833
C +0.99326 -1.98880 +2.76973
N -0.35439 -2.39300 +0.97197
N +2.28540 +0.83183 +1.06558
N -1.93719 +1.56272 +1.03240
C -0.01917 +0.0327 +1.45390
C +0.27002 -1.38314 +1.75911
C +0.77519 -3.40782 +2.66325
C +1.19720 -4.50340 +3.43724
H +1.84613 -4.35008 +4.29618
C +0.76584 -5.78403 +3.09595
H +1.08664 -6.63647 +3.68992
C -0.0884 -5.98870 +1.99565
H -0.41599 -6.99507 +1.74794
C -0.52709 -4.91721 +1.21472
H -1.18527 -5.08092 +0.37055
C -0.09017 -3.63439 +1.55464
C +1.74901 -1.32881 +3.88580
H +2.43046 -2.04274 +4.36251
H +1.07862 -0.94769 +4.67112
H +2.34720 -0.48129 +3.54028
C -0.27714 -3.24550 -1.76181

C	+0.90019	-3.96371	-1.51653
H	+1.41978	-3.85440	-0.57244
C	+1.41946	-4.82288	-2.48852
H	+2.33045	-5.37557	-2.27698
C	+0.78423	-4.96056	-3.72390
H	+1.19156	-5.62949	-4.47734
C	-0.37437	-4.22295	-3.98957
H	-0.87122	-4.31234	-4.95219
C	-0.89721	-3.36850	-3.01960
H	-1.79737	-2.79928	-3.23838
C	-2.72225	-2.42758	-0.4848
C	-3.24642	-3.68874	-0.81402
H	-2.60566	-4.45446	-1.23813
C	-4.59858	-3.97359	-0.61170
H	-4.98452	-4.95535	-0.87400
C	-5.45039	-3.00609	-0.07075
H	-6.50157	-3.23162	+0.08866
C	-4.94330	-1.74618	+0.25480
H	-5.59279	-0.97933	+0.66883
C	-3.59424	-1.46134	+0.04098
H	-3.21922	-0.47408	+0.26654
C	+1.06313	+0.97097	+1.78360
C	+1.21746	+1.95990	+2.73923
C	+3.30668	+3.38337	+3.38448
H	+2.82800	+3.94715	+4.18173
C	+2.57480	+2.43527	+2.64946
C	+4.65369	+3.58399	+3.08089
H	+5.22858	+4.31662	+3.64239
C	+0.25851	+2.40085	+3.80445
H	+0.25175	+3.49527	+3.89622
H	+0.54166	+2.00545	+4.79172
H	-0.76339	+2.07505	+3.60272
C	+4.57814	+1.88864	+1.32425
H	+5.06537	+1.31378	+0.54417
C	-2.85679	+1.96135	-1.67587
C	+3.22763	+1.70038	+1.62143
C	+3.31485	-1.29187	-0.45026
C	+3.52898	-2.05457	-1.61163
H	+3.04487	-1.75472	-2.53651
C	+4.32918	-3.19760	-1.56723
H	+4.49102	-3.77407	-2.47401
C	+4.89354	-3.61641	-0.35933
H	+5.50278	-4.51576	-0.32329
C	+3.17671	+1.40479	-1.50369
C	-5.08711	+2.04546	-3.39947
H	-5.94888	+2.08015	-4.06063
C	+3.88858	-1.71544	+0.75901
H	+3.7255	-1.14184	+1.66473
C	+2.72436	+2.73197	-1.43870
H	+1.87349	+2.97552	-0.81171
C	+3.37205	+3.74156	-2.14999
H	+3.01184	+4.76367	-2.07677
C	-1.38091	+0.47658	+1.77396
C	+4.47466	+3.43630	-2.95364
H	+4.97847	+4.22077	-3.51227
C	+4.28342	+1.10670	-2.30902
H	+4.66130	+0.09205	-2.37114
C	-5.56560	+2.01446	+3.01391
H	-6.50331	+2.08274	+3.56013
C	-4.65617	+1.00535	+3.32891
H	-4.87518	+0.29477	+4.12208
C	+4.92514	+2.11638	-3.03161
H	+5.78390	+1.86875	-3.65064
C	-5.27862	+2.95792	+2.01010
H	-5.99106	+3.74973	+1.79403
C	-3.44634	+0.92450	+2.61675
C	-2.29597	+0.06463	+2.72510
C	-4.08139	+2.90117	+1.29139
H	-3.85097	+3.64738	+0.54216
C	-3.18867	+1.86852	+1.58134
C	+5.28323	+2.84437	+2.06226
H	+6.33514	+3.01254	+1.84612
C	-4.45763	+0.82948	-3.12867
H	-4.82184	-0.09054	-3.57782
C	-2.11443	-1.00809	+3.75971
H	-2.98045	-1.04825	+4.42937
H	-1.22758	-0.83031	+4.38048
H	-1.99842	-2.00228	+3.31078
C	-4.59284	+3.22189	-2.82779
H	-5.06988	+4.17456	-3.04224
C	+4.66286	-2.87664	+0.80452
H	+5.09203	-3.19867	+1.75000
C	-3.34683	+0.79136	-2.28343
H	-2.85163	-0.15507	-2.09793
C	-3.48791	+3.18105	-1.97732
H	-3.11741	+4.10688	-1.55020
C	-0.72708	+3.51659	-0.56982
C	+0.09499	+5.46773	+0.61797
H	+0.27172	+5.98732	+1.55636
C	+0.45115	+6.06636	-0.59315
H	+0.90802	+7.05230	-0.60196
C	+0.21788	+5.39034	-1.79603
H	+0.49404	+5.84901	-2.74171
C	-0.49858	+4.20284	+0.63060
H	-0.78387	+3.74715	+1.57225
C	-0.35663	+4.12007	-1.78494
H	-0.51072	+3.58806	-2.72113
C	+0.35369	+0.09210	-2.94918
O	-0.48391	+0.73508	-3.88555
O	+1.37463	-0.35724	-3.47238
H	-1.254	+1.05738	-3.39028

Ni-TIM_CO2H_q0_s_3H2O_MN15_6-311++Gdp_R

E = -5582.1713122
0,1
Ni -0.10037 +0.05134 +0.54502
P +1.87895 -1.09135 +0.83060
P -1.77093 -1.09255 -0.34456
P -0.04764 +2.26726 +0.59793
C +1.33397 -1.74281 -2.96841
N +2.09743 -1.59573 -0.82554
N -1.78937 -0.39016 -1.92650
N +1.04642 +2.56757 -0.72844
C +0.53120 +0.24273 -1.41796
C +1.23709 -1.03003 -1.79468
C +2.33132 -2.76521 -2.75196
C +2.87878 -3.75583 -3.58544
H +2.53713 -3.86128 -4.61183
C +3.86953 -4.59366 -3.08078
H +4.30141 -5.36161 -3.71739
C +4.32565 -4.45646 -1.75476
H +5.10156 -5.12008 -1.38246
C +3.80142 -3.48120 -0.90771
H +4.15436 -3.38175 +0.11107
C +2.80771 -2.64038 -1.41876
C +0.66062 -1.49777 -4.28809
H +0.86736 -2.32392 -4.97633
H +1.01759 -0.57689 -4.76718
H -0.42552 -1.41115 -4.19575
C +1.82368 -2.65063 +1.78612
C +1.38957 -3.84995 +1.20722
H +1.11517 -3.88312 +0.16038
C +1.30353 -5.01311 +1.97519
H +0.97240 -5.93596 +1.50794
C +1.62685 -4.98678 +3.33199
H +1.55779 -5.89314 +3.92758
C +2.03088 -3.78628 +3.92427
H +2.27330 -3.75300 +4.98309
C +2.12848 -2.62517 +3.15875
H +2.44529 -1.7007 +3.62946
C +3.51774 -0.36124 +1.26800
C +4.60693 -1.11604 +1.73634
H +4.49791 -2.17732 +1.93090
C +5.84107 -0.50782 +1.97089
H +6.67277 -1.10610 +2.33309
C +6.00834 +0.86072 +1.73990
H +6.97088 +1.33093 +1.92260
C +4.93188 +1.62124 +1.27952
H +5.04442 +2.68692 +1.10009
C +3.69718 +1.01340 +1.05042
H +2.87006 +1.61748 +0.70924
C -0.69836 +0.44566 -2.25820
C -1.01975 +1.19167 -3.36697
C -3.18783 +1.18781 -4.82443
H -2.85468 +1.93783 -5.53722
C -2.35474 +0.79751 -3.76399
C -4.44148 +0.59122 -4.95154
H -5.09606 +0.88348 -5.76859
C -0.19613 +2.16958 -4.14769
H -0.79638 +3.04799 -4.41698
H +0.15732 +1.72591 -5.08925
H +0.67894 +2.51301 -3.59620
C -4.05926 -0.8037 -2.98226
H -4.38514 -1.56795 -2.28458
C +0.63583 +3.10352 +2.08491
C -2.80718 -0.19787 -2.85961
C -1.73992 -2.89529 -0.64678
C -1.71155 -3.77485 +0.44659
H -1.69079 -3.40120 +1.46913
C -1.67705 -5.15394 +0.22927
H -1.66017 -5.82626 +1.08242
C -1.62965 -5.66501 -1.06945
H -1.58722 -6.73851 -1.23296
C -3.44867 -0.70887 +0.29615
C +1.72120 +4.27481 +4.39784
H +2.14312 +4.72948 +5.29009
C -1.68964 -3.41060 -1.95221
H -1.69645 -2.74146 -2.80472
C -3.95379 +0.58449 +0.06904
H -3.40506 +1.28183 -0.55490
C -5.15694 +0.98174 +0.65358
H -5.53662 +1.98067 +0.46381
C +1.45720 +1.42231 -1.45574
C -5.85487 +0.10552 +1.48786
H -6.7841 +0.42175 +1.95473
C -4.16018 -1.58667 +1.12625
H -3.78956 -2.58600 +1.32274
C +4.10075 +5.19489 -1.72569
H +4.92577 +5.83957 -2.01740
C +4.06305 +3.87692 -2.17565
H +4.84703 +3.49267 -2.82276
C -5.35343 -1.17677 +1.72329
H -5.87442 -1.84345 +2.40211
C +3.07511 +5.70930 -0.91014
H +3.11563 +6.7454 -0.58499
C +2.99149 +3.05464 -1.79021
C +2.64577 +1.68284 -2.09547
C +1.99622 +4.91551 -0.5194
H +1.19746 +5.32506 +0.08505
C +1.97658 +3.58776 -0.95084
C -4.87127 -0.39364 -4.04090
H -5.85101 -0.84733 -4.16441
C +1.97194 +2.93160 +4.10984
H +2.58535 +2.33216 +4.77690
C +3.45815 +0.78452 -2.98315
H +4.36199 +1.30207 -3.32011
H +2.90471 +0.47281 -3.87538
H +3.77630 -0.12411 -2.46062
C +0.91442 +5.02971 +3.54184

H +0.70424 +6.07189 +3.76586
C -1.62542 -4.78923 -2.15903
H -1.57759 -5.17708 -3.17299
C +1.42589 +2.34767 +2.96635
H +1.60491 +1.29942 +2.76730
C +0.37204 +4.44877 +2.39556
H -0.26511 +5.04458 +1.75086
C -1.52695 +3.26038 +0.18404
C -2.95620 +4.31532 -1.46866
H -3.12724 +4.62743 -2.49542
C -3.92404 +4.55392 -0.48974
H -4.85399 +5.05136 -0.75103
C -3.68922 +4.15593 +0.82978
H -4.43445 +4.34286 +1.59777
C -1.76012 +3.67740 -1.13407
H -1.00947 +3.50446 -1.89521
C -2.50161 +3.50681 +1.16624
H -2.33607 +3.19750 +2.19444
C -0.70334 -0.06963 +2.35402
O -1.93728 +0.51694 +2.69353
O -0.12137 -0.55092 +3.31267
H -2.40438 +0.79528 +1.88317
O -3.23756 -1.12173 +4.50566
H -2.88625 -0.46732 +3.85733
H -0.66743 -2.58790 +3.52142
H -5.09018 -1.04418 +4.70737
O -6.06058 -0.96193 +4.82537
H -6.26918 -0.16239 +4.31636
H -2.80358 -0.87626 +5.34242
O -1.44684 -3.15833 +3.63102
H -2.13461 -2.52438 +3.92304

Ni-TIM_CO2H_q0_s_3H2O_MN15_6-311++Gdp_TS
E = -5582.1346081
0,1
Ni -0.03610 -0.00730 +0.56212
P +2.15382 -0.62573 +0.82883
P -1.46896 -1.52297 -0.27450
P -0.62239 +2.18451 +0.50809
C +1.69711 -1.54348 -2.91011
N +2.44294 -1.15000 -0.79431
N -1.62146 -0.89548 -1.87594
N +0.45677 +2.66364 -0.77719
C +0.49204 +0.26319 -1.39696
C +1.46133 -0.83042 -1.75723
C +2.90115 -2.31159 -2.68081
C +3.64070 -3.19255 -3.48882
H +3.32161 -3.40906 -4.50478
C +4.79170 -3.77915 -2.97085
H +5.37216 -4.46050 -3.58725
C +5.21923 -3.50044 -1.65736
H +6.12151 -3.97045 -1.27558
C +4.50680 -2.62949 -0.83530
H +4.83714 -2.41888 +0.17427
C +3.35474 -2.03966 -1.36293
C +0.96509 -1.50650 -4.21985
H +1.36273 -2.26831 -4.89763
H +1.07027 -0.53580 -4.71905
H -0.10554 -1.69500 -4.10653
C +2.39222 -2.10009 +1.87917
C +2.1814 -3.39214 +1.37889
H +1.95289 -3.54597 +0.33121
C +2.25855 -4.49478 +2.23162
H +2.10007 -5.49048 +1.829
C +2.51768 -4.31839 +3.59173
H +2.56894 -5.17862 +4.25342
C +2.70657 -3.02986 +4.10093
H +2.90421 -2.88252 +5.15902
C +2.64691 -1.92564 +3.25111
H +2.80082 -0.93142 +3.65891
C +3.56674 +0.47654 +1.23662
C +4.79119 -0.01285 +1.72324
H +4.91590 -1.06955 +1.93246
C +5.85602 +0.85812 +1.95509
H +6.79692 +0.46626 +2.33138
C +5.71372 +2.22621 +1.70519
H +6.54487 +2.90185 +1.88780
C +4.49946 +2.72219 +1.22665
H +4.37500 +3.78399 +1.03376
C +3.43266 +1.85302 +0.99672
H +2.49571 +2.25248 +0.63766
C -0.75630 +0.16317 -2.22761
C -1.22392 +0.78089 -3.36258
C -3.30743 +0.22178 -4.83559
H -3.14935 +1.00772 -5.56925
C -2.42115 +0.06860 -3.75736
C -4.38492 -0.65411 -4.94896
H -5.07951 -0.54838 -5.77805
C -0.64119 +1.89366 -4.17906
H -0.15115 +1.50346 -5.08198
H +0.09596 +2.48085 -3.63170
H -1.43448 +2.57197 -4.51590
C -3.71770 -1.85887 -2.93018
H -3.86771 -2.65264 -2.20614
C -0.19987 +3.22848 +1.95889
C -2.64162 -0.97552 -2.82405
C -1.05603 -3.28768 -0.51215
C -0.86024 -4.10748 +0.61016
H -0.94226 -3.70489 +1.61947
C -0.53139 -5.45357 +0.43606
H -0.38751 -6.0824 +1.31026
C -0.35822 -5.98154 -0.84518
H -0.08784 -7.02624 -0.97369

C -3.17890 -1.45160 +0.38011
C +0.51197 +4.75391 +4.20887
H +0.78658 +5.34444 +5.07869
C -0.87956 -3.81824 -1.80056
H -1.01718 -3.19369 -2.67487
C -3.99993 -0.37726 -0.00744
H -3.64868 +0.34748 -0.73250
C -5.26596 -0.22324 +0.55796
H -5.88951 +0.60910 +0.24676
C +1.12200 +1.62315 -1.46633
C -5.71209 -1.11656 +1.53485
H -6.68998 -0.98271 +1.99004
C -3.63056 -2.34195 +1.36506
H -2.99784 -3.14532 +1.72018
C +2.82303 +5.88725 -1.88084
H +3.47787 +6.69490 -2.19704
C +3.08630 +4.58424 -2.29436
H +3.93771 +4.36936 -2.93434
C -4.88941 -2.16935 +1.94109
H -5.20408 -2.82002 +2.74923
C +1.70929 +6.17698 -1.06844
H +1.51477 +7.20388 -0.77120
C +2.23073 +3.55073 -1.87713
C +2.21253 +2.12719 -2.13409
C +0.84234 +5.17127 -0.64345
H -0.02325 +5.40388 -0.03635
C +1.12334 +3.86218 -1.04309
C -4.58687 -1.68232 -4.00662
H -5.43323 -2.35410 -4.12038
C +1.17258 +3.55309 +3.93938
H +1.96375 +3.20168 +4.59581
C +3.19762 +1.41107 -3.01148
H +3.93942 +2.11443 -3.40246
H +2.71216 +0.93444 -3.86950
H +3.73994 +0.63030 -2.46710
C -0.50848 +5.18959 +3.36006
H -1.03020 +6.12049 +3.56437
C -0.52447 -5.15804 -1.96221
H -0.38345 -5.55707 -2.96304
C +0.81266 +2.79177 +2.82768
H +1.32704 +1.85872 +2.63847
C -0.86358 +4.43338 +2.24243
H -1.65685 +4.78749 +1.59332
C -2.25164 +2.80564 -0.04570
C -3.70281 +3.63621 -1.80443
H -3.83574 +3.95830 -2.83372
C -4.78481 +3.63312 -0.92134
H -5.76720 +3.95028 -1.26049
C -4.59791 +3.22576 +0.40293
H -5.43416 +3.22401 +1.09650
C -2.43926 +3.23231 -1.36866
H -1.60172 +3.25771 -2.05418
C -3.34224 +2.80525 +0.84070
H -3.21223 +2.46396 +1.86292
C -0.33845 -0.22092 +2.36360
O -2.36264 +0.50821 +2.80475
O +0.01304 -0.41993 +3.44476
H -2.91111 +0.25339 +2.04682
O -2.74234 -1.28646 +4.44869
H -2.57435 -0.27535 +3.56649
H -0.23402 -2.82087 +3.75635
H -4.33224 -1.62079 +4.84723
O -5.30458 -1.77195 +5.05339
H -5.73621 -1.13001 +4.46859
H -2.31462 -0.96345 +5.25883
O -1.11253 -3.21537 +3.64705
H -1.74683 -2.48561 +3.9466

Ni-TIM_CO2H_q0_s_MN15_6-311++gdp
E = -5352.9764065
0,1
Ni -0.04564 +0.01010 -0.80985
P +2.22047 -0.09936 -0.62214
P -1.05534 +1.93275 -0.60737
P -1.16424 -1.91588 -0.63684
C +1.38779 +1.70183 +2.72205
N +2.35966 +0.64332 +0.95297
N -1.70730 +1.78111 +0.99097
N -0.65204 -2.38102 +0.97343
C -0.02235 +0.00899 +1.25671
C +1.16128 +0.83314 +1.67859
C +2.79313 +2.03605 +2.68701
C +3.60571 +2.83676 +3.50809
H +3.17977 +3.36177 +4.35921
C +4.96427 +2.94281 +3.22144
H +5.60122 +3.55797 +3.85190
C +5.52673 +2.25932 +2.12579
H +6.58957 +2.35547 +1.92022
C +4.74414 +1.45654 +1.29670
H +5.18280 +0.93728 +0.45422
C +3.38121 +1.34933 +1.5895
C +0.43908 +2.17298 +3.78662
H +0.91437 +2.94481 +4.40092
H +0.13527 +1.36101 +4.45973
H -0.47569 +2.60337 +3.36938
C +3.20326 +0.96727 -1.73504
C +3.36339 +2.33715 -1.49151
H +2.95292 +2.78434 -0.59427
C +4.04798 +3.13919 -2.40725
H +4.16997 +4.19848 -2.20043
C +4.56094 +2.58725 -3.58238
H +5.09349 +3.21383 -4.29290
C +4.37972 +1.22563 -3.84463
H +4.76661 +0.78904 -4.76155

C +3.70274 +0.42102 -2.92919
H +3.56964 -0.63556 -3.14328
C +3.25903 -1.61962 -0.45965
C +4.65472 -1.62251 -0.62749
H +5.17278 -0.72039 -0.93276
C +5.39219 -2.78883 -0.41941
H +6.47044 -2.77216 -0.55375
C +4.74919 -3.97124 -0.04138
H +5.32582 -4.87822 +0.11959
C +3.36292 -3.98226 +0.12183
H +2.84798 -4.89541 +0.40758
C +2.62642 -2.81581 -0.08902
H +1.55397 -2.84533 +0.02653
C -1.30765 +0.63541 +1.71899
C -2.18597 +0.42818 +2.75560
C -4.27703 +1.81164 +3.48995
H -4.53431 +1.19363 +4.34639
C -3.16829 +1.48988 +2.69149
C -5.03496 +2.93713 +3.16905
H -5.89670 +3.19725 +3.77840
C -2.16787 -0.59449 +3.85037
H -3.17616 -0.99390 +4.01867
H -1.84034 -0.14973 +4.80095
H -1.50436 -1.43144 +3.63188
C -3.59441 +3.45449 +1.26435
H -3.33086 +4.08111 +0.41887
C -0.71690 -3.29122 -1.77573
C -2.84324 +2.32359 +1.59016
C -0.12050 +3.50112 -0.59564
C +0.54393 +3.89467 -1.76869
H +0.50055 +3.26428 -2.65274
C +1.28075 +5.07954 -1.79020
H +1.78697 +5.37979 -2.70292
C +1.39298 +5.85778 -0.63507
H +1.97929 +6.77255 -0.64986
C -2.55127 +2.26777 -1.61783
C +0.07004 -5.30504 -3.57324
H +0.37591 -6.08640 -4.26382
C -0.00571 +4.28372 +0.56286
H -0.50745 +3.98149 +1.47566
C -3.63726 +1.39328 -1.45229
H -3.57357 +0.58320 -0.73424
C -4.80200 +1.56712 -2.19693
H -5.63542 +0.88598 -2.05259
C +0.11594 -1.42329 +1.68219
C -4.89181 +2.60803 -3.12638
H -5.79923 +2.74198 -3.70912
C -2.64683 +3.31242 -2.54644
H -1.82695 +4.00937 -2.68149
C +0.29892 -5.80403 +3.12189
H +0.58543 -6.66832 +3.71553
C +0.84838 -4.55674 +3.40913
H +1.55514 -4.44125 +4.22671
C -3.81282 +3.47686 -3.29959
H -3.87640 +4.29121 -4.01644
C -0.63704 -5.95800 +2.08132
H -1.06682 -6.93667 +1.88506
C +0.46936 -3.44788 +2.63401
C +0.82141 -2.04683 +2.68501
C -1.03358 -4.87219 +1.30056
H -1.77109 -4.99269 +0.51845
C -0.46124 -3.62784 +1.57439
C -4.69709 +3.74829 +2.06837
H -5.30204 +4.62187 +1.84039
C +0.87617 -4.1794 -3.38583
H +1.80937 -4.07511 -3.93263
C +1.74240 -1.44056 +3.70470
H +2.12397 -2.21362 +4.37954
H +1.23737 -0.68720 +4.31892
H +2.60768 -0.95509 +3.23951
C -1.13900 -5.41509 -2.88176
H -1.77952 -6.27941 -3.03463
C +0.75763 +5.45239 +0.54231
H +0.85020 +6.04865 +1.44609
C +0.47852 -3.17585 -2.50305
H +1.09643 -2.29376 -2.38342
C -1.53296 -4.41332 -1.99291
H -2.48462 -4.50536 -1.48013
C -2.98839 -2.01732 -0.53001
C -5.03014 -1.92479 +0.77999
H -5.51892 -1.88883 +1.75001
C -5.79059 -1.92252 -0.39171
H -6.87523 -1.88259 -0.33876
C -5.15040 -1.97464 -1.63414
H -5.73545 -1.97557 -2.54980
C -3.63613 -1.97823 +0.71345
H -3.05369 -1.98705 +1.62710
C -3.75822 -2.01418 -1.70581
H -3.26827 -2.03017 -2.67373
C -0.17846 +0.15814 -2.70760
O -1.24940 -0.51594 -3.26814
O +0.52433 +0.81517 -3.46329
H -1.23186 -0.31977 -4.23107

Ni-TIM_CO2H_q1_d_MN15_6-311++gdp
E = -5352.7962103
1,2
Ni +0.07979 -0.01909 -0.80941
P -2.10691 +0.80564 -0.59273
P +0.40271 -2.19992 -0.60644
P +1.72966 +1.49664 -0.62265
C -1.81791 -1.32607 +2.68154
N -2.45449 +0.01575 +0.93780
N +1.00742 -2.23167 +1.02583

N	+1.35030	+2.05013	+1.00422
C	-0.00157	-0.01382	+1.37228
C	-1.36198	-0.49027	+1.66525
C	-3.24905	-1.30918	+2.60850
C	-4.24217	-1.93791	+3.38400
H	-3.96333	-2.57934	+4.21506
C	-5.57619	-1.723	+3.06594
H	-6.35641	-2.19976	+3.65249
C	-5.93311	-0.88893	+1.98559
H	-6.98349	-0.73609	+1.75384
C	-4.97096	-0.25173	+1.20453
H	-5.26301	+0.37531	+0.37307
C	-3.62777	-0.46401	+1.52705
C	-1.01148	-2.01740	+3.73523
H	-1.66315	-2.41066	+4.52077
H	-0.29539	-1.33143	+4.20056
H	-0.43305	-2.85650	+3.33072
C	-3.35340	+0.14735	-1.74932
C	-3.79909	-1.17715	-1.64851
H	-3.45954	-1.80728	-0.83515
C	-4.68245	-1.69323	-2.59694
H	-5.02784	-2.71826	-2.50124
C	-5.10974	-0.90168	-3.66566
H	-5.79656	-1.30691	-4.40379
C	-4.64605	+0.41125	-3.78579
H	-4.96742	+1.03095	-4.61851
C	-3.77241	+0.93620	-2.83308
H	-3.42525	+1.96023	-2.93353
C	-2.63121	+2.54810	-0.31650
C	-3.98273	+2.93703	-0.28525
H	-4.76623	+2.21786	-0.49360
C	-4.33129	+4.25803	-0.00613
H	-5.37965	+4.54269	+0.01544
C	-3.33841	+5.21160	+0.24130
H	-3.61350	+6.24023	+0.45813
C	-1.99445	+4.83846	+0.20297
H	-1.21271	+5.56935	+0.38882
C	-1.64627	+3.51586	-0.07529
H	-0.60219	+3.24497	-0.10921
C	+1.05433	-0.98467	+1.70045
C	+2.02572	-1.02763	+2.68708
C	+3.58969	-2.97526	+3.41748
H	+4.08093	-2.43547	+4.22216
C	+2.59893	-2.34626	+2.64190
C	+3.92364	-4.29311	+3.12934
H	+4.68975	-4.79586	+3.71296
C	+2.36034	-0.01210	+3.73173
H	+3.43446	+0.20989	+3.72691
H	+2.12124	-0.40022	+4.73049
H	+1.81817	+0.92373	+3.59444
C	+2.28395	-4.39937	+1.31437
H	+1.78375	-4.94247	+0.52023
C	+1.68554	+2.93434	-1.76123
C	+1.95380	-3.07443	+1.60745
C	-0.97229	-3.38887	-0.64954
C	-1.64020	-3.59856	-1.86782
H	-1.33055	-3.06144	-2.75971
C	-2.72201	-4.47724	-1.92572
H	-3.23137	-4.63926	-2.87103
C	-3.16651	-5.12165	-0.76783
H	-4.01933	-5.79332	-0.81304
C	+1.77102	-2.95407	-1.56154
C	+1.52932	+5.06164	-3.58853
H	+1.46856	+5.88702	-4.29267
C	-1.41533	-4.04147	+0.50950
H	-0.90480	-3.88058	+1.45178
C	+3.04357	-2.38702	-1.38606
H	+3.17705	-1.55905	-0.69839
C	+4.13971	-2.89300	-2.08062
H	+5.11906	-2.44844	-1.93121
C	+0.26850	+1.40194	+1.65397
C	+3.97473	-3.96179	-2.96731
H	+4.82910	-4.35394	-3.51223
C	+1.60979	-4.03011	-2.44383
H	+0.63997	-4.49502	-2.58132
C	+1.30955	+5.67233	+3.03987
H	+1.25704	+6.60504	+3.59427
C	+0.38272	+4.66770	+3.28805
H	-0.39615	+4.79874	+4.03370
C	+2.71068	-4.52643	-3.14722
H	+2.5769	-5.36129	-3.82955
C	+2.33136	+5.4921	+2.08388
H	+3.05742	+6.28416	+1.92343
C	+0.46999	+3.46944	+2.55554
C	-0.30377	+2.25977	+2.58416
C	+2.44226	+4.31346	+1.34814
H	+3.24797	+4.17514	+0.63957
C	+1.49444	+3.31538	+1.58134
C	+3.27761	-4.99514	+2.08833
H	+3.55662	-6.02589	+1.88838
C	+0.43627	+4.20943	-3.41226
H	-0.47738	+4.36349	-3.97949
C	-1.46537	+1.99564	+3.48724
H	-1.52915	+2.75806	+4.26892
H	-1.38641	+1.01743	+3.97156
H	-2.41070	+2.00516	+2.92909
C	+2.70692	+4.83968	-2.87007
H	+3.56641	+5.48823	-3.01471
C	-2.51676	-4.89686	+0.44890
H	-2.86226	-5.39071	+1.35292
C	+0.51785	+3.14829	-2.51189
H	-0.32963	+2.48257	-2.39380
C	+2.78842	+3.77990	-1.96617
H	+3.71869	+3.60604	-1.43634
C	+3.50274	+1.08000	-0.50666
C	+5.46282	+0.53628	+0.81496

H +5.93814 +0.43565 +1.78692
C +6.18073 +0.26400 -0.35150
H +7.21753 -0.05545 -0.29227
C +5.56254 +0.40922 -1.59777
H +6.11689 +0.20351 -2.50922
C +4.13022 +0.94652 +0.73945
H +3.58398 +1.17290 +1.64661
C +4.22955 +0.80993 -1.67856
H +3.75187 +0.90208 -2.64779
C +0.21497 -0.21820 -2.66849
O +1.44225 +0.03786 -3.21131
O -0.70149 -0.57661 -3.38571
H +1.37599 -0.12558 -4.17869

Ni-TIM_CO2_q-1_d_3H2O_MN15_6-311++Gdp_R
E = -5582.2675531
-1,2
Ni -0.07220 -0.01257 +0.68817
P +1.84795 +1.09181 +0.90360
P +0.10352 -2.20283 +0.09958
P -1.84697 +1.17114 -0.00636
C +2.87239 -0.52902 -2.49497
N +2.69751 +0.69454 -0.57604
N -0.22484 -2.17877 -1.61268
N -1.25078 +1.95911 -1.45687
C +0.49864 +0.19309 -1.64353
C +1.97091 +0.05127 -1.62315
C +4.19552 -0.20386 -2.02843
C +5.47927 -0.47515 -2.53232
H +5.59864 -1.06297 -3.43927
C +6.59267 +0.02870 -1.86139
H +7.59010 -0.17385 -2.24398
C +6.44214 +0.80269 -0.69522
H +7.32258 +1.18854 -0.18788
C +5.17696 +1.08912 -0.17705
H +5.06945 +1.68465 +0.72091
C +4.06272 +0.58107 -0.84901
C +2.59961 -1.23937 -3.78713
H +3.36897 -1.99711 -3.98064
H +2.61149 -0.54777 -4.64364
H +1.62936 -1.73942 -3.78886
C +2.87093 +0.425 +2.26957
C +3.72328 -0.67705 +2.12451
H +3.89173 -1.11182 +1.14682
C +4.35452 -1.23293 +3.24024
H +5.01648 -2.08395 +3.10771
C +4.12760 -0.71115 +4.51511
H +4.61896 -1.14782 +5.38049
C +3.25378 +0.36971 +4.67262
H +3.05905 +0.77607 +5.66179
C +2.62695 +0.92998 +3.56026
H +1.94695 +1.76737 +3.69521
C +2.15341 +2.91640 +1.01587
C +3.24038 +3.50214 +1.68609
H +3.94606 +2.88428 +2.23115
C +3.42471 +4.88625 +1.67120
H +4.27203 +5.31997 +2.19606
C +2.52972 +5.71000 +0.98243
H +2.67711 +6.78671 +0.97049
C +1.44092 +5.14092 +0.31820
H +0.73057 +5.76763 -0.21454
C +1.25358 +3.75841 +0.34333
H +0.38965 +3.33293 -0.14598
C -0.23669 -0.91759 -2.27429
C -0.92642 -1.05076 -3.46614
C -2.02865 -3.14267 -4.56801
H -2.38626 -2.63498 -5.46065
C -1.34137 -2.42609 -3.57340
C -2.23643 -4.51112 -4.39636
H -2.76836 -5.07482 -5.15903
C -1.11455 -0.04407 -4.56141
H -2.12316 -0.11664 -4.98893
H -0.40856 -0.21048 -5.389
H -0.97096 +0.97936 -4.21098
C -1.06275 -4.48785 -2.25381
H -0.68566 -5.00533 -1.37878
C -2.41286 +2.53397 +1.11880
C -0.87083 -3.11497 -2.42128
C +1.64598 -3.19097 +0.20786
C +2.18961 -3.45660 +1.47639
H +1.67671 -3.09102 +2.36103
C +3.38904 -4.16072 +1.59593
H +3.79475 -4.36615 +2.58280
C +4.07881 -4.57597 +0.45355
H +5.02010 -5.11089 +0.54856
C -1.20631 -3.32892 +0.73716
C -3.12934 +4.50859 +3.00224
H -3.40869 +5.26851 +3.72684
C +2.34098 -3.61593 -0.93555
H +1.93670 -3.41228 -1.92108
C -2.53153 -2.91895 +0.52178
H -2.72644 -2.02294 -0.05626
C -3.59849 -3.65740 +1.03087
H -4.61621 -3.32341 +0.84817
C +0.01490 +1.54382 -1.96701
C -3.35552 -4.81587 +1.77581
H -4.18467 -5.38977 +2.18121
C -0.97039 -4.49443 +1.47696
H +0.04285 -4.83919 +1.65307
C -1.57003 +5.70286 -3.23516
H -1.59893 +6.67570 -3.71986
C -0.44438 +4.89318 -3.38090
H +0.39825 +5.22406 -3.98301

C -2.04004 -5.22995 +1.99568
H -1.84103 -6.13037 +2.57130
C -2.67755 +5.27116 -2.48165
H -3.55284 +5.90960 -2.39499
C -0.41556 +3.63717 -2.74946
C +0.54245 +2.56145 -2.74022
C -2.67683 +4.02451 -1.85078
H -3.54345 +3.68569 -1.29763
C -1.53574 +3.22927 -1.96950
C -1.75724 -5.17671 -3.25212
H -1.92354 -6.24524 -3.14238
C -1.81840 +4.03319 +2.95110
H -1.06678 +4.41686 +3.63554
C +1.81936 +2.53662 -3.52952
H +1.91292 +3.44242 -4.13842
H +1.86103 +1.67646 -4.2092
H +2.70642 +2.48049 -2.88666
C -4.08578 +3.99386 +2.12083
H -5.11093 +4.35237 +2.15749
C +3.55466 -4.29489 -0.81180
H +4.08726 -4.60926 -1.7058
C -1.46807 +3.04804 +2.02520
H -0.45216 +2.66970 +2.01055
C -3.73217 +3.01588 +1.18982
H -4.49257 +2.62731 +0.52125
C -3.43197 +0.45112 -0.58345
C -4.85104 -0.41623 -2.35459
H -5.04157 -0.55825 -3.41541
C -5.75273 -0.90685 -1.40702
H -6.64757 -1.43498 -1.72542
C -5.49997 -0.70788 -0.04576
H -6.20111 -1.07192 +0.70073
C -3.70189 +0.26562 -1.94698
H -3.01345 +0.65118 -2.68934
C -4.34385 -0.04451 +0.36532
H -4.17273 +0.10318 +1.42741
C -0.47198 -0.54424 +2.57562
O -1.39023 +0.16089 +3.41617
O -0.03857 -1.55105 +3.13853
H -1.71794 +0.92630 +2.91242
O -3.52874 -1.08265 +4.56671
H -2.70422 -0.67277 +4.19928
H -0.90010 -2.54452 +4.47063
H -4.68148 +0.19084 +3.86631
O -5.19444 +0.92584 +3.46510
H -4.52259 +1.60263 +3.28292
H -3.49027 -0.86808 +5.51629
O -1.47091 -3.07250 +5.06866
H -2.35214 -2.71726 +4.85788

Ni-TIM_CO2_q-1_d_4H2O_R_MN15

E = -5658.1039833
-1,1
Ni -0.50036 +1.78684 +9.15882
P +1.3076 +0.71594 +9.97414
P -1.05290 +1.56691 +7.08528
P -1.35517 +3.41180 +10.3767
C +3.02144 +2.61182 +7.03848
N +2.51291 +1.37912 +8.88656
N -0.40875 +3.03145 +6.39612
N +0.03358 +4.49347 +10.4385
C +0.88779 +3.21592 +8.47725
C +2.10853 +2.45343 +8.05966
C +4.06476 +1.63797 +7.25365
C +5.26072 +1.34989 +6.57326
H +5.53512 +1.91051 +5.68305
C +6.09264 +0.34304 +7.05727
H +7.02022 +0.11508 +6.53798
C +5.74975 -0.38221 +8.21474
H +6.41329 -1.16339 +8.57645
C +4.57045 -0.11487 +8.91068
H +4.31284 -0.67826 +9.79837
C +3.73754 +0.89702 +8.42394
C +3.03786 +3.63632 +5.94019
H +3.90343 +3.47888 +5.28806
H +3.10253 +4.66065 +6.32710
H +2.14186 +3.58813 +5.31197
C +1.41033 -1.09246 +9.70750
C +1.73883 -1.62723 +8.45532
H +2.00978 -0.97181 +7.63530
C +1.70617 -3.00788 +8.24739
H +1.96953 -3.40645 +7.27187
C +1.32449 -3.86767 +9.27902
H +1.29882 -4.94155 +9.11378
C +0.96725 -3.33850 +10.5232
H +0.65613 -3.99815 +11.3291
C +1.00610 -1.96090 +10.7344
H +0.71867 -1.56248 +11.7032
C +2.11681 +0.95804 +11.6238
C +3.03901 +0.05798 +12.1849
H +3.26310 -0.87879 +11.6870
C +3.66837 +0.34648 +13.3969
H +4.37813 -0.36283 +13.8144
C +3.39014 +1.54020 +14.0690
H +3.88308 +1.76327 +15.0116
C +2.47185 +2.44032 +13.5251
H +2.24020 +3.36987 +14.0381
C +1.83941 +2.14679 +12.3162
H +1.11962 +2.84556 +11.9174
C +0.25715 +3.89619 +7.30148
C +0.25148 +5.17463 +6.79286
C -0.65622 +6.07529 +4.51303
H -0.35168 +7.10857 +4.66022
C -0.40281 +5.11325 +5.50365

C -1.29902 +5.68235 +3.33887
H -1.50087 +6.41781 +2.56417
C +0.88293 +6.42234 +7.33105
H +0.19715 +7.27430 +7.23612
H +1.79187 +6.68470 +6.76990
H +1.16065 +6.32651 +8.38128
C -1.43516 +3.3663 +4.10326
H -1.72607 +2.33200 +3.95017
C -1.83708 +3.05468 +12.1210
C -0.79064 +3.76687 +5.27441
C -0.36204 +0.24014 +6.02904
C -0.73033 -1.09378 +6.27131
H -1.41643 -1.33834 +7.07355
C -0.18797 -2.12151 +5.49920
H -0.49692 -3.14593 +5.68765
C +0.76046 -1.83951 +4.51294
H +1.19225 -2.64394 +3.92329
C -2.84042 +1.68088 +6.65189
C -2.5263 +2.33875 +14.7529
H -2.78965 +2.06501 +15.7712
C +0.59599 +0.51994 +5.04061
H +0.90333 +1.54360 +4.85494
C -3.45422 +2.94310 +6.71039
H -2.86990 +3.82301 +6.95593
C -4.82057 +3.07779 +6.46565
H -5.27407 +4.06379 +6.50364
C +1.1465 +4.11965 +9.63992
C -5.60115 +1.95014 +6.19925
H -6.66916 +2.05437 +6.02506
C -3.63093 +0.55238 +6.39166
H -3.20440 -0.44374 +6.37226
C +1.8867 +7.08328 +13.1046
H +2.42267 +7.74682 +13.7786
C +2.58485 +6.39292 +12.1158
H +3.65923 +6.51990 +12.0094
C -5.00492 +0.68768 +6.17592
H -5.60759 -0.20479 +6.03685
C +0.49133 +6.94558 +13.2321
H -0.03686 +7.50714 +13.9982
C +1.88092 +5.53815 +11.2505
C +2.28365 +4.72824 +10.1250
C -0.23142 +6.10652 +12.3828
H -1.30679 +6.02676 +12.4696
C +0.47441 +5.39316 +11.4110
C -1.68525 +4.34318 +3.13704
H -2.18398 +4.06214 +2.21322
C -1.70882 +1.50093 +13.9896
H -1.33704 +0.56857 +14.4063
C +3.68331 +4.65994 +9.58381
H +4.35263 +5.29088 +10.1781
H +3.74075 +5.00742 +8.54613
H +4.08666 +3.64111 +9.60590
C -3.01829 +3.52164 +14.1953
H -3.66874 +4.17056 +14.7758
C +1.15962 -0.51733 +4.29482
H +1.90457 -0.28942 +3.53679
C -1.37871 +1.85241 +12.6811
H -0.77029 +1.18618 +12.0807
C -2.68076 +3.87441 +12.8869
H -3.09198 +4.78407 +12.4621
C -2.68994 +4.52564 +9.80020
C -3.42129 +6.54855 +8.67370
H -3.1789 +7.49391 +8.19505
C -4.7526 +6.14009 +8.78888
H -5.55135 +6.76565 +8.39928
C -5.05258 +4.92114 +9.40548
H -6.08531 +4.59334 +9.49002
C -2.39424 +5.74919 +9.18111
H -1.36431 +6.07812 +9.09841
C -4.02943 +4.11255 +9.89975
H -4.26118 +3.15193 +10.3480
C -1.88880 +0.51117 +9.60058
O -3.02104 +0.96827 +9.95835
O -1.60861 -0.71164 +9.47160
O -6.18609 -2.09260 +7.41895
H -5.94334 -1.46246 +8.12618
H -5.30619 -2.40386 +7.11379
H -3.40020 -3.77251 +6.65782
O -3.50057 -2.83932 +6.9095
H -3.39937 -2.83691 +7.90689
H -5.66221 -0.43933 +10.4877
O -5.17439 -0.57851 +9.65908
H -4.35217 +0.00083 +9.74161
H -2.69166 -1.99257 +9.63807
O -3.39714 -2.69740 +9.58444
H -4.20423 -2.14424 +9.69060

Ni-TIM_CO2_q-1_s_3H2O_CO2_P_MN15
E = -5770.1948949
-1,1
Ni -0.27007 -0.18335 +0.59638
P +1.78699 -0.66998 +1.46275
P -1.47858 -1.76686 -0.24871
P -0.66751 +1.97947 +0.18933
C +2.20928 -2.19520 -2.09275
N +2.46588 -1.42331 +0.03715
N -1.25710 -1.47387 -1.94543
N +0.59596 +2.28214 -0.99096
C +0.71675 -0.19182 -1.22816
C +1.73307 -1.29539 -1.16576
C +3.31887 -2.88245 -1.47407
C +4.21262 -3.86408 -1.93593
H +4.12057 -4.26117 -2.94360
C +5.22288 -4.31428 -1.08973

H +5.92045 -5.07136 -1.43896
C +5.35716 -3.79694 +0.21334
H +6.15383 -4.16087 +0.85684
C +4.48631 -2.82038 +0.69560
H +4.59453 -2.42911 +1.69893
C +3.47390 -2.36890 -0.15647
C +1.78027 -2.39646 -3.51775
H +2.32513 -3.23501 -3.96383
H +1.97584 -1.51195 -4.13656
H +0.71162 -2.61627 -3.60442
C +1.89560 -1.95197 +2.76222
C +1.83124 -3.31796 +2.45983
H +1.7842 -3.64909 +1.42932
C +1.82255 -4.26619 +3.48540
H +1.78064 -5.32207 +3.23388
C +1.85532 -3.86119 +4.82097
H +1.84547 -4.60168 +5.61645
C +1.89137 -2.49772 +5.12938
H +1.90067 -2.16990 +6.16545
C +1.91334 -1.54892 +4.10809
H +1.92927 -0.49449 +4.36421
C +3.09736 +0.53974 +1.95251
C +4.23075 +0.19005 +2.70718
H +4.33855 -0.81372 +3.10217
C +5.22450 +1.13248 +2.97454
H +6.09381 +0.84379 +3.55935
C +5.10396 +2.43981 +2.49409
H +5.88021 +3.17117 +2.70269
C +3.97839 +2.80185 +1.75230
H +3.86600 +3.81597 +1.37868
C +2.98355 +1.85896 +1.48984
H +2.11174 +2.15970 +0.93005
C -0.31819 -0.46853 -2.281
C -0.55312 -0.04073 -3.56620
C -2.32449 -0.84595 -5.31004
H -2.01130 -0.18474 -6.11409
C -1.66616 -0.81706 -4.07059
C -3.37832 -1.74066 -5.49332
H -3.89682 -1.77316 -6.44815
C +0.19674 +0.94875 -4.40567
H -0.50023 +1.54563 -5.00713
H +0.87307 +0.44351 -5.11037
H +0.79766 +1.63372 -3.80674
C -3.13431 -2.60665 -3.22164
H -3.43793 -3.28031 -2.42739
C -0.46814 +3.18327 +1.56630
C -2.08213 -1.70598 -3.04487
C -1.03262 -3.52058 -0.00617
C -1.10409 -4.05484 +1.29084
H -1.41338 -3.42471 +2.12041
C -0.75173 -5.38549 +1.51909
H -0.81471 -5.79209 +2.52417
C -0.28817 -6.17982 +0.46693
H +0.00056 -7.21127 +0.65006
C -3.30345 -1.70494 -0.06553
C -0.23069 +4.87563 +3.79863
H -0.14764 +5.52779 +4.66396
C -0.56709 -4.32008 -1.06057
H -0.49840 -3.91196 -2.06305
C -3.95045 -0.56548 -0.57115
H -3.37199 +0.21260 -1.05712
C -5.33278 -0.43268 -0.45935
H -5.81841 +0.45296 -0.85657
C +1.36282 +1.15342 -1.37808
C -6.0843 -1.42702 +0.17492
H -7.16136 -1.31851 +0.26948
C -4.06054 -2.70140 +0.56358
H -3.58502 -3.59575 +0.95116
C +3.18666 +5.33099 -2.10391
H +3.90334 +6.08843 -2.4109
C +3.50816 +3.98215 -2.23793
H +4.46660 +3.68309 -2.65415
C -5.44569 -2.55718 +0.68697
H -6.02278 -3.33566 +1.17892
C +1.93592 +5.72708 -1.59312
H +1.69654 +6.78439 -1.51633
C +2.57283 +3.01322 -1.83728
C +2.57646 +1.56848 -1.87686
C +0.98798 +4.78442 -1.19401
H +0.01797 +5.09672 -0.83203
C +1.32411 +3.43315 -1.30294
C -3.77918 -2.6094 -4.45995
H -4.60246 -3.29867 -4.62787
C +0.21638 +3.55485 +3.87016
H +0.62183 +3.15054 +4.79160
C +3.70089 +0.74216 -2.43271
H +4.52763 +1.3879 -2.74633
H +3.39175 +0.15818 -3.30670
H +4.09920 +0.03771 -1.69438
C -0.8159 +5.34708 +2.62031
H -1.18743 +6.36675 +2.56330
C -0.18720 -5.64173 -0.81933
H +0.18143 -6.25225 -1.63931
C +0.09818 +2.71774 +2.76273
H +0.42554 +1.68930 +2.84496
C -0.94780 +4.50262 +1.51688
H -1.45281 +4.86812 +0.62936
C -2.17756 +2.55931 -0.66042
C -3.43647 +2.93992 -2.69938
H -3.46636 +3.00324 -3.78409
C -4.59821 +3.14653 -1.94990
H -5.53793 +3.36473 -2.45054
C -4.54716 +3.06659 -0.55471
H -5.44443 +3.19807 +0.04248
C -2.23012 +2.64723 -2.05944
H -1.33339 +2.48217 -2.64526

C -3.34478 +2.77761 +0.08916
H -3.32088 +2.70509 +1.16995
C -1.29478 -0.25344 +2.20231
O -2.29601 +0.65798 +2.28079
O -1.11459 -1.03099 +3.14282
O -2.76134 +1.44807 +4.73315
H -0.31946 -0.11249 +4.67834
H -4.61465 +0.84891 +1.81190
O -5.31060 +1.33681 +2.27809
H -4.79903 +1.97622 +2.82724
O -0.15929 +0.60349 +5.31979
H -1.01349 +1.08548 +5.26187
H -2.58634 +0.79633 +3.24548
C -3.12978 +2.65525 +4.63718
O -3.85477 +3.18234 +3.76298
O -2.66910 +3.46169 +5.65623
H -2.98804 +4.35855 +5.45056

Ni-TIM_CO2_q-1_s_3H2O_R_MN15
E = -5581.70145933
-1,1
Ni -0.10959 +0.03001 +0.5584
P +1.93569 -0.42524 +1.33885
P -1.39877 -1.57661 -0.11047
P -0.58285 +2.11378 +0.01429
C +2.25334 -2.15277 -2.13215
N +2.59329 -1.22795 -0.07656
N -1.21187 -1.46239 -1.83891
N +0.64276 +2.38123 -1.22150
C +0.7814 -0.10205 -1.34695
C +1.81043 -1.18635 -1.25464
C +3.39196 -2.78977 -1.51361
C +4.27280 -3.79922 -1.93907
H +4.14208 -4.26967 -2.91049
C +5.31953 -4.18423 -1.10425
H +6.00657 -4.96280 -1.42640
C +5.50296 -3.57452 +0.15191
H +6.32661 -3.88926 +0.78748
C +4.64569 -2.56790 +0.59705
H +4.79109 -2.10380 +1.56420
C +3.59762 -2.18154 -0.24324
C +1.76152 -2.46115 -3.51740
H +2.30216 -3.31945 -3.93025
H +1.90742 -1.62049 -4.20745
H +0.69455 -2.70627 -3.53543
C +2.08327 -1.67538 +2.66961
C +2.00068 -3.04653 +2.39654
H +1.90923 -3.39522 +1.37432
C +2.02338 -3.97652 +3.43834
H +1.96392 -5.03619 +3.20755
C +2.10721 -3.54809 +4.76418
H +2.12483 -4.27390 +5.57301
C +2.15803 -2.17908 +5.04613
H +2.20947 -1.83469 +6.07576
C +2.14185 -1.24959 +4.00703
H +2.1781 -0.18922 +4.24009
C +3.27855 +0.78808 +1.73673
C +4.40386 +0.48730 +2.52310
H +4.49590 -0.48099 +3.00224
C +5.41336 +1.43291 +2.71207
H +6.27542 +1.18139 +3.32429
C +5.31937 +2.69378 +2.11604
H +6.10748 +3.42742 +2.26350
C +4.20430 +3.00670 +1.33639
H +4.11274 +3.98449 +0.87116
C +3.19349 +2.06197 +1.15387
H +2.32884 +2.32585 +0.56343
C -0.31042 -0.47046 -2.30283
C -0.61777 -0.15623 -3.60707
C -2.42051 -1.17190 -5.20428
H -2.16293 -0.57381 -6.07486
C -1.72149 -1.00610 -3.99803
C -3.44323 -2.11838 -5.26810
H -3.99138 -2.25631 -6.19672
C +0.07433 +0.77521 -4.55572
H -0.65902 +1.32894 -5.15596
H +0.71311 +0.22626 -5.26338
H +0.70402 +1.50068 -4.03914
C -3.08645 -2.76566 -2.94004
H -3.3349 -3.37241 -2.07539
C -0.36189 +3.43983 +1.27704
C -2.06364 -1.81759 -2.88427
C -1.02097 -3.33177 +0.25736
C -1.10597 -3.78813 +1.58352
H -1.38835 -3.10119 +2.37445
C -0.79229 -5.11266 +1.89089
H -0.86930 -5.45564 +2.91894
C -0.35033 -5.98336 +0.89157
H -0.09342 -7.01054 +1.13648
C -3.22429 -1.42077 +0.09955
C +0.01202 +5.34324 +3.31375
H +0.15981 +6.08054 +4.09848
C -0.56740 -4.20712 -0.74236
H -0.47764 -3.86172 -1.76645
C -3.90378 -0.48320 -0.69558
H -3.36439 +0.09347 -1.43894
C -5.27427 -0.28042 -0.53738
H -5.78079 +0.44626 -1.16532
C +1.38953 +1.23912 -1.61038
C -5.98503 -0.99434 +0.43098
H -7.05189 -0.83024 +0.5579
C -3.94098 -2.1272 +1.07580
H -3.44659 -2.82455 +1.73894
C +3.14780 +5.39657 -2.59113

H +3.84253 +6.14509 -2.96392
C +3.46579 +4.04448 -2.70117
H +4.39931 +3.73427 -3.16383
C -5.31116 -1.91022 +1.24056
H -5.84789 -2.45956 +2.00974
C +1.92900 +5.80675 -2.01744
H +1.69212 +6.86581 -1.95717
C +2.56006 +3.08559 -2.21524
C +2.56730 +1.64198 -2.20228
C +1.01009 +4.87504 -1.53293
H +0.06496 +5.19694 -1.11634
C +1.34328 +3.52088 -1.61894
C -3.77367 -2.90490 -4.1476
H -4.57404 -3.63636 -4.2224
C +0.58881 +4.07474 +3.42419
H +1.18167 +3.81663 +4.29776
C +3.64749 +0.79316 -2.80976
H +4.44305 +1.42452 -3.21941
H +3.26941 +0.17127 -3.62941
H +4.10764 +0.11935 -2.07875
C -0.76922 +5.65374 +2.19814
H -1.23525 +6.63170 +2.11155
C -0.22694 -5.52312 -0.42262
H +0.12833 -6.18998 -1.20399
C +0.39063 +3.12811 +2.41962
H +0.80832 +2.13365 +2.52522
C -0.95886 +4.70670 +1.18911
H -1.58388 +4.95807 +0.33831
C -2.11858 +2.61793 -0.84696
C -3.35216 +3.11767 -2.87796
H -3.36298 +3.26196 -3.95539
C -4.53135 +3.24749 -2.13890
H -5.46499 +3.48932 -2.64040
C -4.50504 +3.05733 -0.75386
H -5.41074 +3.12692 -0.15906
C -2.14971 +2.80958 -2.23665
H -1.23761 +2.72296 -2.81681
C -3.30964 +2.73646 -0.11225
H -3.30788 +2.54623 +0.95483
C -1.12206 +0.07698 +2.20787
O -2.00996 +0.98912 +2.29703
O -0.90925 -0.80770 +3.07622
O -4.17968 +0.61412 +3.72318
H -3.29533 +0.73084 +3.24111
H -2.27408 -1.60387 +3.91682
H -5.14406 +1.84003 +2.80554
O -5.59481 +2.57732 +2.33001
H -4.97879 +3.31884 +2.43906
H -4.01362 +0.96630 +4.61466
O -3.11115 -2.02037 +4.22868
H -3.74666 -1.31111 +4.01181

Ni-TIM_CO2_q-1_s_MN15_6-311++gdp
E = -5352.4934299
-1,1
Ni -0.02291 +0.00823 -0.82380
P -1.52747 -1.57008 -0.64757
P +2.06758 -0.65531 -0.59333
P -0.48592 +2.13255 -0.76382
C -0.18858 -2.01492 +2.94537
N -1.45255 -1.94855 +1.05261
N +2.37648 -0.28920 +1.09826
N -1.04274 +2.30557 +0.89514
C -0.05350 +0.07544 +1.32264
C -0.45709 -1.28307 +1.80665
C -1.11012 -3.12913 +2.95546
C -1.36819 -4.15852 +3.87809
H -0.78544 -4.23736 +4.79251
C -2.38964 -5.06844 +3.61086
H -2.59785 -5.86543 +4.32036
C -3.16499 -4.96792 +2.43819
H -3.95914 -5.68721 +2.25623
C -2.93347 -3.95434 +1.50796
H -3.52560 -3.87188 +0.60209
C -1.90632 -3.04740 +1.78058
C +0.75682 -1.69627 +4.06709
H +0.89576 -2.57405 +4.70789
H +0.38598 -0.88460 +4.70800
H +1.74286 -1.39222 +3.70467
C -1.17783 -3.15829 -1.52092
C -0.73474 -4.31794 -0.86579
H -0.58390 -4.32266 +0.20558
C -0.46189 -5.48142 -1.58810
H -0.12266 -6.36846 -1.05992
C -0.60851 -5.504 -2.97604
H -0.39789 -6.41206 -3.53478
C -0.99725 -4.33938 -3.64301
H -1.08224 -4.33265 -4.72674
C -1.26943 -3.17301 -2.92624
H -1.52404 -2.26080 -3.45641
C -3.34776 -1.32277 -0.83145
C -4.10584 -1.76184 -1.92477
H -3.65527 -2.37049 -2.70031
C -5.46061 -1.42935 -2.02575
H -6.03464 -1.78032 -2.87944
C -6.07482 -0.65631 -1.03825
H -7.12732 -0.39864 -1.12084
C -5.32509 -0.21669 +0.05788
H -5.78939 +0.38707 +0.83340
C -3.97497 -0.54485 +0.15882
H -3.39576 -0.17889 +1.00023
C +1.33236 +0.41900 +1.75158
C +1.86629 +1.21476 +2.74228

C +4.34168 +1.44267 +3.56147
H +4.14472 +2.15010 +4.36325
C +3.29222 +0.97163 +2.75446
C +5.63842 +0.98873 +3.32114
H +6.45681 +1.34855 +3.93999
C +1.15173 +2.08077 +3.73909
H +1.85321 +2.78044 +4.20759
H +0.69550 +1.48999 +4.54633
H +0.35317 +2.67056 +3.28155
C +4.87690 -0.42635 +1.48413
H +5.07635 -1.13630 +0.68810
C -1.86904 +2.85676 -1.74682
C +3.58049 +0.02793 +1.73175
C +2.49850 -2.43126 -0.74748
C +2.37456 -3.04668 -2.00650
H +2.00769 -2.46248 -2.84531
C +2.70289 -4.39313 -2.16363
H +2.61032 -4.85754 -3.14144
C +3.12143 -5.15055 -1.06522
H +3.36664 -6.20208 -1.18902
C +3.50028 +0.20082 -1.38555
C -4.10280 +3.79668 -3.17323
H -4.96710 +4.16202 -3.72203
C +2.90402 -3.19906 +0.35421
H +2.98712 -2.74063 +1.33415
C +3.73037 +1.53406 -1.01353
H +3.07328 +2.01418 -0.29621
C +4.80996 +2.24351 -1.54014
H +4.97659 +3.27057 -1.23000
C -1.06038 +1.12193 +1.67697
C +5.66514 +1.63756 -2.46407
H +6.50439 +2.19074 -2.87751
C +4.36454 -0.40150 -2.30974
H +4.21829 -1.43348 -2.60859
C -3.85787 +4.58599 +2.91859
H -4.60107 +5.13961 +3.48718
C -3.55002 +3.27484 +3.27936
H -4.04026 +2.80935 +4.13075
C +5.43530 +0.31496 -2.85008
H +6.09585 -0.16766 -3.56603
C -3.20713 +5.21031 +1.83783
H -3.45127 +6.23850 +1.58359
C -2.58819 +2.56881 +2.53723
C -2.01278 +1.24852 +2.66826
C -2.24072 +4.53341 +1.09112
H -1.72083 +5.02387 +0.27745
C -1.95325 +3.21221 +1.44044
C +5.90448 +0.06576 +2.29147
H +6.92358 -0.27170 +2.12161
C -4.07040 +2.47918 -2.70829
H -4.90551 +1.80936 -2.89602
C -2.38084 +0.28580 +3.76095
H -3.27517 +0.63665 +4.28725
H -1.58534 +0.18043 +4.50984
H -2.59974 -0.71477 +3.37727
C -3.01433 +4.64071 -2.93711
H -3.02879 +5.66564 -3.29866
C +3.20665 -4.55395 +0.19488
H +3.51750 -5.13946 +1.05638
C -2.95508 +2.01057 -2.01348
H -2.90944 +0.97876 -1.69953
C -1.90594 +4.17590 -2.22551
H -1.07618 +4.85039 -2.04223
C +0.80695 +3.42375 -0.88469
C +2.35186 +5.00791 +0.11051
H +2.74440 +5.50586 +0.99333
C +2.87111 +5.30619 -1.15164
H +3.66889 +6.03681 -1.25462
C +2.36769 +4.65348 -2.28187
H +2.77666 +4.87120 -3.26485
C +1.31950 +4.07533 +0.24520
H +0.91429 +3.85416 +1.22729
C +1.35131 +3.70888 -2.14817
H +0.97492 +3.19146 -3.02789
C -0.02885 -0.13412 -2.77982
O +1.07917 -0.38134 -3.32408
O -1.14561 -0.00255 -3.33349

Ni-TIM_CO2_q-1_s_MN15_6-311++gdp_TS
E = -5352.4781446
-1,1
Ni +0.00037 +0.00798 -0.68902
P -1.45991 -1.52380 -0.62290
P +2.01040 -0.64611 -0.57052
P -0.47708 +2.06393 -0.75757
C -0.31224 -2.01644 +3.07362
N -1.42631 -1.94197 +1.09122
N +2.37811 -0.31053 +1.13246
N -1.04801 +2.29889 +0.91450
C -0.04008 +0.08426 +1.47845
C -0.49097 -1.27242 +1.92215
C -1.22326 -3.13350 +3.00702
C -1.54285 -4.16412 +3.90782
H -1.02311 -4.24102 +4.86005
C -2.54314 -5.07418 +3.56782
H -2.80168 -5.87371 +4.25787
C -3.23199 -4.96835 +2.34289
H -4.01220 -5.68604 +2.10235
C -2.93616 -3.95144 +1.4334
H -3.46813 -3.86783 +0.49147
C -1.92949 -3.04404 +1.77613
C +0.51788 -1.71324 +4.28420
H +1.11240 -2.58821 +4.58036
H -0.10943 -1.45348 +5.14900

H +1.20564 -0.88546 +4.11026
C -1.12685 -3.12710 -1.48642
C -0.66790 -4.29173 -0.85197
H -0.51172 -4.30710 +0.21894
C -0.38829 -5.44309 -1.59191
H -0.03396 -6.33306 -1.07850
C -0.54663 -5.45136 -2.97889
H -0.32961 -6.34979 -3.55051
C -0.95710 -4.28362 -3.62811
H -1.05707 -4.26460 -4.71037
C -1.23187 -3.13135 -2.89026
H -1.52014 -2.22412 -3.41178
C -3.30038 -1.32548 -0.77869
C -4.07368 -1.77763 -1.85683
H -3.63078 -2.38716 -2.63658
C -5.43381 -1.46001 -1.94119
H -6.01587 -1.82361 -2.78439
C -6.04308 -0.68623 -0.95139
H -7.09897 -0.43864 -1.02121
C -5.28106 -0.23440 +0.13165
H -5.74039 +0.36841 +0.91125
C -3.92707 -0.55055 +0.21608
H -3.34225 -0.17674 +1.05128
C +1.36658 +0.38698 +1.85516
C +1.96247 +1.14209 +2.84591
C +4.46598 +1.30821 +3.57292
H +4.30717 +1.99288 +4.40283
C +3.38262 +0.88163 +2.78805
C +5.74551 +0.83731 +3.27405
H +6.59238 +1.16172 +3.87401
C +1.3233 +1.97874 +3.91372
H +1.83744 +2.94423 +4.01250
H +1.38041 +1.49032 +4.89783
H +0.27104 +2.17757 +3.70490
C +4.8889 -0.50873 +1.42505
H +5.04807 -1.20208 +0.60598
C -1.86644 +2.78134 -1.76332
C +3.61088 -0.03526 +1.72668
C +2.48385 -2.41579 -0.73799
C +2.40615 -3.00540 -2.01266
H +2.06268 -2.41528 -2.85864
C +2.75878 -4.34162 -2.19814
H +2.70262 -4.78050 -3.19039
C +3.15338 -5.12200 -1.10665
H +3.41597 -6.16684 -1.24953
C +3.45597 +0.21425 -1.36166
C -4.06573 +3.65962 -3.29669
H -4.91244 +3.99953 -3.88760
C +2.86311 -3.20985 +0.35387
H +2.90738 -2.77497 +1.34717
C +3.68561 +1.54593 -0.97725
H +3.04212 +2.00864 -0.23639
C +4.74426 +2.2756 -1.51660
H +4.90806 +3.29886 -1.19189
C -1.03308 +1.15890 +1.76587
C +5.58334 +1.69588 -2.47272
H +6.40571 +2.26525 -2.89805
C +4.30728 -0.35973 -2.31689
H +4.17322 -1.3897 -2.62753
C -3.87802 +4.64716 +2.84458
H -4.62455 +5.22009 +3.38942
C -3.54777 +3.36140 +3.27137
H -4.02360 +2.93545 +4.15147
C +5.35660 +0.37767 -2.87304
H +6.00458 -0.08783 -3.61177
C -3.24300 +5.22149 +1.72736
H -3.50140 +6.23227 +1.42179
C -2.58247 +2.62916 +2.55899
C -1.98035 +1.33245 +2.76053
C -2.27263 +4.5183 +1.01007
H -1.76351 +4.97591 +0.17134
C -1.96522 +3.21822 +1.42000
C +5.95479 -0.05920 +2.20948
H +6.95996 -0.41228 +1.99356
C -4.03382 +2.35582 -2.79455
H -4.85301 +1.66914 -2.99220
C -2.28435 +0.44794 +3.93599
H -3.14405 +0.83776 +4.49184
H -1.44290 +0.39338 +4.63890
H -2.52139 -0.57817 +3.63977
C -2.99502 +4.52058 -3.04170
H -3.00635 +5.53576 -3.43062
C +3.18836 -4.55694 +0.17007
H +3.47803 -5.16117 +1.02616
C -2.93690 +1.92245 -2.04979
H -2.89680 +0.90006 -1.70212
C -1.90864 +4.08832 -2.27707
H -1.09647 +4.78074 -2.08299
C +0.77653 +3.40416 -0.88548
C +2.27830 +5.04326 +0.09497
H +2.65599 +5.55936 +0.97409
C +2.78896 +5.34785 -1.16915
H +3.56533 +6.10046 -1.27823
C +2.30283 +4.67138 -2.29324
H +2.70383 +4.89329 -3.27881
C +1.27236 +4.08255 +0.23649
H +0.87383 +3.85886 +1.22107
C +1.31488 +3.69837 -2.14982
H +0.95332 +3.16302 -3.02555
C +0.03258 +0.08959 -3.65730
O +1.19708 -0.01404 -3.83614
O -1.12664 +0.18211 -3.86783

Ni-TIM_CO2_q0_d_MN15_6-311++gdp

E = -5352.3417856
0,2
Ni +0.09773 -0.07972 -0.90828
P +0.23038 +2.18375 -0.65531
P -2.11321 -0.83525 -0.64733
P +2.03949 -1.23744 -0.60814
C -1.61921 +1.38350 +2.67912
N -0.56069 +2.31426 +0.88773
N -1.91499 -1.55180 +0.92383
N +2.42361 -0.79640 +1.03085
C +0.03586 -0.08761 +1.26170
C -0.75216 +1.12711 +1.64044
C -1.95824 +2.78416 +2.61059
C -2.76682 +3.61227 +3.40878
H -3.30011 +3.20159 +4.26222
C -2.87096 +4.96456 +3.09508
H -3.49286 +5.61298 +3.70692
C -2.17696 +5.50661 +1.99567
H -2.26968 +6.56561 +1.77008
C -1.36791 +4.70829 +1.18792
H -0.84136 +5.13251 +0.34241
C -1.26750 +3.35028 +1.50409
C -2.0709 +0.45111 +3.76556
H -2.71467 +0.97726 +4.47775
H -1.22673 +0.03592 +4.32857
H -2.63807 -0.40090 +3.37607
C -0.77638 +3.18747 -1.80077
C -2.15673 +3.33635 -1.61101
H -2.63737 +2.90822 -0.74052
C -2.92199 +4.04274 -2.54062
H -3.98900 +4.15718 -2.37457
C -2.32125 +4.59170 -3.67506
H -2.91872 +5.14291 -4.39628
C -0.94889 +4.42469 -3.88237
H -0.47475 +4.84066 -4.76721
C -0.17972 +3.72377 -2.95422
H +0.88325 +3.59845 -3.12867
C +1.78358 +3.14884 -0.43778
C +1.83107 +4.54673 -0.58412
H +0.95776 +5.09570 -0.91753
C +3.00785 +5.24608 -0.31495
H +3.02716 +6.32601 -0.43314
C +4.15402 +4.56396 +0.10379
H +5.06886 +5.11224 +0.31195
C +4.11866 +3.17626 +0.24889
H +5.00219 +2.63101 +0.56879
C +2.94199 +2.47673 -0.02099
H +2.93633 +1.40370 +0.08764
C -0.70170 -1.32609 +1.63144
C -0.56094 -2.28920 +2.60266
C -2.13748 -4.25708 +3.27537
H -1.51650 -4.63570 +4.08324
C -1.72346 -3.14594 +2.52488
C -3.35850 -4.85963 +2.97215
H -3.69020 -5.72186 +3.54497
C +0.47979 -2.42004 +3.67226
H +0.80471 -3.46336 +3.77412
H +0.08009 -2.11540 +4.65040
H +1.36084 -1.80923 +3.47346
C -3.78677 -3.25007 +1.18473
H -4.42077 -2.86146 +0.39521
C +3.48837 -0.92778 -1.68638
C -2.55976 -2.65812 +1.48771
C -3.62856 +0.17434 -0.52389
C -4.11304 +0.80841 -1.67933
H -3.59373 +0.68904 -2.62648
C -5.25243 +1.61102 -1.61428
H -5.62327 +2.09094 -2.51526
C -5.89610 +1.81646 -0.39106
H -6.77410 +2.45443 -0.33887
C -2.56905 -2.30215 -1.65705
C +5.61370 -0.37540 -3.43425
H +6.43932 -0.16100 -4.10741
C -4.28146 +0.37784 +0.70006
H -3.91270 -0.10380 +1.59763
C -1.70236 -3.40411 -1.57597
H -0.82084 -3.35189 -0.94672
C -1.97048 -4.56563 -2.29496
H -1.29269 -5.41087 -2.21777
C +1.45341 -0.01258 +1.72346
C -3.10208 -4.63826 -3.11463
H -3.31008 -5.54398 -3.67809
C -3.70370 -2.38012 -2.47295
H -4.40007 -1.55152 -2.53744
C +5.81446 +0.09364 +3.25555
H +6.67041 +0.36605 +3.86762
C +4.57156 +0.66654 +3.51445
H +4.45213 +1.37700 +4.32812
C -3.96463 -3.54489 -3.20194
H -4.84985 -3.59505 -3.83036
C +5.97477 -0.84721 +2.22064
H +6.94930 -1.29548 +2.04688
C +3.47269 +0.30573 +2.71602
C +2.07777 +0.68181 +2.73746
C +4.89865 -1.22571 +1.41740
H +5.02332 -1.96671 +0.63879
C +3.66085 -0.62784 +1.66273
C -4.17303 -4.36115 +1.93861
H -5.12271 -4.84367 +1.7233
C +4.58809 +0.55743 -3.26494
H +4.60611 +1.49756 -3.80909
C +1.47832 +1.61969 +3.74602
H +2.25509 +2.00349 +4.41517
H +0.72299 +1.12890 +4.36889
H +0.99885 +2.48242 +3.27096
C +5.56842 -1.59347 -2.7494

H +6.35544 -2.32914 -2.88988
C -5.40432 +1.20486 +0.76522
H -5.89711 +1.36530 +1.72026
C +3.52556 +0.27894 -2.40434
H +2.71574 +0.99139 -2.30493
C +4.51069 -1.87147 -1.88417
H +4.47828 -2.82644 -1.36988
C +1.90777 -3.05793 -0.56229
C +1.69257 -5.13312 +0.67463
H +1.67008 -5.65855 +1.62559
C +1.53774 -5.83654 -0.52171
H +1.39058 -6.91305 -0.50655
C +1.57514 -5.15177 -1.74111
H +1.45781 -5.69393 -2.67531
C +1.88119 -3.74932 +0.65654
H +2.01420 -3.21087 +1.58693
C +1.75292 -3.769 -1.76454
H +1.75683 -3.24027 -2.71157
C +0.21554 -0.10832 -2.86758
O +0.08114 -1.34405 -2.89984
O +0.38423 +0.80470 -3.65532

Ni-TIM_COOH2_q-1_d_3H2O_MN15_6-311++Gdp_TS
E = -5582.2566918
-1,2
Ni -0.03557 -0.00706 +0.68902
P +2.15857 -0.65286 +0.76965
P -1.48454 -1.50687 -0.18274
P -0.58317 +2.20007 +0.49078
C +1.63239 -1.6167 -2.96512
N +2.42421 -1.15028 -0.87792
N -1.66549 -0.92596 -1.81349
N +0.43765 +2.65377 -0.86194
C +0.48237 +0.26126 -1.53894
C +1.43011 -0.83692 -1.84404
C +2.81464 -2.40750 -2.72739
C +3.52358 -3.33593 -3.51021
H +3.17296 -3.59719 -4.50566
C +4.68502 -3.91111 -2.99836
H +5.23895 -4.62902 -3.59826
C +5.15636 -3.57095 -1.71591
H +6.06679 -4.02842 -1.33760
C +4.47399 -2.64984 -0.91938
H +4.84038 -2.38879 +0.06577
C +3.30753 -2.07901 -1.43338
C +0.88337 -1.57227 -4.26565
H +1.24924 -2.34826 -4.94659
H +1.00387 -0.60646 -4.77527
H -0.19229 -1.72801 -4.13624
C +2.43026 -2.15403 +1.78508
C +2.22380 -3.44875 +1.28786
H +1.99170 -3.60323 +0.24090
C +2.31455 -4.55404 +2.13659
H +2.16030 -5.54942 +1.73074
C +2.58222 -4.38300 +3.49568
H +2.64601 -5.24537 +4.15367
C +2.75922 -3.09358 +4.00698
H +2.95937 -2.94666 +5.06499
C +2.68380 -1.98757 +3.15991
H +2.82735 -0.99331 +3.57101
C +3.63975 +0.39279 +1.12236
C +4.84871 -0.09875 +1.64266
H +4.94359 -1.1446 +1.91425
C +5.93849 +0.75333 +1.82947
H +6.86614 +0.35676 +2.23371
C +5.84049 +2.10720 +1.49458
H +6.69147 +2.76745 +1.63924
C +4.64223 +2.60781 +0.98099
H +4.54996 +3.65908 +0.72180
C +3.54996 +1.75732 +0.80396
H +2.62147 +2.15812 +0.42263
C -0.81160 +0.12636 -2.24422
C -1.38152 +0.74409 -3.33809
C -3.58034 +0.20253 -4.63556
H -3.48161 +0.9928 -5.37592
C -2.61197 +0.04937 -3.62934
C -4.66195 -0.67697 -4.67311
H -5.41834 -0.56752 -5.44643
C -0.82517 +1.82886 -4.20970
H -0.43557 +1.42207 -5.15493
H -0.01164 +2.37333 -3.72793
H -1.60626 +2.55269 -4.47580
C -3.83064 -1.89089 -2.72313
H -3.92209 -2.69356 -1.99924
C -0.06782 +3.30765 +1.87524
C -2.75611 -1.00058 -2.68280
C -1.09988 -3.28387 -0.41447
C -0.91116 -4.09026 +0.72004
H -0.98923 -3.66755 +1.72185
C -0.59460 -5.44269 +0.57000
H -0.45741 -6.05907 +1.45436
C -0.42136 -5.99292 -0.70207
H -0.15885 -7.04175 -0.81322
C -3.22468 -1.47614 +0.42635
C +0.80468 +4.86175 +4.05459
H +1.14182 +5.46375 +4.89428
C -0.92392 -3.83971 -1.69185
H -1.05325 -3.22411 -2.57494
C -3.96885 -0.30809 +0.18146
H -3.53574 +0.49427 -0.40522
C -5.26298 -0.1727 +0.68081
H -5.82329 +0.73411 +0.47310
C +1.08137 +1.60982 -1.58548

C	-5.83021	-1.19469	+1.44767
H	-6.83833	-1.08899	+1.84021
C	-3.80070	-2.49681	+1.19579
H	-3.25420	-3.41085	+1.39871
C	+2.82631	+5.87798	-1.94181
H	+3.48779	+6.68386	-2.25034
C	+3.08809	+4.57362	-2.35815
H	+3.94327	+4.35956	-2.99434
C	-5.09226	-2.35154	+1.70915
H	-5.51718	-3.14057	+2.32206
C	+1.70624	+6.16940	-1.14079
H	+1.50902	+7.19579	-0.84272
C	+2.22709	+3.53836	-1.95367
C	+2.18199	+2.12639	-2.24128
C	+0.83377	+5.15972	-0.72835
H	-0.03998	+5.39304	-0.13335
C	+1.11630	+3.84928	-1.11914
C	-4.78539	-1.71285	-3.72745
H	-5.63337	-2.39061	-3.77996
C	+1.45146	+3.663	+3.74446
H	+2.29376	+3.32308	+4.34124
C	+3.12859	+1.40811	-3.15968
H	+3.86017	+2.10741	-3.57876
H	+2.60613	+0.93718	-4.00074
H	+3.68827	+0.61821	-2.64428
C	-0.28472	+5.27815	+3.28471
H	-0.79813	+6.20641	+3.52136
C	-0.57690	-5.18443	-1.83222
H	-0.43542	-5.60101	-2.82620
C	+1.00894	+2.88774	+2.67212
H	+1.49875	+1.94701	+2.45502
C	-0.71694	+4.50853	+2.20339
H	-1.56077	+4.85152	+1.61437
C	-2.21416	+2.88272	-0.00243
C	-3.72054	+3.78545	-1.68117
H	-3.88776	+4.13682	-2.69617
C	-4.76854	+3.77778	-0.75752
H	-5.75747	+4.12084	-1.04997
C	-4.53764	+3.32739	+0.54619
H	-5.34823	+3.31784	+1.27038
C	-2.44834	+3.34889	-1.30446
H	-1.63801	+3.37191	-2.02383
C	-3.27328	+2.87295	+0.92344
H	-3.11083	+2.47993	+1.92432
C	-0.26682	-0.11192	+2.57302
O	-2.29500	+0.62869	+2.99978
O	+0.18734	-0.19810	+3.63905
H	-2.73339	+0.30958	+2.19619
O	-2.61149	-1.18349	+4.60819
H	-2.49579	-0.30383	+3.84914
H	-0.01273	-2.62876	+3.78030
H	-4.31554	-1.42764	+4.82097
O	-5.30607	-1.50084	+4.86666
H	-5.58031	-1.04182	+4.05838
H	-2.22034	-0.83731	+5.42724
O	-0.86291	-3.09293	+3.80499
H	-1.51169	-2.38876	+4.08848

Ni-TIM_CO_q-1_d_OH-3H2O_MN15_6-311++Gdp_P
E = -5582.265297
-1,2

Ni	+0.05828	+0.02273	-0.69287
P	-2.10384	+0.69449	-0.91576
P	+0.31276	-2.14142	+0.04001
P	+1.77594	+1.43129	-0.13001
C	-2.58631	-0.61726	+2.72084
N	-2.76620	+0.26088	+0.62686
N	+0.76066	-1.84373	+1.70980
N	+0.99128	+2.32877	+1.14853
C	-0.44414	+0.33540	+1.52448
C	-1.86918	-0.05446	+1.68610
C	-3.97364	-0.62203	+2.32885
C	-5.14830	-1.03664	+2.97988
H	-5.09951	-1.46748	+3.97672
C	-6.37279	-0.88059	+2.33384
H	-7.28745	-1.19653	+2.82925
C	-6.44287	-0.31396	+1.04652
H	-7.40834	-0.20119	+0.56033
C	-5.29187	+0.10795	+0.37939
H	-5.35359	+0.53811	-0.61228
C	-4.06590	-0.04918	+1.03095
C	-2.10508	-1.05105	+4.07366
H	-2.77976	-1.80503	+4.49583
H	-2.07451	-0.21248	+4.78514
H	-1.10229	-1.48154	+4.03990
C	-3.01835	-0.30948	-2.13964
C	-3.52440	-1.57700	-1.81684
H	-3.48676	-1.93499	-0.79488
C	-4.07054	-2.39019	-2.81118
H	-4.46605	-3.36514	-2.54278
C	-4.09259	-1.96392	-4.14104
H	-4.51276	-2.60341	-4.91259
C	-3.56398	-0.71374	-4.47511
H	-3.56847	-0.37565	-5.50783
C	-3.03147	+0.10995	-3.48254
H	-2.62987	+1.08087	-3.75594
C	-2.69081	+2.41662	-1.19427
C	-3.93630	+2.73964	-1.75964
H	-4.59751	+1.95699	-2.11591
C	-4.32980	+4.07254	-1.88636
H	-5.29536	+4.30731	-2.32643
C	-3.48894	+5.10092	-1.44897
H	-3.79981	+6.13754	-1.54782

C -2.24630 +4.79098 -0.89309
H -1.58063 +5.58017 -0.55450
C -1.84972 +3.45809 -0.77293
H -0.88060 +3.22846 -0.35347
C +0.51131 -0.54439 +2.24041
C +1.21235 -0.39638 +3.41828
C +2.75325 -2.04856 +4.70158
H +2.99036 -1.36970 +5.51694
C +1.91750 -1.62723 +3.65425
C +3.25897 -3.34648 +4.68415
H +3.90827 -3.68569 +5.48748
C +1.18789 +0.73600 +4.39970
H +2.18505 +0.89906 +4.82713
H +0.51217 +0.52164 +5.24079
H +0.85791 +1.67218 +3.94752
C +2.08758 -3.83511 +2.59493
H +1.82126 -4.53632 +1.81765
C +2.29161 +2.70110 -1.35791
C +1.60507 -2.52369 +2.59420
C -1.06390 -3.34454 +0.19556
C -1.52114 -3.98982 -0.96669
H -1.04846 -3.77777 -1.92473
C -2.58964 -4.88440 -0.89233
H -2.93504 -5.38357 -1.79395
C -3.2259 -5.12780 +0.32905
H -4.06463 -5.81724 +0.37992
C +1.63641 -3.17204 -0.70711
C +2.96956 +4.56230 -3.35445
H +3.23283 +5.28092 -4.12588
C -1.69305 -3.60319 +1.42060
H -1.33736 -3.12025 +2.32288
C +2.56884 -2.50706 -1.51088
H +2.48145 -1.43940 -1.66135
C +3.57012 -3.21463 -2.18107
H +4.23558 -2.68584 -2.85802
C -0.19924 +1.78310 +1.70459
C +3.64091 -4.60231 -2.05261
H +4.41120 -5.16084 -2.57910
C +1.69212 -4.57457 -0.61672
H +0.94470 -5.11769 -0.04736
C +0.65666 +6.27632 +2.39648
H +0.51409 +7.29728 +2.74194
C -0.31897 +5.31640 +2.66048
H -1.21577 +5.58086 +3.21517
C +2.69700 -5.28246 -1.27350
H +2.73493 -6.36555 -1.18944
C +1.83149 +5.93892 +1.69849
H +2.58654 +6.69894 +1.51548
C -0.12577 +3.99902 +2.20893
C -0.90067 +2.78983 +2.34083
C +2.05035 +4.63719 +1.24258
H +2.96396 +4.38027 +0.72071
C +1.05842 +3.68402 +1.48458
C +2.92275 -4.23047 +3.64224
H +3.31032 -5.24594 +3.65218
C +1.64669 +4.13506 -3.21444
H +0.87364 +4.51765 -3.87542
C -2.18746 +2.68325 +3.10866
H -2.48174 +3.66365 +3.49809
H -2.10072 +2.00365 +3.96509
H -3.01295 +2.31718 +2.48767
C +3.95433 +4.05404 -2.50315
H +4.98652 +4.37759 -2.60774
C -2.77591 -4.48355 +1.48441
H -3.26135 -4.66909 +2.43915
C +1.31322 +3.20217 -2.23211
H +0.28862 +2.85958 -2.14966
C +3.61909 +3.13132 -1.51087
H +4.39479 +2.75394 -0.85319
C +3.35001 +0.89719 +0.64880
C +4.80933 +0.69379 +2.57767
H +5.02294 +0.92277 +3.61851
C +5.68995 -0.09866 +1.83716
H +6.58986 -0.49397 +2.30111
C +5.41282 -0.37304 +0.49452
H +6.10096 -0.97586 -0.09284
C +3.64664 +1.19330 +1.98667
H +2.96864 +1.80665 +2.56835
C +4.25174 +0.12245 -0.10149
H +4.04996 -0.06563 -1.15229
C +0.26465 +0.10536 -2.51133
O +3.21042 +0.13240 -3.14248
O +0.12218 +0.50280 -3.58700
H +2.84521 +1.00619 -3.34636
O +2.03690 -1.62195 -4.58259
H +2.64590 -0.55239 -3.70019
H -0.84466 -1.97729 -3.82843
H +3.71322 -1.77324 -4.93295
O +4.69712 -1.57881 -4.91595
H +4.66384 -0.80093 -4.32906
H +1.67915 -1.08293 -5.30760
O -0.22484 -2.71671 -3.73906
H +0.67034 -2.31978 -4.01996

Ni-TIM_CO_q-1_s_MN15_6-311++gdp
E = -5277.2756292
-1,1
Ni +0.02280 +0.03194 -1.30097
P -2.06726 +0.37386 -0.55052
P +0.70649 -1.92134 -0.56354
P +1.35483 +1.63983 -0.56485
C -1.75592 -1.59938 +2.79888
N -2.37669 -0.21217 +1.07975
N +1.34727 -1.97901 +1.07005

N	+1.07753	+2.15446	+1.11204
C	+0.01762	-0.00931	+1.81740
C	-1.28112	-0.644	+1.90831
C	-3.17048	-1.72169	+2.60088
C	-4.16740	-2.46352	+3.26175
H	-3.89498	-3.16444	+4.04749
C	-5.50306	-2.27963	+2.90448
H	-6.27905	-2.84846	+3.41162
C	-5.86138	-1.35959	+1.90297
H	-6.90802	-1.22238	+1.64382
C	-4.88699	-0.61056	+1.23329
H	-5.17088	+0.09923	+0.46663
C	-3.54946	-0.80982	+1.57330
C	-0.98379	-2.24059	+3.91197
H	-1.62524	-2.41234	+4.78595
H	-0.14654	-1.60793	+4.22527
H	-0.55997	-3.21612	+3.63222
C	-3.19253	-0.56507	-1.67265
C	-3.72819	-1.83025	-1.40761
H	-3.54905	-2.31229	-0.45567
C	-4.48982	-2.49510	-2.37423
H	-4.90186	-3.47348	-2.14345
C	-4.70406	-1.92093	-3.62673
H	-5.29499	-2.44198	-4.37531
C	-4.14002	-0.67323	-3.91658
H	-4.28552	-0.21920	-4.89335
C	-3.39041	-0.00524	-2.95078
H	-2.95721	+0.96272	-3.18919
C	-2.91327	+2.02677	-0.49379
C	-4.25236	+2.24864	-0.85725
H	-4.85551	+1.43798	-1.25141
C	-4.82713	+3.51475	-0.72798
H	-5.86457	+3.66375	-1.01611
C	-4.07699	+4.58318	-0.22784
H	-4.52773	+5.56689	-0.12593
C	-2.74350	+4.37831	+0.13117
H	-2.14133	+5.19759	+0.51480
C	-2.17063	+3.11363	-0.00988
H	-1.13209	+2.97369	+0.24425
C	+1.20466	-0.83065	+1.91802
C	+2.27101	-0.80487	+2.80754
C	+4.20077	-2.50846	+3.21188
H	+4.68322	-1.95776	+4.01624
C	+3.06378	-1.97828	+2.57501
C	+4.69199	-3.74928	+2.80366
H	+5.57142	-4.16590	+3.28952
C	+2.47904	+0.15968	+3.93494
H	+3.38425	+0.76957	+3.79910
H	+2.59699	-0.36431	+4.89442
H	+1.63318	+0.84705	+4.02693
C	+2.92405	-3.97042	+1.13435
H	+2.43350	-4.53435	+0.34815
C	+1.20726	+3.15972	-1.6329

C	+2.44793	-2.72025	+1.52635
C	-0.45556	-3.34714	-0.56481
C	-1.18136	-3.63360	-1.73153
H	-1.08275	-2.99057	-2.60257
C	-2.04906	-4.72645	-1.77755
H	-2.59647	-4.93823	-2.69142
C	-2.24133	-5.51832	-0.64347
H	-2.93150	-6.35733	-0.67398
C	+2.13196	-2.56291	-1.56388
C	+0.86278	+5.35012	-3.38020
H	+0.73068	+6.19577	-4.05007
C	-0.65316	-4.14923	+0.56973
H	-0.10661	-3.93550	+1.48129
C	+3.40102	-1.99678	-1.35781
H	+3.54383	-1.26096	-0.57535
C	+4.49198	-2.38824	-2.13292
H	+5.46447	-1.94245	-1.94622
C	+0.12138	+1.42444	+1.91082
C	+4.33416	-3.34091	-3.14318
H	+5.18412	-3.64337	-3.74922
C	+1.98315	-3.51883	-2.58008
H	+1.02304	-3.98794	-2.76144
C	+0.66612	+5.93637	+2.83220
H	+0.50881	+6.90391	+3.30351
C	-0.16197	+4.86343	+3.16568
H	-0.95377	+4.98181	+3.90160
C	+3.07499	-3.90088	-3.36510
H	+2.93758	-4.64535	-4.14527
C	+1.71722	+5.776	+1.91305
H	+2.37181	+6.61362	+1.68653
C	+0.04924	+3.61549	+2.55036
C	-0.53115	+2.32119	+2.75062
C	+1.94437	+4.54134	+1.29081
H	+2.78371	+4.41301	+0.62076
C	+1.08468	+3.48421	+1.57888
C	+4.06015	-4.47554	+1.77765
H	+4.45315	-5.44434	+1.48028
C	-0.17717	+4.43934	-3.18185
H	-1.12466	+4.56561	-3.69931
C	-1.56894	+1.98656	+3.77749
H	-1.50128	+2.65884	+4.64231
H	-1.45113	+0.95875	+4.13551
H	-2.59216	+2.07322	+3.38233
C	+2.08261	+5.15397	-2.72754
H	+2.90641	+5.84422	-2.88962
C	-1.54986	-5.21852	+0.53416
H	-1.7018	-5.82162	+1.42571
C	+0.00136	+3.35094	-2.32703
H	-0.79321	+2.62423	-2.21338
C	+2.25295	+4.06959	-1.86512
H	+3.21708	+3.92675	-1.39033
C	+3.17499	+1.40354	-0.58192
C	+5.29191	+1.01806	+0.54344

H +5.85632 +0.92270 +1.46760
C +5.93298 +0.87738 -0.68960
H +6.99929 +0.67159 -0.73144
C +5.19515 +1.00235 -1.87177
H +5.68611 +0.89154 -2.83495
C +3.92189 +1.28985 +0.59681
H +3.42933 +1.40943 +1.55432
C +3.82454 +1.25058 -1.81852
H +3.25472 +1.32690 -2.74171
C +0.03081 +0.00737 -3.03033
O +0.08701 -0.03625 -4.19951

Ni-TIM_CO_q0_d_MN15_6-311++gdp
E = -5277.1651993
0,2
Ni -0.03179 -0.02905 -0.98218
P +2.21552 +0.23246 -0.61887
P -1.31817 +1.78924 -0.62382
P -0.88501 -2.09312 -0.63772
C +1.11779 +1.91793 +2.71234
N +2.22324 +0.99266 +0.94342
N -1.96112 +1.49063 +0.95726
N -0.31339 -2.44919 +0.97420
C -0.03081 -0.00120 +1.34082
C +1.01277 +0.99282 +1.69479
C +2.44282 +2.48338 +2.64113
C +3.12485 +3.42669 +3.42949
H +2.62734 +3.89972 +4.27231
C +4.44765 +3.73829 +3.12299
H +4.98353 +4.46540 +3.72801
C +5.10480 +3.11802 +2.04301
H +6.13858 +3.37200 +1.82422
C +4.45160 +2.17632 +1.24666
H +4.96066 +1.70218 +0.41681
C +3.12305 +1.87188 +1.55240
C +0.13002 +2.21417 +3.80287
H +0.40166 +3.13842 +4.32460
H +0.09948 +1.41320 +4.55583
H -0.88790 +2.33579 +3.42365
C +2.93945 +1.42060 -1.80644
C +3.01069 +2.80222 -1.59168
H +2.70771 +3.22656 -0.64284
C +3.46870 +3.64817 -2.60562
H +3.52575 +4.71681 -2.41987
C +3.83592 +3.12994 -3.84816
H +4.18969 +3.79209 -4.63373
C +3.73621 +1.75393 -4.08055
H +4.00869 +1.34110 -5.04817
C +3.28865 +0.90515 -3.06944
H +3.21896 -0.16324 -3.25969
C +3.50891 -1.07393 -0.48706
C +4.86929 -0.84745 -0.75695
H +5.20347 +0.12130 -1.11359
C +5.80457 -1.86895 -0.58532
H +6.85308 -1.67779 -0.79776
C +5.39650 -3.13061 -0.1415
H +6.12745 -3.92356 -0.00753
C +4.04536 -3.37004 +0.11712
H +3.71220 -4.34876 +0.45157
C +3.10964 -2.35031 -0.06180
H +2.06398 -2.55645 +0.11278
C -1.40452 +0.41477 +1.70337
C -2.2969 +0.04634 +2.68765
C -4.62417 +1.03947 +3.32461
H -4.81952 +0.36405 +4.15380
C -3.43962 +0.92191 +2.57926
C -5.53731 +2.03864 +2.98911
H -6.45849 +2.13938 +3.55759
C -2.13155 -0.95995 +3.78565
H -3.03208 -1.57979 +3.88758
H -1.9759 -0.46727 +4.75646
H -1.28304 -1.62295 +3.61174
C -4.10454 +2.83606 +1.17784
H -3.90413 +3.52710 +0.36647
C -0.24279 -3.40506 -1.75986
C -3.20016 +1.82595 +1.51017
C -0.59926 +3.46503 -0.52426
C +0.00890 +4.00043 -1.67097
H +0.03953 +3.42244 -2.59157
C +0.58523 +5.27098 -1.63275
H +1.04654 +5.67878 -2.52736
C +0.59416 +6.00090 -0.44137
H +1.0581 +6.98294 -0.40849
C -2.82549 +1.97600 -1.65668
C +0.82402 -5.28098 -3.56425
H +1.23617 -6.00799 -4.25887
C -0.59315 +4.20407 +0.66816
H -1.05304 +3.79575 +1.56133
C -3.72004 +0.89332 -1.65138
H -3.52155 +0.03635 -1.01740
C -4.86983 +0.92173 -2.43759
H -5.55482 +0.07893 -2.41517
C +0.31798 -1.39346 +1.69471
C -5.13572 +2.02878 -3.25026
H -6.03044 +2.05081 -3.86660
C -3.09685 +3.08398 -2.47062
H -2.42754 +3.93751 -2.48337
C +1.24099 -5.73737 +2.98276
H +1.68438 -6.56174 +3.53556
C +1.60604 -4.42716 +3.28782
H +2.32291 -4.22433 +4.07932
C -4.24687 +3.10538 -3.26534
H -4.44857 +3.97095 -3.89084
C +0.29179 -6.00792 +1.97939

H +0.00583 -7.03555 +1.77149
C +1.02751 -3.36978 +2.56497
C +1.14880 -1.93432 +2.65391
C -0.30068 -4.97474 +1.25036
H -1.05057 -5.18671 +0.49939
C +0.09343 -3.66629 +1.53446
C -5.27945 +2.92734 +1.92814
H -6.00234 +3.70277 +1.68895
C +1.49629 -4.08055 -3.32400
H +2.43245 -3.86413 -3.83154
C +1.96055 -1.20508 +3.6856
H +2.48489 -1.91597 +4.33274
H +1.33188 -0.57747 +4.32908
H +2.71568 -0.55129 +3.23475
C -0.38851 -5.53819 -2.91809
H -0.92351 -6.46465 -3.10865
C +0.01183 +5.46138 +0.70949
H +0.02235 +6.02142 +1.64074
C +0.96002 -3.14576 -2.43773
H +1.47602 -2.20723 -2.27167
C -0.91978 -4.60733 -2.02482
H -1.86805 -4.81866 -1.54171
C -2.67308 -2.45525 -0.54293
C -4.71909 -2.74184 +0.72873
H -5.21866 -2.84099 +1.68885
C -5.46026 -2.76840 -0.45493
H -6.53996 -2.88573 -0.42059
C -4.80751 -2.64616 -1.68625
H -5.37704 -2.66956 -2.61124
C -3.33091 -2.59215 +0.68680
H -2.75992 -2.58104 +1.60794
C -3.42337 -2.48202 -1.73135
H -2.92589 -2.36654 -2.69127
C -0.12285 +0.04605 -2.79032
O -0.40280 +0.15463 -3.90608

Ni-TIM_CO_q0_s_OH-3H2O_MN15_6-311++Gdp_P
E = -5582.1410365
0,1
Ni -0.00432 -0.01910 +0.61703
P -2.19556 -0.61493 +0.80769
P +0.42105 +2.14388 +0.05947
P +1.69583 -1.55255 +0.29910
C -2.28392 +0.84218 -2.78578
N -2.72229 -0.06003 -0.74091
N +0.94080 +1.84535 -1.56599
N +0.90612 -2.38071 -1.02008
C -0.33187 -0.25976 -1.38562
C -1.71883 +0.23564 -1.68893
C -3.70731 +0.89631 -2.53824
C -4.78777 +1.37561 -3.29863
H -4.62005 +1.81327 -4.27891
C -6.07488 +1.27643 -2.77927
H -6.91874 +1.64194 -3.35834
C -6.30192 +0.70482 -1.51142
H -7.31605 +0.63873 -1.12701
C -5.24965 +0.21984 -0.73724
H -5.42942 -0.21294 +0.23884
C -3.95939 +0.31711 -1.26639
C -1.64014 +1.29216 -4.06435
H -2.36535 +1.82827 -4.68427
H -1.26701 +0.44476 -4.65186
H -0.79288 +1.96143 -3.89393
C -3.11292 +0.36311 +2.04198
C -3.61753 +1.63489 +1.74183
H -3.54346 +2.03070 +0.73671
C -4.21259 +2.40378 +2.74342
H -4.60537 +3.38548 +2.49711
C -4.28465 +1.92389 +4.05222
H -4.74157 +2.52925 +4.83014
C -3.76360 +0.66338 +4.36084
H -3.81264 +0.28437 +5.37769
C -3.18310 -0.11732 +3.36173
H -2.79290 -1.09971 +3.61080
C -2.84321 -2.32138 +0.99040
C -4.16094 -2.57643 +1.40975
H -4.8134 -1.76047 +1.70022
C -4.63656 -3.88603 +1.47185
H -5.65695 -4.07088 +1.79589
C -3.80577 -4.95454 +1.12074
H -4.18014 -5.97334 +1.17112
C -2.49300 -4.71027 +0.71351
H -1.83607 -5.53273 +0.44542
C -2.01457 -3.40119 +0.65107
H -0.99415 -3.22629 +0.34570
C +0.70372 +0.56069 -2.10263
C +1.41887 +0.40886 -3.26597
C +2.99722 +2.08162 -4.49501
H +3.24265 +1.42186 -5.32298
C +2.13287 +1.64896 -3.47699
C +3.52611 +3.36830 -4.42496
H +4.19765 +3.71861 -5.20432
C +1.43468 -0.71155 -4.25891
H +0.91628 -0.41619 -5.18130
H +0.95385 -1.61339 -3.88105
H +2.46484 -0.96200 -4.54013
C +2.33434 +3.82464 -2.33905
H +2.07609 +4.49301 -1.52568
C +1.93546 -2.82004 +1.60755
C +1.81353 +2.53001 -2.41218
C -0.90359 +3.39128 -0.08770
C -1.47923 +3.91864 +1.08033
H -1.14249 +3.58215 +2.06233

C	-2.50043	+4.86582	+0.97759
H	-2.93903	+5.27716	+1.88262
C	-2.97347	+5.26685	-0.27446
H	-3.77794	+5.99403	-0.34629
C	+1.85435	+3.01647	+0.78712
C	+2.21987	-4.72779	+3.65184
H	+2.33030	-5.46524	+4.44212
C	-1.37089	+3.80222	-1.34507
H	-0.92684	+3.40304	-2.24859
C	+3.08053	+2.33999	+0.73861
H	+3.13513	+1.36073	+0.28261
C	+4.23048	+2.92128	+1.26585
H	+5.17235	+2.38156	+1.22142
C	-0.19365	-1.73504	-1.62773
C	+4.16452	+4.18776	+1.85455
H	+5.05777	+4.64118	+2.27528
C	+1.79107	+4.29138	+1.36559
H	+0.85629	+4.83844	+1.40191
C	+0.41882	-6.21729	-2.51510
H	+0.24111	-7.20620	-2.92922
C	-0.4562	-5.17690	-2.8141
H	-1.31240	-5.34452	-3.46164
C	+2.94553	+4.86967	+1.90064
H	+2.88893	+5.85604	+2.35256
C	+1.54229	-6.00343	-1.69182
H	+2.21920	-6.82777	-1.48421
C	-0.21271	-3.90417	-2.27020
C	-0.90643	-2.64026	-2.38081
C	+1.80925	-4.75099	-1.14162
H	+2.6835	-4.59041	-0.52312
C	+0.91541	-3.71504	-1.42784
C	+3.19599	+4.22844	-3.35894
H	+3.61636	+5.23008	-3.32995
C	+0.99330	-4.08928	+3.45464
H	+0.14281	-4.32515	+4.08821
C	-2.14878	-2.42928	-3.19608
H	-2.47198	-3.37435	-3.64338
H	-1.99242	-1.71559	-4.01190
H	-2.97775	-2.05604	-2.58572
C	+3.30522	-4.40931	+2.83240
H	+4.26374	-4.89924	+2.97962
C	-2.41048	+4.72904	-1.43500
H	-2.77384	+5.03357	-2.41275
C	+0.85489	-3.13586	+2.44624
H	-0.10031	-2.64294	+2.31787
C	+3.16578	-3.46342	+1.81565
H	+4.01798	-3.23480	+1.18549
C	+3.37346	-1.24198	-0.38136
C	+4.90408	-1.22768	-2.26750
H	+5.09756	-1.43930	-3.31566
C	+5.90416	-0.67798	-1.46318
H	+6.88085	-0.45306	-1.88328
C	+5.64400	-0.42599	-0.11323
H	+6.41975	-0.00845	+0.52333
C	+3.64699	-1.51293	-1.73104
H	+2.88805	-1.95243	-2.36479
C	+4.38907	-0.70422	+0.43021
H	+4.17859	-0.49148	+1.47421
C	+0.10469	+0.00921	+2.40860
O	+2.81183	-0.10721	+2.9407
O	-0.05241	-0.16927	+3.53059
H	+2.52894	-0.80617	+3.55123
O	+1.97829	+2.03543	+4.00417
H	+2.41697	+0.78671	+3.34907
H	+4.42401	+0.71453	+4.29716
H	+0.42524	+2.45762	+3.96673
O	-0.53759	+2.78237	+3.88336
H	-1.07887	+1.99991	+4.06422
H	+2.32108	+2.69677	+3.38474
O	+4.42306	+1.42843	+4.95700
H	+3.50199	+1.77699	+4.79491

Ni-TIM_CO_q1_s_MN15_6-311++gdp

E = -5277.038486

1,1

Ni	-0.03623	-0.02050	-0.84332
P	+2.23205	+0.08095	-0.62529
P	-1.17161	+1.90407	-0.61506
P	-1.03401	-2.02767	-0.65776
C	+1.18912	+1.78080	+2.69802
N	+2.27531	+0.83887	+0.92787
N	-1.86272	+1.62694	+0.93544
N	-0.51752	-2.42601	+0.95183
C	-0.04824	-0.00646	+1.20261
C	+1.05209	+0.92455	+1.63166
C	+2.5696	+2.21250	+2.70376
C	+3.30452	+3.04577	+3.56478
H	+2.82295	+3.51941	+4.41578
C	+4.65924	+3.24483	+3.31695
H	+5.23888	+3.88430	+3.97731
C	+5.29513	+2.62311	+2.22349
H	+6.35530	+2.79027	+2.05407
C	+4.59123	+1.79226	+1.35438
H	+5.08216	+1.31577	+0.51435
C	+3.23015	+1.59836	+1.60723
C	+0.18312	+2.15119	+3.74819
H	+0.58916	+2.92659	+4.40519
H	-0.08555	+1.29428	+4.37784
H	-0.74438	+2.53735	+3.31677
C	+3.00626	+1.19964	-1.84014
C	+3.33267	+2.53562	-1.58591
H	+3.16272	+2.96840	-0.60867
C	+3.87139	+3.32681	-2.60448
H	+4.12717	+4.36136	-2.39533

C +4.06414 +2.79963 -3.88151
H +4.47900 +3.42068 -4.67058
C +3.71414 +1.47091 -4.14653
H +3.85275 +1.05568 -5.14080
C +3.18819 +0.67303 -3.13259
H +2.92649 -0.36137 -3.34339
C +3.40565 -1.32364 -0.51239
C +4.76664 -1.18101 -0.83025
H +5.14581 -0.23751 -1.20848
C +5.64104 -2.25644 -0.67027
H +6.69195 -2.13465 -0.91745
C +5.16783 -3.48346 -0.19645
H +5.85166 -4.31886 -0.07384
C +3.81350 -3.63623 +0.10911
H +3.43313 -4.58895 +0.46653
C +2.93690 -2.56362 -0.05242
H +1.88758 -2.70024 +0.16349
C -1.39510 +0.49466 +1.63816
C -2.27472 +0.18318 +2.64592
C -4.48291 +1.37750 +3.36810
H -4.70748 +0.71788 +4.20185
C -3.33396 +1.17077 +2.58852
C -5.32194 +2.44551 +3.05698
H -6.21539 +2.61853 +3.65090
C -2.20974 -0.87488 +3.70429
H -3.21483 -1.25967 +3.91306
H -1.82044 -0.46596 +4.64695
H -1.57762 -1.71733 +3.42485
C -3.88744 +3.13290 +1.20144
H -3.65557 +3.80346 +0.38104
C -0.46092 -3.34771 -1.79087
C -3.05497 +2.05842 +1.51944
C -0.29432 +3.49196 -0.51728
C +0.45261 +3.91169 -1.62803
H +0.51274 +3.28642 -2.51493
C +1.13219 +5.12967 -1.58917
H +1.70683 +5.44996 -2.45265
C +1.09938 +5.91345 -0.43325
H +1.64226 +6.85394 -0.39974
C -2.61835 +2.16493 -1.70045
C +0.48921 -5.31687 -3.55134
H +0.85697 -6.08082 -4.23080
C -0.32984 +4.28104 +0.64228
H -0.90239 +3.95958 +1.50500
C -3.65763 +1.22400 -1.61676
H -3.59735 +0.40918 -0.90399
C -4.77970 +1.34795 -2.43352
H -5.58092 +0.61946 -2.35499
C +0.20224 -1.41999 +1.64022
C -4.86934 +2.40239 -3.34717
H -5.74384 +2.49639 -3.98510
C -2.71113 +3.22363 -2.61426
H -1.92392 +3.96651 -2.68402
C +0.63546 -5.75686 +3.13649
H +0.96949 -6.59546 +3.74149
C +1.10653 -4.47635 +3.41380
H +1.79923 -4.30830 +4.23373
C -3.83471 +3.33586 -3.43669
H -3.90112 +4.15961 -4.14198
C -0.28155 -5.98032 +2.09107
H -0.64635 -6.98648 +1.90378
C +0.6657 -3.40318 +2.62180
C +0.94160 -1.98162 +2.65161
C -0.74038 -4.93190 +1.29473
H -1.46342 -5.10397 +0.50777
C -0.24799 -3.65377 +1.56459
C -5.02690 +3.31203 +1.98608
H -5.69602 +4.13957 +1.76695
C +1.24734 -4.16864 -3.31255
H +2.20569 -4.03007 -3.80492
C +1.84120 -1.31436 +3.65017
H +2.27318 -2.05728 +4.32754
H +1.30345 -0.58210 +4.26096
H +2.67187 -0.79082 +3.16502
C -0.74962 -5.47620 -2.92400
H -1.34883 -6.36207 -3.11449
C +0.37468 +5.48453 +0.68268
H +0.35330 +6.08840 +1.58553
C +0.77166 -3.18666 -2.44394
H +1.36586 -2.29755 -2.27454
C -1.22608 -4.49756 -2.05196
H -2.19608 -4.62805 -1.58414
C -2.84405 -2.19230 -0.63432
C -4.95008 -2.27655 +0.55846
H -5.49399 -2.32746 +1.49761
C -5.64174 -2.22984 -0.65400
H -6.72815 -2.24137 -0.66173
C -4.93485 -2.17039 -1.85985
H -5.46902 -2.13551 -2.80494
C -3.55365 -2.26354 +0.57186
H -3.02097 -2.31502 +1.51378
C -3.54136 -2.14164 -1.85309
H -2.99773 -2.07417 -2.79203
C -0.14166 +0.04725 -2.61677
O -0.27605 +0.12384 -3.75458

Ni-TIM_MeCN_q0_d_MN15_6-311++Gdp_forall
E = -5296.5622346
0,2
Ni -0.00516 +0.01369 +0.81406
P +2.16192 +0.55051 +0.46201
P -0.64613 -2.11985 +0.53011
P -1.52687 +1.65948 +0.67474
C +1.56436 -1.35970 -2.87897

N	+2.37377	-0.14340	-1.12554
N	-1.39031	-2.02824	-1.03713
N	-1.14998	+2.25860	-0.93065
N	+0.18625	-0.19367	+2.84703
C	-0.09861	+0.04843	-1.41780
C	+1.20272	-0.52680	-1.83979
C	+3.00099	-1.47936	-2.85139
C	+3.92297	-2.15484	-3.67005
H	+3.57659	-2.75403	-4.50850
C	+5.28512	-2.04149	-3.39882
H	+6.00425	-2.56005	-4.02815
C	+5.74435	-1.25898	-2.32250
H	+6.81106	-1.18188	-2.12896
C	+4.85074	-0.57440	-1.49665
H	+5.21074	+0.02605	-0.67077
C	+3.48534	-0.69248	-1.76802
C	+0.67913	-1.94331	-3.94212
H	+1.25426	-2.60211	-4.60159
H	+0.22476	-1.16592	-4.57168
H	-0.14463	-2.53171	-3.52534
C	+3.31810	-0.34141	+1.56411
C	+3.67909	-1.67894	+1.34927
H	+3.37279	-2.18725	+0.44275
C	+4.43050	-2.36849	+2.30308
H	+4.71046	-3.40082	+2.11485
C	+4.80945	-1.74336	+3.49342
H	+5.3924	-2.2845	+4.23376
C	+4.42749	-0.41894	+3.72911
H	+4.70644	+0.07463	+4.65623
C	+3.68529	+0.27617	+2.77412
H	+3.39737	+1.30552	+2.96933
C	+2.93285	+2.22169	+0.30498
C	+4.31015	+2.46555	+0.44409
H	+4.98385	+1.65832	+0.71094
C	+4.82565	+3.74949	+0.26153
H	+5.89314	+3.92065	+0.37275
C	+3.97474	+4.81074	-0.06338
H	+4.37891	+5.80945	-0.20596
C	+2.60349	+4.58270	-0.19379
H	+1.92934	+5.39936	-0.43739
C	+2.08899	+3.29908	-0.00413
H	+1.02395	+3.13881	-0.08465
C	-1.25955	-0.80685	-1.75977
C	-2.25960	-0.74418	-2.70725
C	-4.12991	-2.45924	-3.31921
H	-4.57421	-1.87269	-4.11967
C	-3.03113	-1.95930	-2.60120
C	-4.63544	-3.71766	-2.99272
H	-5.48663	-4.11463	-3.54037
C	-2.48436	+0.28254	-3.77530
H	-3.54726	+0.54861	-3.84661
H	-2.18712	-0.09831	-4.76379
H	-1.91902	+1.19767	-3.59263
C	-2.95372	-4.01580	-1.24592
H	-2.49994	-4.61285	-0.46205
C	-1.31316	+3.06173	+1.85730
C	-2.45883	-2.75139	-1.56994
C	+0.52302	-3.52201	+0.38249
C	+1.29572	-3.87186	+1.50273
H	+1.17514	-3.33044	+2.43762
C	+2.23532	-4.90043	+1.41976
H	+2.81976	-5.16568	+2.29610
C	+2.44319	-5.56462	+0.20794
H	+3.18687	-6.35403	+0.13966
C	-2.00730	-2.76208	+1.59188
C	-0.84716	+5.07513	+3.76880
H	-0.66827	+5.85509	+4.50409
C	+0.73370	-4.19624	-0.82987
H	+0.15031	-3.93098	-1.70475
C	-3.22055	-2.05478	+1.54916
H	-3.32866	-1.21843	+0.86698
C	-4.28983	-2.42905	+2.36031
H	-5.22252	-1.87522	+2.30462
C	-0.255	+1.48706	-1.72777
C	-4.15850	-3.50718	+3.24186
H	-4.99017	-3.79694	+3.87844
C	-1.88281	-3.84328	+2.47542
H	-0.96324	-4.41642	+2.52133
C	-0.85178	+5.93701	-2.83961
H	-0.72928	+6.87791	-3.37032
C	-0.10594	+4.82470	-3.22638
H	+0.59101	+4.89014	-4.05809
C	-2.95281	-4.20871	+3.29797
H	-2.84184	-5.05000	+3.97717
C	-1.77323	+5.85479	-1.77879
H	-2.35829	+6.72865	-1.50434
C	-0.27046	+3.61364	-2.53231
C	+0.29912	+2.29928	-2.69695
C	-1.95957	+4.66162	-1.07718
H	-2.68900	+4.59836	-0.28020
C	-1.18854	+3.55773	-1.44597
C	-4.05335	-4.48732	-1.96752
H	-4.46062	-5.46759	-1.73419
C	+0.16469	+4.16782	+3.44677
H	+1.13514	+4.23355	+3.93146
C	+1.24368	+1.90269	-3.79517
H	+1.42145	+2.74521	-4.47216
H	+0.84578	+1.07639	-4.39563
H	+2.21888	+1.57994	-3.41043
C	-2.09678	+4.96737	+3.15134
H	-2.89299	+5.66212	+3.40498
C	+1.69617	-5.20407	-0.91727
H	+1.85711	-5.71175	-1.86473
C	-0.07161	+3.16214	+2.50852
H	+0.71042	+2.44647	+2.28040
C	-2.32899	+3.96780	+2.20591

H -3.30860 +3.89145 +1.74560
C -3.34864 +1.46867 +0.58996
C -5.40208 +1.18648 -0.67637
H -5.91786 +1.17963 -1.63314
C -6.10541 +0.92721 +0.50231
H -7.17048 +0.71437 +0.46820
C -5.43211 +0.94451 +1.72834
H -5.97188 +0.74673 +2.65053
C -4.03321 +1.46213 -0.63401
H -3.49778 +1.67045 -1.55346
C +0.56458 -0.58466 +3.87170
C +1.05891 -1.09668 +5.13851
H +0.30719 -1.74759 +5.59639
H +1.28052 -0.26689 +5.81716
H +1.97647 -1.66887 +4.96469
C -4.06257 +1.20482 +1.77167
H -3.54541 +1.19855 +2.72795

Ni-TIM_MeCN_q0_d_MN15_6-311++gdp
E = -5296.5622346
0,2
Ni -0.00516 +0.01369 +0.81406
P +2.16192 +0.55051 +0.46201
P -0.64613 -2.11985 +0.53011
P -1.52687 +1.65948 +0.67474
C +1.56436 -1.35970 -2.87897
N +2.37377 -0.14340 -1.12554
N -1.39031 -2.02824 -1.03713
N -1.14998 +2.25860 -0.93065
N +0.18625 -0.19367 +2.84703
C -0.09861 +0.04843 -1.41780
C +1.20272 -0.52680 -1.83979
C +3.00099 -1.47936 -2.85139
C +3.92297 -2.15484 -3.67005
H +3.57659 -2.75403 -4.50850
C +5.28512 -2.04149 -3.39882
H +6.00425 -2.56005 -4.02815
C +5.74435 -1.25898 -2.32250
H +6.81106 -1.18188 -2.12896
C +4.85074 -0.57440 -1.49665
H +5.21074 +0.02605 -0.67077
C +3.48534 -0.69248 -1.76802
C +0.67913 -1.94331 -3.94212
H +1.25426 -2.60211 -4.60159
H +0.22476 -1.16592 -4.57168
H -0.14463 -2.53171 -3.52534
C +3.31810 -0.34141 +1.56411
C +3.67909 -1.67894 +1.34927
H +3.37279 -2.18725 +0.44275
C +4.43050 -2.36849 +2.30308
H +4.71046 -3.40082 +2.11485
C +4.80945 -1.74336 +3.49342
H +5.3924 -2.2845 +4.23376
C +4.42749 -0.41894 +3.72911
H +4.70644 +0.07463 +4.65623
C +3.68529 +0.27617 +2.77412
H +3.39737 +1.30552 +2.96933
C +2.93285 +2.22169 +0.30498
C +4.31015 +2.46555 +0.44409
H +4.98385 +1.65832 +0.71094
C +4.82565 +3.74949 +0.26153
H +5.89314 +3.92065 +0.37275
C +3.97474 +4.81074 -0.06338
H +4.37891 +5.80945 -0.20596
C +2.60349 +4.58270 -0.19379
H +1.92934 +5.39936 -0.43739
C +2.08899 +3.29908 -0.00413
H +1.02395 +3.13881 -0.08465
C -1.25955 -0.80685 -1.75977
C -2.25960 -0.74418 -2.70725
C -4.12991 -2.45924 -3.31921
H -4.57421 -1.87269 -4.11967
C -3.03113 -1.95930 -2.60120
C -4.63544 -3.71766 -2.99272
H -5.48663 -4.11463 -3.54037
C -2.48436 +0.28254 -3.77530
H -3.54726 +0.54861 -3.84661
H -2.18712 -0.09831 -4.76379
H -1.91902 +1.19767 -3.59263
C -2.95372 -4.01580 -1.24592
H -2.49994 -4.61285 -0.46205
C -1.31316 +3.06173 +1.85730
C -2.45883 -2.75139 -1.56994
C +0.52302 -3.52201 +0.38249
C +1.29572 -3.87186 +1.50273
H +1.17514 -3.33044 +2.43762
C +2.23532 -4.90043 +1.41976
H +2.81976 -5.16568 +2.29610
C +2.44319 -5.56462 +0.20794
H +3.18687 -6.35403 +0.13966
C -2.00730 -2.76208 +1.59188
C -0.84716 +5.07513 +3.76880
H -0.66827 +5.85509 +4.50409
C +0.73370 -4.19624 -0.82987
H +0.15031 -3.93098 -1.70475
C -3.22055 -2.05478 +1.54916
H -3.32866 -1.21843 +0.86698
C -4.28983 -2.42905 +2.36031
H -5.22252 -1.87522 +2.30462
C -0.255 +1.48706 -1.72777
C -4.15850 -3.50718 +3.24186
H -4.99017 -3.79694 +3.87844
C -1.88281 -3.84328 +2.47542
H -0.96324 -4.41642 +2.52133

C -0.85178 +5.93701 -2.83961
H -0.72928 +6.87791 -3.37032
C -0.10594 +4.82470 -3.22638
H +0.59101 +4.89014 -4.05809
C -2.95281 -4.20871 +3.29797
H -2.84184 -5.05000 +3.97717
C -1.77323 +5.85479 -1.77879
H -2.35829 +6.72865 -1.50434
C -0.27046 +3.61364 -2.53231
C +0.29912 +2.29928 -2.69695
C -1.95957 +4.66162 -1.07718
H -2.68900 +4.59836 -0.28020
C -1.18854 +3.55773 -1.44597
C -4.05335 -4.48732 -1.96752
H -4.46062 -5.46759 -1.73419
C +0.16469 +4.16782 +3.44677
H +1.13514 +4.23355 +3.93146
C +1.24368 +1.90269 -3.79517
H +1.42145 +2.74521 -4.47216
H +0.84578 +1.07639 -4.39563
H +2.21888 +1.57994 -3.41043
C -2.09678 +4.96737 +3.15134
H -2.89299 +5.66212 +3.40498
C +1.69617 -5.20407 -0.91727
H +1.85711 -5.71175 -1.86473
C -0.07161 +3.16214 +2.50852
H +0.71042 +2.44647 +2.28040
C -2.32899 +3.96780 +2.20591
H -3.30860 +3.89145 +1.74560
C -3.34864 +1.46867 +0.58996
C -5.40208 +1.18648 -0.67637
H -5.91786 +1.17963 -1.63314
C -6.10541 +0.92721 +0.50231
H -7.17048 +0.71437 +0.46820
C -5.43211 +0.94451 +1.72834
H -5.97188 +0.74673 +2.65053
C -4.03321 +1.46213 -0.63401
H -3.49778 +1.67045 -1.55346
C +0.56458 -0.58466 +3.87170
C +1.05891 -1.09668 +5.13851
H +0.30719 -1.74759 +5.59639
H +1.28052 -0.26689 +5.81716
H +1.97647 -1.66887 +4.96469
C -4.06257 +1.20482 +1.77167
H -3.54541 +1.19855 +2.72795

Ni-TIM_MeCN_q1_s_MN15_6-311++Gdp_forall
E = -5296.43592511,1
Ni +0.02610 +0.05118 +0.73472
P -0.36952 -2.18500 +0.51346
P -1.74062 +1.43166 +0.54155
P +2.18079 +0.76551 +0.59182
C -1.88270 -0.88836 -2.81820
N -1.19021 -2.08857 -1.00899
N -1.38134 +2.06831 -1.03014
N +2.50284 +0.15770 -1.00689
N -0.05783 +0.02182 +2.62424
C +0.03456 +0.06495 -1.27159
C -1.04079 -0.88431 -1.73172
C -2.5749 -2.15709 -2.79771
C -3.53870 -2.73361 -3.64306
H -3.90136 -2.19271 -4.51303
C -4.01845 -4.00693 -3.35108
H -4.76259 -4.46305 -3.99862
C -3.55236 -4.71688 -2.22614
H -3.94229 -5.70981 -2.01940
C -2.59802 -4.16986 -1.37069
H -2.24572 -4.71450 -0.50327
C -2.11629 -2.89234 -1.67143
C -2.03701 +0.14594 -3.89536
H -2.79042 -0.1727 -4.62241
H -1.10049 +0.31044 -4.44051
H -2.35054 +1.11782 -3.50034
C -1.58056 -2.79380 +1.73023
C -2.96029 -2.62175 +1.56052
H -3.35121 -2.19899 +0.64241
C -3.83969 -2.98922 +2.58129
H -4.90775 -2.86053 +2.43478
C -3.35117 -3.50237 +3.78439
H -4.03949 -3.78214 +4.57709
C -1.97258 -3.64944 +3.96881
H -1.58379 -4.04098 +4.90479
C -1.08956 -3.29751 +2.94823
H -0.02047 -3.42125 +3.09734
C +0.83091 -3.56258 +0.33667
C +0.46992 -4.90877 +0.51807
H -0.53575 -5.16917 +0.83012
C +1.40795 -5.92169 +0.31691
H +1.11721 -6.95887 +0.45961
C +2.71505 -5.60479 -0.06564
H +3.44288 -6.39649 -0.22153
C +3.08540 -4.26923 -0.23623
H +4.10067 -4.01034 -0.52354
C +2.14965 -3.25495 -0.03151
H +2.45407 -2.22473 -0.14448
C -0.28029 +1.47402 -1.69451
C +0.17221 +2.31668 -2.67990
C -0.70986 +4.65698 -3.42182
H +0.00997 +4.80390 -4.22269
C -0.67616 +3.48961 -2.64379
C -1.68618 +5.61473 -3.15434
H -1.72544 +6.52469 -3.74716
C +1.23293 +2.12176 -3.71941
H +1.83329 +3.03322 -3.82840

H +0.78342 +1.91792 -4.70121
H +1.90539 +1.29711 -3.48742
C -2.62472 +4.25885 -1.34957
H -3.36653 +4.10147 -0.57554
C +3.36899 -0.01786 +1.75317
C -1.63659 +3.30891 -1.61595
C -3.46140 +0.83918 +0.44928
C -4.10712 +0.45660 +1.63662
H -3.60435 +0.57023 +2.59336
C -5.39670 -0.0718 +1.59275
H -5.89132 -0.35963 +2.51588
C -6.04207 -0.24756 +0.36495
H -7.04212 -0.67107 +0.33227
C -1.79743 +2.87045 +1.67009
C +5.09350 -1.27459 +3.58302
H +5.76169 -1.76058 +4.28871
C -4.11722 +0.67558 -0.77870
H -3.62966 +0.97941 -1.69656
C -0.56807 +3.33949 +2.15468
H +0.34307 +2.82818 +1.87244
C -0.51269 +4.45153 +2.99306
H +0.44942 +4.80641 +3.35176
C +1.39943 -0.39126 -1.70381
C -1.6931 +5.09801 +3.36949
H -1.65531 +5.96098 +4.02875
C -2.98012 +3.52367 +2.05158
H -3.94193 +3.17676 +1.689
C +5.62915 -1.53888 -3.13580
H +6.41046 -2.01297 -3.72381
C +4.29286 -1.79673 -3.42999
H +4.02635 -2.46259 -4.24617
C -2.92403 +4.63056 +2.90000
H -3.84397 +5.13099 +3.18945
C +5.98492 -0.65983 -2.09397
H +7.03446 -0.46225 -1.89364
C +3.29420 -1.17660 -2.6602
C +1.84797 -1.22086 -2.70482
C +5.01396 -0.02805 -1.31839
H +5.29024 +0.66366 -0.53288
C +3.67572 -0.30739 -1.60459
C -2.63234 +5.41550 -2.13041
H -3.39012 +6.17247 -1.94721
C +3.83453 -1.81933 +3.32067
H +3.51467 -2.72941 +3.82044
C +1.06395 -2.02198 -3.70409
H +1.74105 -2.61648 -4.32521
H +0.47722 -1.38598 -4.37522
H +0.36983 -2.71593 -3.21908
C +5.48817 -0.09697 +2.94233
H +6.4631 +0.33664 +3.14678
C -5.39985 +0.12498 -0.81865
H -5.89707 -0.00637 -1.77585
C +2.97548 -1.19177 +2.41843
H +1.99891 -1.62136 +2.23335
C +4.63195 +0.53026 +2.03723
H +4.94884 +1.45056 +1.55841
C +2.71660 +2.51019 +0.51296
C +3.20718 +4.51870 -0.75586
H +3.36494 +5.00485 -1.71486
C +3.28902 +5.25580 +0.42755
H +3.50641 +6.31981 +0.39365
C +3.09826 +4.61865 +1.65783
H +3.17149 +5.18236 +2.58370
C +2.92449 +3.15149 -0.71634
H +2.87599 +2.58497 -1.63818
C -0.15789 -0.08729 +3.77081
C -0.27474 -0.24272 +5.20764
H -0.61696 +0.6946 +5.65765
H +0.70087 -0.51236 +5.62541
H -0.99461 -1.03808 +5.42716
C +2.81363 +3.25428 +1.70239
H +2.66527 +2.76910 +2.66368

Ni-TIM_none_q-1_s_MN15-bigbase
E = -5164.0115648
-1,1
Ni +0.01684 +0.05084 -0.83257
P -2.07223 -0.29258 -0.75710
P +1.31573 -1.60893 -0.82070
P +0.76752 +2.02417 -0.78701
C -1.15063 -1.86586 +2.71385
N -2.19084 -0.99085 +0.88033
N +1.96828 -1.47044 +0.82841
N +0.29598 +2.46374 +0.88751
C +0.05043 +0.01311 +1.32062
C -1.01375 -0.96647 +1.67399
C -2.47153 -2.43357 +2.61357
C -3.18653 -3.35296 +3.40159
H -2.72234 -3.80394 +4.27561
C -4.49861 -3.67204 +3.0559
H -5.05823 -4.38078 +3.66169
C -5.11267 -3.08311 +1.93354
H -6.13784 -3.34342 +1.68238
C -4.42733 -2.16473 +1.13627
H -4.90427 -1.71409 +0.27493
C -3.10947 -1.8485 +1.47963
C -0.17654 -2.14853 +3.82124
H -0.59373 -2.87879 +4.52354
H +0.07301 -1.24813 +4.39689
H +0.77141 -2.55452 +3.44911
C -2.84648 -1.56146 -1.84752
C -2.86553 -2.93254 -1.55861
H -2.48758 -3.29465 -0.60992
C -3.36322 -3.84739 -2.49105

H	-3.37491	-4.90532	-2.24385
C	-3.82723	-3.41217	-3.73298
H	-4.21215	-4.12682	-4.45564
C	-3.78239	-2.04835	-4.04493
H	-4.13087	-1.69717	-5.01295
C	-3.29368	-1.13429	-3.11316
H	-3.26873	-0.07648	-3.36460
C	-3.42749	+0.97821	-0.63268
C	-4.77552	+0.74744	-0.95392
H	-5.08559	-0.20956	-1.36028
C	-5.73639	+1.74498	-0.77036
H	-6.77319	+1.54442	-1.02875
C	-5.37012	+2.99135	-0.25516
H	-6.11921	+3.76583	-0.11209
C	-4.03271	+3.23552	+0.06660
H	-3.72790	+4.20140	+0.46139
C	-3.07468	+2.23995	-0.12737
H	-2.03949	+2.45351	+0.09653
C	+1.42270	-0.45165	+1.65402
C	+2.31118	-0.17701	+2.67368
C	+4.59974	-1.28435	+3.26787
H	+4.79977	-0.68339	+4.15210
C	+3.43355	-1.06943	+2.51517
C	+5.48890	-2.28391	+2.87098
H	+6.39511	-2.45886	+3.44598
C	+2.15414	+0.76600	+3.82767
H	+3.01897	+1.43898	+3.91142
H	+2.08365	+0.22529	+4.78295
H	+1.25968	+1.38352	+3.72833
C	+4.06500	-2.89047	+0.98013
H	+3.85812	-3.51121	+0.11515
C	+0.07929	+3.36277	-1.88005
C	+3.18299	-1.88013	+1.37142
C	+0.70335	-3.34929	-0.80901
C	+0.20424	-3.90762	-1.99753
H	+0.20224	-3.32053	-2.91286
C	-0.30687	-5.20607	-2.01651
H	-0.67966	-5.62315	-2.94793
C	-0.37315	-5.95164	-0.83657
H	-0.78834	-6.95592	-0.84713
C	+2.90472	-1.73718	-1.77529
C	-1.06692	+5.20848	-3.68007
H	-1.50844	+5.92134	-4.37164
C	+0.63255	-4.10647	+0.37129
H	+1.00028	-3.68851	+1.30201
C	+3.84053	-0.70438	-1.59009
H	+3.63539	+0.07975	-0.86985
C	+5.03896	-0.68647	-2.30149
H	+5.74848	+0.11827	-2.13146
C	-0.27211	+1.41938	+1.67221
C	+5.32336	-1.69568	-3.22733
H	+6.25567	-1.68077	-3.78567
C	+3.20426	-2.74997	-2.69880
H	+2.51967	-3.57634	-2.85339
C	-1.19358	+5.78280	+2.90363
H	-1.61762	+6.61461	+3.46095
C	-1.52268	+4.47446	+3.25736
H	-2.19085	+4.28127	+4.09317
C	+4.40037	-2.72445	-3.42289
H	+4.61168	-3.52017	-4.13306
C	-0.30325	+6.03897	+1.84449
H	-0.04139	+7.06447	+1.59650
C	-0.97063	+3.40675	+2.52908
C	-1.05988	+1.97452	+2.66269
C	+0.26331	+4.99422	+1.11018
H	+0.97172	+5.19966	+0.31956
C	-0.09653	+3.68504	+1.43766
C	+5.22308	-3.07815	+1.73924
H	+5.92595	-3.85598	+1.45148
C	-1.68235	+3.97494	-3.45176
H	-2.60507	+3.71880	-3.96621
C	-1.82173	+1.26203	+3.74280
H	-2.23316	+1.98022	+4.46070
H	-1.18706	+0.56637	+4.30233
H	-2.66193	+0.67974	+3.34378
C	+0.13111	+5.51208	-3.02766
H	+0.62812	+6.46127	-3.21127
C	+0.08628	-5.39209	+0.35899
H	+0.02920	-5.95956	+1.28455
C	-1.10304	+3.0585	-2.57343
H	-1.56313	+2.08670	-2.42348
C	+0.69806	+4.59817	-2.13715
H	+1.63823	+4.84849	-1.65708
C	+2.55220	+2.47928	-0.73537
C	+4.64146	+2.72020	+0.48689
H	+5.17181	+2.74947	+1.43554
C	+5.33979	+2.87981	-0.71249
H	+6.41571	+3.03282	-0.70402
C	+4.64418	+2.84155	-1.92569
H	+5.17831	+2.96310	-2.86451
C	+3.2571	+2.53028	+0.47667
H	+2.72426	+2.41012	+1.41357
C	+3.26539	+2.63190	-1.93686
H	+2.73955	+2.58459	-2.88728

Ni-TIM_none_q0_d_MN15-bigbase

E = -5163.9012091

0,2

Ni	+0.01479	+0.04422	-0.86386
P	-2.17908	-0.26651	-0.70530
P	+1.33861	-1.71933	-0.77799
P	+0.84928	+2.08730	-0.74115
C	-1.11410	-1.89825	+2.67210
N	-2.20728	-1.01614	+0.87423

N	+1.96435	-1.49137	+0.83670
N	+0.31275	+2.45853	+0.89472
C	+0.0393	+0.00307	+1.27233
C	-1.01024	-0.98791	+1.64323
C	-2.42516	-2.49381	+2.58370
C	-3.10025	-3.44656	+3.36648
H	-2.60765	-3.90251	+4.22165
C	-4.40827	-3.79392	+3.03635
H	-4.93816	-4.52914	+3.63683
C	-5.05710	-3.20147	+1.93571
H	-6.07892	-3.48454	+1.69706
C	-4.41101	-2.25217	+1.14317
H	-4.91442	-1.80168	+0.29707
C	-3.09727	-1.90884	+1.47388
C	-0.12029	-2.17055	+3.76427
H	-0.51575	-2.91213	+4.46658
H	+0.11582	-1.26705	+4.34005
H	+0.82957	-2.55655	+3.37734
C	-2.90444	-1.47887	-1.87018
C	-2.88367	-2.86232	-1.64966
H	-2.50030	-3.26307	-0.71918
C	-3.35536	-3.73743	-2.6311
H	-3.34223	-4.80653	-2.44028
C	-3.82543	-3.24670	-3.85040
H	-4.18941	-3.93180	-4.61124
C	-3.81671	-1.86895	-4.09115
H	-4.17077	-1.47727	-5.04102
C	-3.35882	-0.99070	-3.10967
H	-3.36617	+0.07910	-3.30274
C	-3.48784	+1.02879	-0.56721
C	-4.85078	+0.79102	-0.81183
H	-5.18367	-0.17995	-1.16312
C	-5.79237	+1.80429	-0.62451
H	-6.84253	+1.60363	-0.81965
C	-5.38842	+3.06913	-0.18674
H	-6.12384	+3.85573	-0.04011
C	-4.03529	+3.31929	+0.05087
H	-3.70536	+4.30012	+0.38246
C	-3.09384	+2.30800	-0.14532
H	-2.04744	+2.52323	+0.01582
C	+1.41488	-0.44109	+1.62537
C	+2.30480	-0.12064	+2.62596
C	+4.61682	-1.16648	+3.23714
H	+4.81425	-0.52839	+4.09503
C	+3.43961	-1.00319	+2.48921
C	+5.52039	-2.16215	+2.86680
H	+6.43601	-2.29812	+3.43695
C	+2.14643	+0.85460	+3.75229
H	+3.02572	+1.50754	+3.83505
H	+2.04712	+0.33581	+4.71667
H	+1.26739	+1.48785	+3.62356
C	+4.09133	-2.86563	+1.01410
H	+3.88736	-3.52121	+0.17464
C	+0.14579	+3.35040	-1.88421
C	+3.19586	-1.85899	+1.38175
C	+0.68869	-3.43227	-0.74017
C	+0.15216	-3.97953	-1.91681
H	+0.14622	-3.39735	-2.8351
C	-0.38850	-5.26595	-1.91489
H	-0.79263	-5.68029	-2.83400
C	-0.43869	-6.00378	-0.7293
H	-0.87594	-6.99854	-0.72464
C	+2.87973	-1.82536	-1.78502
C	-1.03061	+5.11319	-3.73386
H	-1.48622	+5.79779	-4.44427
C	+0.63957	-4.17980	+0.44600
H	+1.04222	-3.76529	+1.36362
C	+3.76565	-0.73942	-1.68674
H	+3.53994	+0.07910	-1.01224
C	+4.94400	-0.71521	-2.43042
H	+5.61960	+0.12969	-2.33394
C	-0.29444	+1.40423	+1.64017
C	+5.25018	-1.77128	-3.29455
H	+6.16705	-1.75173	-3.87748
C	+3.19598	-2.88420	-2.64732
H	+2.54130	-3.74477	-2.72958
C	-1.23864	+5.75317	+2.89467
H	-1.68088	+6.57949	+3.44549
C	-1.58169	+4.44261	+3.22336
H	-2.28015	+4.24153	+4.03165
C	+4.37285	-2.85203	-3.40121
H	+4.60515	-3.68076	-4.06493
C	-0.31206	+6.02112	+1.86964
H	-0.04121	+7.04908	+1.64365
C	-1.00556	+3.38303	+2.50238
C	-1.10834	+1.94731	+2.61106
C	+0.27744	+4.98542	+1.14227
H	+1.01205	+5.19572	+0.37653
C	-0.09642	+3.67572	+1.44857
C	+5.25915	-3.00261	+1.76778
H	+5.97416	-3.77671	+1.50171
C	-1.60123	+3.85951	-3.50290
H	-2.50019	+3.55890	-4.03417
C	-1.90494	+1.22366	+3.65862
H	-2.39456	+1.93824	+4.32863
H	-1.27386	+0.57443	+4.27632
H	-2.68761	+0.59219	+3.22211
C	+0.13906	+5.47856	-3.06064
H	+0.59799	+6.4455	-3.24839
C	+0.06725	-5.45354	+0.45172
H	+0.02447	-6.01811	+1.37943
C	-1.00935	+2.98189	-2.59393
H	-1.44415	+2.00115	-2.42920
C	+0.72565	+4.60326	-2.14622
H	+1.64596	+4.89358	-1.65064
C	+2.62546	+2.52767	-0.66389

C +4.68612 +2.77942 +0.59597
H +5.19819 +2.82135 +1.55378
C +5.40675 +2.91926 -0.59251
H +6.48266 +3.06900 -0.56517
C +4.73651 +2.86725 -1.81916
H +5.28908 +2.97707 -2.74838
C +3.30264 +2.59054 +0.56253
H +2.75028 +2.48678 +1.48935
C +3.35705 +2.66391 -1.85603
H +2.84947 +2.60658 -2.81549

Ni-TIM_none_q1_s_MN15_6-311++gdp
E = -5163.7492121
1,1
Ni +0.01331 +0.00845 -0.79446
P -2.22586 -0.21923 -0.66124
P +1.30918 -1.81796 -0.74160
P +0.94159 +2.07386 -0.71508
C -1.02274 -1.95787 +2.59713
N -2.20762 -1.07028 +0.85618
N +1.94653 -1.52937 +0.84968
N +0.32701 +2.42870 +0.88904
C +0.02523 -0.02283 +1.17372
C -0.98427 -1.05041 +1.56175
C -2.33184 -2.56249 +2.55826
C -2.95400 -3.53710 +3.35893
H -2.42299 -3.98035 +4.19673
C -4.25557 -3.92327 +3.05998
H -4.74846 -4.67535 +3.67007
C -4.94824 -3.35235 +1.97202
H -5.9643 -3.67199 +1.75743
C -4.35781 -2.38439 +1.16387
H -4.89091 -1.95747 +0.32397
C -3.05132 -1.99167 +1.47264
C +0.01258 -2.21909 +3.65067
H -0.33768 -2.98681 +4.34711
H +0.22877 -1.31569 +4.23334
H +0.96146 -2.56326 +3.22758
C -2.94636 -1.35434 -1.89117
C -2.87000 -2.74674 -1.75153
H -2.43531 -3.18553 -0.86174
C -3.35847 -3.57719 -2.76167
H -3.30561 -4.65463 -2.63706
C -3.89957 -3.02847 -3.92631
H -4.27598 -3.67908 -4.71090
C -3.95045 -1.63987 -4.08282
H -4.36391 -1.20616 -4.98912
C -3.47789 -0.80404 -3.07148
H -3.53431 +0.27395 -3.19539
C -3.49376 +1.09373 -0.46855
C -4.87233 +0.83113 -0.54988
H -5.22697 -0.16595 -0.78766
C -5.79722 +1.85484 -0.34503
H -6.86026 +1.63917 -0.40869
C -5.35884 +3.15222 -0.06083
H -6.08200 +3.9475 +0.09845
C -3.99140 +3.42482 +0.00861
H -3.63952 +4.43035 +0.22123
C -3.06556 +2.40157 -0.19847
H -2.01029 +2.62818 -0.15835
C +1.41547 -0.41348 +1.54134
C +2.29004 -0.01691 +2.52757
C +4.59671 -1.01736 +3.21752
H +4.78565 -0.32500 +4.03341
C +3.41810 -0.91667 +2.45960
C +5.51098 -2.01809 +2.90212
H +6.42946 -2.10958 +3.47549
C +2.14226 +1.03121 +3.58538
H +3.06352 +1.61950 +3.67305
H +1.96613 +0.56770 +4.56563
H +1.31780 +1.71537 +3.38738
C +4.09656 -2.84930 +1.08798
H +3.90208 -3.54905 +0.28257
C +0.24386 +3.28033 -1.90777
C +3.18290 -1.84197 +1.41061
C +0.64701 -3.51641 -0.68313
C +0.08267 -4.05872 -1.84929
H +0.06988 -3.48164 -2.77021
C -0.47956 -5.33536 -1.82863
H -0.90674 -5.74858 -2.73759
C -0.51573 -6.06645 -0.63810
H -0.96835 -7.05401 -0.61990
C +2.81178 -1.89871 -1.78882
C -0.90153 +5.00485 -3.80473
H -1.34666 +5.67583 -4.53432
C +0.61580 -4.25684 +0.50760
H +1.04498 -3.84617 +1.41392
C +3.67587 -0.79322 -1.72457
H +3.44502 +0.03515 -1.06441
C +4.84204 -0.76452 -2.48659
H +5.50276 +0.09493 -2.42099
C -0.35163 +1.36861 +1.54311
C +5.15414 -1.83602 -3.32886
H +6.06192 -1.81357 -3.92548
C +3.13205 -2.97449 -2.62878
H +2.49057 -3.84714 -2.68067
C -1.29926 +5.66036 +2.91774
H -1.75851 +6.47372 +3.47281
C -1.66627 +4.34637 +3.18933
H -2.40497 +4.12413 +3.95433
C +4.29784 -2.93720 -3.39780
H +4.53854 -3.77668 -4.04432
C -0.32714 +5.95323 +1.93929
H -0.04327 +6.98658 +1.75990

C	-1.06331	+3.30807	+2.45807
C	-1.21055	+1.87232	+2.49477
C	+0.28815	+4.94289	+1.20337
H	+1.05225	+5.16992	+0.47140
C	-0.10114	+3.62618	+1.46272
C	+5.26210	-2.92234	+1.84854
H	+5.99133	-3.69662	+1.62658
C	-1.44135	+3.73266	-3.60300
H	-2.30413	+3.40482	-4.17600
C	-2.11997	+1.12999	+3.42681
H	-2.55850	+1.81425	+4.15915
H	-1.59046	+0.34608	+3.97612
H	-2.94408	+0.65007	+2.88505
C	+0.22169	+5.40937	-3.07682
H	+0.65393	+6.39250	-3.24096
C	+0.02640	-5.52227	+0.52921
H	-0.00333	-6.08348	+1.45915
C	-0.86774	+2.87325	-2.66582
H	-1.28916	+1.88383	-2.51913
C	+0.79634	+4.55185	-2.13921
H	+1.68303	+4.86916	-1.60092
C	+2.70022	+2.54683	-0.59255
C	+4.69536	+2.99363	+0.71243
H	+5.16675	+3.14651	+1.67956
C	+5.45714	+3.04639	-0.45678
H	+6.52540	+3.23761	-0.40409
C	+4.83987	+2.85716	-1.69774
H	+5.42501	+2.90231	-2.61200
C	+3.32190	+2.74840	+0.64742
H	+2.73538	+2.72367	+1.55776
C	+3.47031	+2.60405	-1.76704
H	+3.00189	+2.44523	-2.73481

6. References

[1] Michaliszyn, K.; Smirnova, E. S.; Bucci, A.; Martin-Diaconescu, V.; Lloret-Fillol, J., Well-defined Nickel P_3C Complexes as Hydrogenation Catalysts of N-Heteroarenes Under Mild Conditions. **2022,** *14*, e202200039.

[2] Savéant, J.-M. Elements of Molecular and Biomolecular Electrochemistry; John Wiley & Sons, Inc.: Hoboken, NJ, USA, **2006**. https://doi.org/10.1002/0471758078.

[3] Nicholson, R. S.; Shain, I. Theory of Stationary Electrode Polarography. Single Scan and Cyclic Methods Applied to Reversible, Irreversible, and Kinetic Systems. *Anal. Chem.* **1964**, *36* (4), 706–723. https://doi.org/10.1021/ac60210a007.

[4] Nicholson, R. S. Semiempirical Procedure for Measuring with Stationary Electrode Polarography Rates of Chemical Reactions Involving the Product of Electron Transfer. *Anal. Chem.* **1966**, *38* (10), 1406–1406. https://doi.org/10.1021/ac60242a030.

[5]a) Gaussian 09, Revision E.01, Frisch et. al. Gaussian, Inc., Wallingford CT, **2009**. b) Gaussian 16, Revision C.01, Frisch et. al. Gaussian, Inc., Wallingford CT, **2016**.

[6]A. V. Marenich, C. J. Cramer, D. G. Truhlar, *J. Phys. Chem. B*, **2009**, *113*, 6378.

[7]S. Grimme, J. Antony, S. Ehrlich, H. J. Krieg, *Chem. Phys.* **2010**, *132*, 154104.

[8]D. G. Truhlar, C. J. Cramer, A. Lewis, J. A. Bumpus, *J. Chem. Educ.* **2004**, *81*, 596.

[9]V. V. Pavlishchuk, A. W. Addison, *Inorganica Chimica Acta*, **2000**, *298*, 97.
